# Transmission Of Tuberculosis Among illicit drug use Linkages (TOTAL): A cross-sectional observational study protocol using respondent driven sampling

**DOI:** 10.1371/journal.pone.0262440

**Published:** 2022-02-15

**Authors:** Tara Carney, Jennifer A. Rooney, Nandi Niemand, Bronwyn Myers, Danie Theron, Robin Wood, Laura F. White, Christina S. Meade, Novel N. Chegou, Elizabeth Ragan, Gerhard Walzl, Robert Horsburgh, Robin M. Warren, Karen R. Jacobson

**Affiliations:** 1 Alcohol, Tobacco and Other Drug Research Unit, South African Medical Research Council, Tygerberg, South Africa; 2 Division of Addiction Psychiatry, Department of Psychiatry and Mental Health, University of Cape Town, Groote Schuur Hospital, Observatory, Cape Town, South Africa; 3 Section of Infectious Diseases, Department of Medicine, Boston University School of Medicine, Boston, MA, United States of America; 4 DSI-NRF Centre of Excellence for Biomedical Tuberculosis Research and South African Medical Research Council Centre for Tuberculosis Research, Division of Molecular Biology and Human Genetics, Faculty of Medicine and Health Sciences, Stellenbosch University, Cape Town, South Africa; 5 Curtin enAble Institute, Faculty of Health Sciences, Curtin University, Perth, Australia; 6 Brewelskloof Hospital, Worcester, South Africa; 7 Desmond Tutu Health Foundation, UCT Faculty of Health Sciences, Observatory, Cape Town, South Africa; 8 Department of Biostatistics, Boston University School of Public Health, Boston, MA, United States of America; 9 Department of Psychiatry and Behavioral Sciences, Duke University School of Medicine, Durham, NC, United States of America; 10 Department of Epidemiology, Biostatistics and Global Health, Boston University School of Public Health, Boston, MA, United States of America; Public Library of Science, UNITED KINGDOM

## Abstract

People who use illicit drugs (PWUDs) have been identified as a key at-risk group for tuberculosis (TB). Examination of illicit drug use networks has potential to assess the risk of TB exposure and disease progression. Research also is needed to assess mechanisms for accelerated TB transmission in this population. This study aims to 1) assess the rate of TB exposure, risk of disease progression, and disease burden among PWUD; 2) estimate the proportion of active TB cases resulting from recent transmission within this network; and 3) evaluate whether PWUD with TB disease have physiologic characteristics associated with more efficient TB transmission. Our cross-sectional, observational study aims to assess TB transmission through illicit drug use networks, focusing on methamphetamine and Mandrax (methaqualone) use, in a high TB burden setting and identify mechanisms underlying accelerated transmission. We will recruit and enroll 750 PWUD (living with and without HIV) through respondent driven sampling in Worcester, South Africa. Drug use will be measured through self-report and biological measures, with sputum specimens collected to identify TB disease by Xpert Ultra (Cepheid) and mycobacterial culture. We will co-enroll those with microbiologic evidence of TB disease in Aim 2 for molecular and social network study. Whole genome sequencing of *Mycobacteria tuberculosis* (*Mtb*) specimens and social contact surveys will be done for those diagnosed with TB. For Aim 3, aerosolized *Mtb* will be compared in individuals with newly diagnosed TB who do and do not smoke illicit drug. Knowledge from this study will provide the basis for a strategy to interrupt TB transmission in PWUD and provide insight into how this fuels overall community transmission. Results have potential for informing interventions to reduce TB spread applicable to high TB and HIV burden settings.

**Trial registration:** Clinicaltrials.gov Registration Number: NCT041515602. Date of Registration: 5 November 2019.

## Introduction

Tuberculosis (TB) is one of the leading infectious diseases globally that cause death, including among people living with HIV (PLHIV) [1]. An effective method to reduce TB incidence and mortality is to interrupt transmission, which requires finding and treating individuals with subclinical and clinical TB disease early. Molecular epidemiologic studies and mathematical models have shown that household contact tracing, our primary approach to case finding, identifies less than 19% of transmissions in high TB and TB/HIV burden settings [2]. There is a clear need to identify additional risk groups and settings where TB transmission occurs. New approaches have focused on congregate settings, such as prisons and healthcare facilities, to identify additional TB transmission [3]. A major source of transmission also may exist in venues where individuals smoke illicit drugs, making these social networks potential amplifiers for the broader population epidemic and a group to target for transmission interventions.

Globally, people who use drugs (PWUD) have higher TB infection prevalence and disease incidence compared to people who do not use drugs, likely reflecting a combination of within-group transmission and clustered vulnerability [4]. In the US, nearly one third of persons ≥ 15 years old with TB disease reported problem alcohol or drug use [5], and the majority of US-based outbreaks between 2002 and 2015 were among PWUD. TB outbreaks among PWUD have also been reported in other countries [6–11]. In the past 20 years, TB incidence has decreased in most high-income countries with TB disease burden concentrated in at-risk subpopulations, including PWUD [12]. Similar higher rates of TB disease among PWUD may occur in high TB burden settings as well, reflecting increased transmission within these groups, but the phenomenon may be harder to isolate given the general high population burden.

South Africa is an ideal context to study the relationship between drug use and TB due to its concurrent high TB/HIV burden and high drug use prevalence [13, 14]. Nationally, an estimated 13.3% of the adult population meet criteria for a lifetime substance use disorder (SUD), with rates as high as 20.6% for the Western Cape Province [14]. Community-based data related to illicit drug use in South Africa are limited but suggest a growing problem. A 2012 population-based study of adults found the past 3-month prevalence was 4.0% for cannabis, 0.4% for sedatives, and 0.3% for amphetamines [13]. A 2017 national study estimated the prevalence of any drug use in the last 3 months increased to 8.6% of adults, largely driven by cannabis use followed by Mandrax (methaqualone) and stimulants such as methamphetamine [15]. Polysubstance use is common [16, 17], and likely to be under-reported due to the illicit nature of substance use and concerns about stigma. In the Western Cape, methamphetamine (referred to as tik), which is generally smoked, has been a popular self-reported drug of choice among patients entering SUD treatment for a number of years.

PWUD, particularly those who smoke drugs, may be especially efficient TB transmitters, due to the sharing of pipes and other drug equipment, and time spent in close, poorly ventilated settings [6]. Physiological effects of smoked drugs induce repeated coughing and lung trauma, which likely facilitates TB spread [18, 19]. Illicit drug use also frequently co-occurs with other TB disease risk factors, including tobacco and alcohol use, HIV, and environmental risk factors such as homelessness, malnutrition, and incarceration history [6, 20–23]. Symptoms of regular drug use, including chronic cough, weight loss, and sweats, also often overlap with TB symptoms, leading to delayed evaluation [24, 25]. In addition, PWUD often wait longer to present for treatment due to perceived stigma or fear of withdrawal, especially if hospitalized [26]. Routine TB investigations and contact tracing may miss links between PWUD due to underreporting of relationships which involve drug use or sales [27]. Hereafter, PWUD refers to those who smoke illicit drugs (rather than inject or ingest).

Respondent Driven Sampling (RDS), developed to understand and address infectious disease epidemics in hard-to-reach or hidden populations [28, 29], may be effective for recruiting PWUD into community-based TB studies and provide opportunities to understand social dynamics of TB transmission. RDS uses coupons with unique numbers to track recruitment, incentives for survey participation and for recruiting others into study activities and is initiated by purposively selecting initial members of the target population, known as *seeds*. A variant of chain referral sampling, RDS has the advantage of using statistical estimation methods to limit biases from peer-driven recruitment [29]. While RDS has successfully reached and recruited PWUD [30, 31] to understand drug use, involvement in illegal activities, and HIV risk behaviours in Cape Town’s peri-urban communities, it has not been widely utilised in rural settings or for TB transmission studies. In preparation for RDS, formative work was conducted with eight focus groups with participants from the study’s target population, namely, individuals who self-reported current methamphetamine and/or Mandrax use, to obtain recommendations for recruitment, confirm whether RDS would be a feasible strategy and identify potential seeds.

### Primary objectives

The Transmission Of Tuberculosis Among illicit drug use Linkages (TOTAL) study has three aims. The first is to assess the TB exposure, risk of disease progression, and disease burden among PWUD, including people living with and without HIV. We hypothesize that TB prevalence in PWUD will be significantly higher compared to results from a community-wide survey [32] and that the prevalence of incipient TB (host RNA signature presence) will be significantly higher among PWUD compared to the general community. We also hypothesize that PWUD living with HIV will have higher rates of incipient, subclinical, and clinical TB disease compared to those PWUD who are HIV negative.

Second, we aim to estimate the proportion of active TB cases that are the result of transmission within the social network of PWUD. We hypothesize that there will be a high proportion of linked isolates from Aim 1, reflecting predominance of recent transmission within this cohort. We also expect that persons with TB/HIV co-infection will more often appear as contacts with fewer links compared to persons with TB disease alone, reflecting their increased susceptibility to TB and their decreased ability to transmit TB.

Third, we aim to evaluate whether PWUD with TB disease have physiologic characteristics associated with more efficient TB transmission compared to people with TB disease who do not use drugs. Among individuals with TB, we hypothesize that PWUD will produce larger amounts of aerosolized *Mtb* than people who do not use drugs, after adjusting for bacterial burden (tested by mycobacterial culture time to positivity and GeneXpert Ultra). We will include people with and without HIV in each arm to assess any modifying effects of HIV, and record other smoked substances (tobacco, cannabis) and alcohol use to assess their impact as well.

## Methods

**Fig 1:**
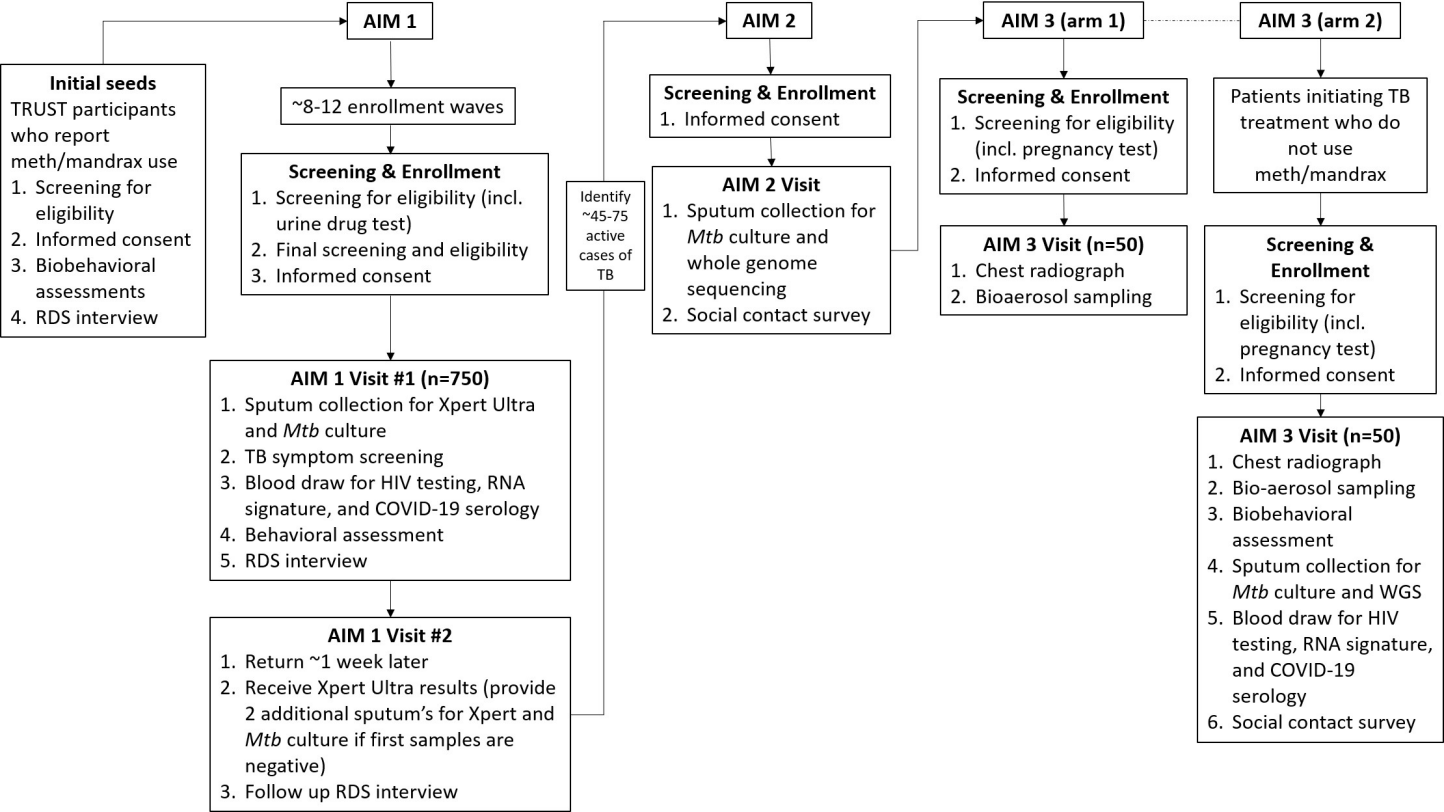
Study design.

TOTAL is a cross-sectional, observational study. The protocol for this study was developed using the Strengthening the Reporting of Observational Studies in Epidemiology (STROBE) guidelines [33] and this checklist can be found in S1 Checklist. For Aim 1, RDS will be used to recruit 750 people who use methamphetamine and/or Mandrax. For Aim 2, we will screen and enroll those participants from Aim 1 with TB disease based on positive microbiology (projected 45–75 participants) to estimate the proportion that reflect recent TB transmission via Whole Genome Sequencing (WGS) and social epidemiologic links. For Aim 3, we will enroll 100 participants who are diagnosed with TB and compare PWUD (Arm 1) with people who do not use drugs (Arm 2). For Arm 1 we will recruit 50 participants, most from Aim 2, who have active, untreated pulmonary TB and use methamphetamine and/or Mandrax. For Arm 2 we will recruit 50 persons who do not smoke illicit drugs but have been newly diagnosed with TB disease and have received less than five days of treatment. The study design is shown in Fig 1.

The protocol for this study has been approved by the following health research ethics committees: Stellenbosch University (primary institution: N19/10/128), South African Medical Research Council (defers to primary institution of approval) and University of Cape Town (604/2020) and Boston Medical Center Institutional Review Board (H-38910). All participants in this study are informed about the study procedures and agree to participate in this study and informed consent is obtained from all participants. For participants under 18 years old, parental consent will first be obtained prior to informed consent. All methods were performed in accordance with the relevant guidelines and regulations.

### Study site selection

We will recruit participants residing in the greater Worcester area. Worcester is the main town of the Breede Valley municipality in the Western Cape Province, South Africa, and is a prominent wine producing region [34] with high rates of TB and HIV. Though the prevalence of methamphetamine and Mandrax use in this community is not precisely known, both drugs are widely available as reflected in reports from local SUD treatment providers [16]. We recently found that among persons initiating TB treatment and recruited into a prospective research study in the Worcester Community Day Centre (CDC), 53% reported smoked drug use including methamphetamines, Mandrax, and cannabis [35].

### Participant eligibility criteria

**Fig 2:**
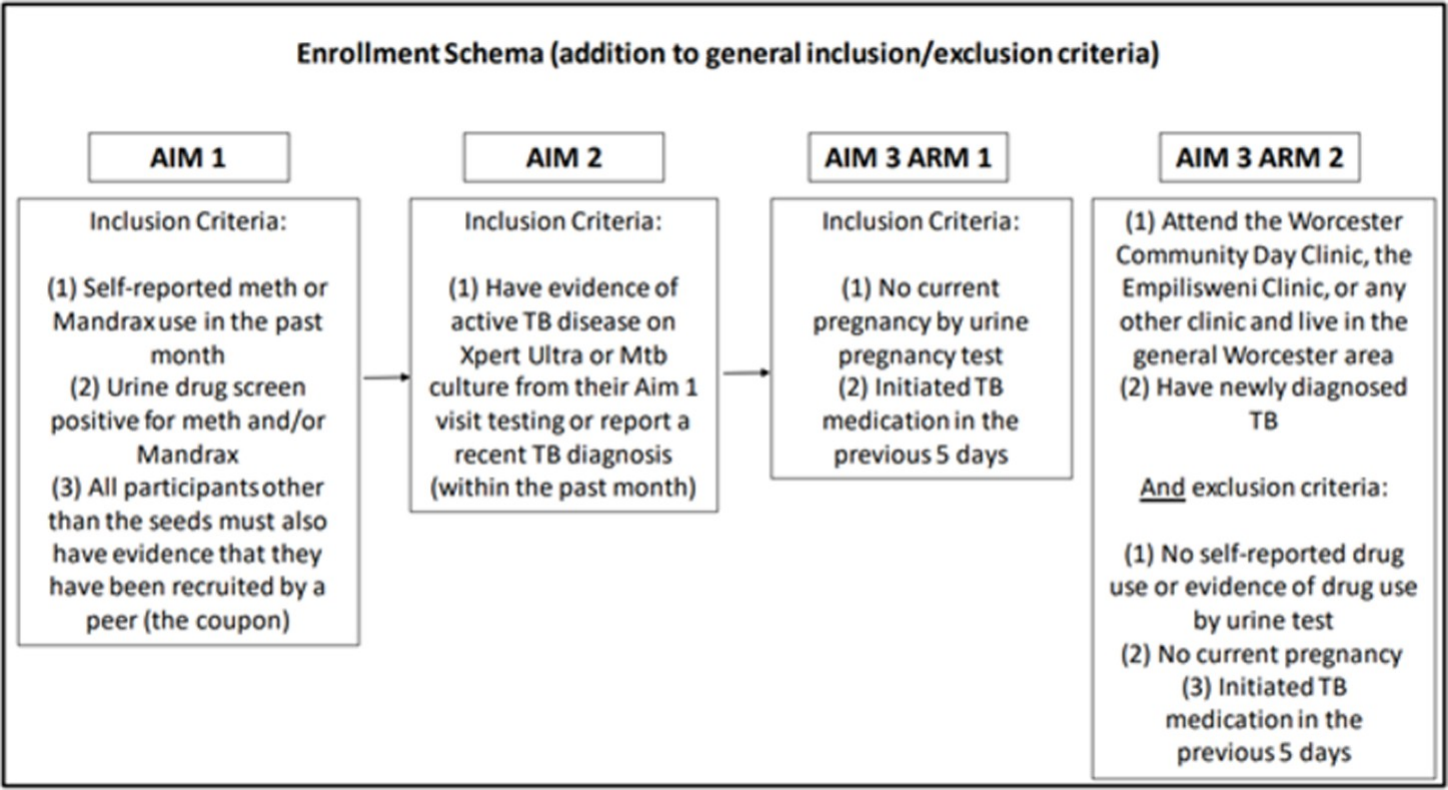
TOTAL participant inclusion criteria per study arm.

Study participants will be recruited by referral chains of PWUD that start with seeds who will be ≥15 years old, reside locally, are willing and able to provide written informed consent and participate in study activities. Inclusion criteria by study arm in shown in Fig 2. For Aim 1, additional inclusion criteria will include self-reported methamphetamine or Mandrax use in the past month and a positive urine drug screen for either of these drugs at enrollment, and a coupon indicating their recruitment into the study by another participant (with the exception of seeds). For Aims 2 and 3, individuals will have microbiologic evidence of active TB disease Xpert Ultra (Cepheid, CA) or *Mtb* culture]. For Aim 3, participants will be excluded if they are pregnant, have taken >5 days of TB medication, or for the control group in Aim 3 Arm 2, if they self-report any past month drug use or have positive urine drug test results. Exclusion criteria for all study aims will include current alcohol or drug intoxication and inability to provide informed consent.

### Recruitment and assessment of primary objectives

For Aim 1, we will invite eligible participants to enroll as initial study participants (seeds).

Individuals will provide written informed consent (and parental consent will be obtained for individuals <18 years) before being screened for Aim 1 eligibility. Enrollment will include the collection of biometric fingerprints. Seeds will then complete interviewer-administered biobehavioural questionnaires and provide blood for COVID-19 serology, rapid HIV testing, and host RNA sequencing, and induced sputa for Xpert Ultra and mycobacterial culture respectively. Those with a new HIV diagnosis will be referred to a clinic so that they can access HIV care services. For the latter, sputum will be liquefied and decontaminated (N-Acetyl-L-Cysteine) and neutralized in PBS. The bacilli will be collected by centrifugation, with positive MGIT cultures assessed for acid fast bacilli and Capilia TB Testing to confirm the *Mtb* presence.

Each seed will then be given two coupons to recruit two people with whom they currently use methamphetamine and/or Mandrax. The initial recruits who are successfully enrolled into the study will be given two recruitment coupons to disperse in the same manner. This will continue until we reach 750 participants. Additional seeds will be included as needed until we reach the target sample size (n = 750).

Participants will be asked to return approximately two weeks after their first visit to receive their Xpert Ultra. Detailed contact information will be obtained from participants in order to be able to contact them. If any of the coupon that they disperse result in successful enrollments, they will receive an additional grocery voucher to reimburse them for these efforts. At this time, they will also complete their second RDS interview (to track number of coupons handed out to date). If a participant’s specimen has negative initial TB culture results, we will ask for two additional sputum samples. If the Xpert test is positive and we believe the participant has current TB disease (has not been treated for active TB disease in prior two years which would cause a false positive test results), we will ask them to return to the study site and refer and transport them to their local clinic for confirmation testing and treatment initiation. If the culture results come back as positive at a later stage, participants will be contacted, tracked in the community and asked to come in for a TB result visit. Participants from Aim 1 with evidence of TB disease (i.e., positive Xpert Ultra results and/or positive *Mtb* culture) will be asked to consent to enroll into Aim 2 in which we will estimate the proportion of participants that reflect recent within-network transmission via WGS and social epidemiologic links. Study staff will conduct interviews to identify individual’s social contacts and places of social aggregation. Participants will then provide two additional sputa for additional mycobacterial culture and WGS analysis. One sputum specimen will be stored as raw sputum (for direct DNA extraction), while the other will be decontaminated and used to inoculated MGIT medium as described above. Genomic *Mtb* DNA will be extracted from the MGIT culture, decontaminated sputum remnants or directly from the raw sputum specimen, (following depletion of human and other bacterial DNA) using the hexadecyltrimethylammonium bromide (CTAB) method. WGS analysis will be performed using an Illumina Next-Generation sequencing platform, facilitated by the NIH/NIAID TB Portals Program. If successful, WGS of the DNA extracted directly from sputum will provide analysis of the *Mtb* population structure without culture bias. Altogether, WGS data will be used to identify transmission of *Mtb* among PWUDs.

For Aim 3 Arm 1, 50 PWUD with active, untreated pulmonary TB will be recruited from Aim 2, and if necessary, the rest will be recruited from local health clinics. After providing informed consent, participants will receive a chest radiograph and undergo bioaerosol sampling. In Aim 3 Arm 2, study staff will recruit patients recently diagnosed with active TB disease who initiated treatment less than five days prior, or who are scheduled for treatment initiation from medical records at local health clinics, if they have provided permission to be contacted for this study. Study materials will also be placed within clinics and hospitals in the Worcester area so that individuals can contact study staff directly. Once individuals are screened for eligibility, informed consent is obtained and participants are enrolled, participants will then complete all study procedures that Arm 1 participants completed in Aim 2 and also undergo the Aim 3 procedures previously described.

### Exhaled bioaerosol collection

For Aim 3, exhaled bioaerosols will be collected with a modified Coriolis®μ Air Sampler, developed to quantitate the infectiousness of expired air [36]. This Respiratory Air Sampling Chamber (RASC) is used to collect and concentrate biological airborne particles (0.5–20 μm) using a high-flow wet walled cyclone, as previously described [37], with some modifications which include reducing the sampling period to two consecutive 15-minute sessions and the removal of continuous CO_2_, aerodynamic particle, temperature, and humidity monitoring. Droplet digital polymerase chain reaction (ddPCR) analysis will be used to detect *Mtb* within the exhaled air [38].

### RNA sequencing

RNA will be extracted from PAXgene tubes collected during Aim 2 or Aim 3 Arm 2 study procedures, and complimentary or cDNA prepared using an automated mRNA extraction, which would be done with a Hamilton EasyBlood Star at Stellenbosch University and cDNA synthesis procedure together with the PAXgene Blood RNA Kit (Qiagen, Germany). We will use the Fluidigm microfluidic qRT-PCR platform to evaluate the expression of genes selected from different published TB transcriptomic biosignatures, using TaqMan primer-probes. Assays will be run on either the 96.96 or 192.24 Gene Expression chips (Fluidigm) on a Biomark HD multiplex microfluidic qRT-PCR instrument. Analysis of gene expression data, including PCR controls and assessment of internal quality control samples to monitor performance of assays across individual Gene Expression chips, and computation of signature scores will be done with pre-prepared R scripts [39–41]. We will look for prevalence of the 16-gene host RNA signature previously studied by other research institutes in Worcester [32, 42], as well as other available signatures shown to be associated with higher risk of disease progression.

### Substance use and biobehavioural measures

TOTAL uses validated self-report questionnaires to assess patterns of substance use (Table 1). The Alcohol, Smoking and Other Substance Involvement Screening Tool (ASSIST) [43], Timeline Follow Back (TLFB) technique for drug use [44], and a modified version of The Important People Instrument [45], which assesses substance use within the participant’s social network, will capture substance use behaviour including frequency, quantity, patterns, and types of substances used. Participants who are identified as high risk for problems due to their substance use according to the ASSIST will be offered a referral to local treatment services.

**Table 1:** Biobehavioral assessments at study visit time periods.

	Assessment Measures										
	Biological Measures and Specimen Collection				Behavioural assessment	Psychosocial Measures			Biological assessment		RDS
Aim 1	Rapid Testing		Sputum for Xpert Ultra, Mtb Culture		Substance Use Measures	Mental health measures	MOSS-S (Medical Outcomes Social Support Survey)	Household Hunger Survey	TB symptom screen	Medical History (including HIV risk behaviour)	*Network Size
	*urinalysis for meth/Mandrax										*RDS rejection recruitment questionnaire
					*ASSIST (Alcohol, Smoking, Substance Use Involvement Screening Tool)	*CESD					*important people survey
	*blood for rapid HIV test		Blood for Host RNA Signature Measurement and COVID-19 Serology			*K-10					
					* Timeline Follow back (Meth/Mandrax)						
Aim 2			Sputum collection for Mtb culture and WGS (whole genome sequencing)								*social contact survey
											*important people survey
Aim 3a	Rapid Testing	Chest radiograph		Bioaerosol assessment *Respiratory Air Sampling Chamber (RASC)							
	*urinalysis for meth/Mandrax										
	*pregnancy test										
Aim 3b	Rapid Testing	Chest radiograph	Sputum collection for Mtb culture and WGS (whole genome sequencing), Blood for Host RNA Signature Measurement and COVID-19 Serology	Bioaerosol assessment *Respiratory Air Sampling Chamber (RASC)	Substance Use Measures	Mental health measures	MOSS-S (Medical Outcomes Social Support Survey)	Household Hunger Survey	TB symptom screen	Medical History (including HIV risk behaviour)	*Social contact survey
	*urinalysis for meth/Mandrax										
					*ASSIST (Alcohol, Smoking, Substance Use Involvement Screening Tool)^a^	*CESD					
	*pregnancy test					*K-10)					

Depressive symptoms will be screened for using The Center for Epidemiologic Studies Depression Scale (CES-D) [46], while the K-10 Kessler Psychological Distress Scale will be used to assess general psychological distress [47]. The Medical Outcomes Survey’s Social Support Scale [48], HIV risk-taking behaviour scale [49] and the Household Hunger Scale will also be used. Validity of these scales has been demonstrated in South Africa which have been widely used in populations of PLHIV/TB who use alcohol or other drugs [50–52]. Referrals will also be made if there are any concerns about individual’s mental well-being.

We will use the World Health Organization (WHO) four-symptom screening (current cough, a cough lasting ≥2 weeks, weight loss, night sweats, or fever) to evaluate for clinical versus subclinical TB [53].

### Data analysis

#### Sample size assumptions

For Aim 1, our estimated sample size of 750 individuals using RDS was calculated based on feasibility. Previous work suggests PWUD compared to non-users have a 2–6 fold increase in TB prevalence, translating into potentially detecting 30–90 TB cases from Aim 1 [12]. TB prevalence among the general population is estimated to range between 1–4% [32]. We assumed prevalence of drug use at 2.6% in the general population [54] and an estimated design effect (DE) of 4 allowing us to detect a three-fold increase in prevalence to 7.2% for PWUD with 80% power; if DE = 2 we can detect a two-fold increase to 5.7%. We therefore concluded that this sample size was appropriate under a variety of reasonable assumptions. Our sample size continues to provide 80% power if prevalence increases to 3%. The incipient disease signature prevalence in the community has been estimated at 13%, meaning that we have 80% power to detect a 1.4 (DE = 2) or 1.5 (DE = 4) fold increase in incipient disease among PWUD.

For Aim 2, other studies have consistently estimated that individuals who are in close contact with one another are linked by WGS in more than 70% of the sample [55]. If this is the case among PWUD, then for a sample size of 50 individuals, our precision will be 24.6% meaning our 95% confidence interval (CI) will range from 56.2% to 80.9%.

Patterson et al. found that 43% of individuals who had tested positive for drug-sensitive pulmonary TB by with Xpert Ultra had positive cough aerosols and those with non-zero colony forming units (CFU) measuring bacterial load had a median of 2.5 CFU [38]. Assuming that this is similar in controls, this would mean that by enrolling of 50 PWUD and 50 controls for Aim 3, we have 80% power to detect a 70% positive CFU probability in PWUD. Among those positive, we have 80% power to detect a 2.5-fold increase in the median CFU value with Mann-Whitney test and assuming CFU values are exponentially distributed. Similarly, assuming exhalation pattern frequency of 23.9 in controls, we have 80% power to detect a 2-fold increase in median exhalation frequency between PWUD and controls to enable us to demonstrate good power for the hypothesized drug use effect.

### Assessment of primary objectives

For the primary analysis, TB prevalence among PWUD will be estimated according to the Volz-Heckathorn estimator [56]. Analysis will be performed using the RDS package within R (r-project.org) and sensitivity analysis will be implemented using the SS estimator. Prevalence of TB among PLHIV and those without HIV will be estimated and compared to community survey estimates [57].

For the second aim, we will estimate the prevalence of clustered cases via WGS in our population of PWUD and check additional thresholds to confirm robustness. A combination of software packages has previously been assembled with optimised parameters, together with in-house developed scripts, to create a customised pipeline for the analysis of mycobacterial genomes, with specific focus on variant calling. This customised pipeline has been automated for *Mtb* WGS data analysis [58]. An in-house developed script is used to identify the variants, and high confidence single nucleotide polymorphisms are then used in further analyses. Phylogenetic software will be used including RaxML, IQ-TREE and MEGA, and phylogenomic trees will be visualised using FigTree software [59–61]. For clustering analysis, the single nucleotide variants used for phylogenomic inference will be used to determine the variant distances between isolates and cluster analysis will be performed with the R-programming language, implementing the APE (Analyses of Phylogenetics and Evolution) and ADEGENET (Exploratory Analysis of Genetic and Genomic Data) packages.

During statistical analysis we will employ a novel approach, using a Bayesian classification tool, to estimate a more nuanced probability of transmission between TB cases linked by drug use [62]. A subset of the data will be randomly selected to be the test set and links determined by a combination of WGS and social contacts are used as a gold standard. The Bayesian classifier is trained on this dataset to determine the relative contribution of other potential predictors of transmission, such as time between disease diagnosis/onset, gender, geographic distance and smear grade, and will be applied to the validation dataset to estimate the probability of a link between the remaining individuals in the dataset. This is done repeatedly, randomly selecting 1000 different training sets and estimating probabilities for each potential transmission pair to allow for the potential of an infector outside of the observed data network. We will also infer the relative contribution of attributes, such as HIV, to transmission probabilities. Simulations that we have performed with this approach show the area under the curve (AUC) of 0.85 compared to 0.65 with random covariates and excellent ability to determine the true infector.

For the third aim, we will investigate differences in *Mtb* transmission between participants with and without illicit drug use. Data generated from the ddPCR reaction will be analysed via the umbrella pipeline [63] to measure the difference between PUWD and those participants who do not use illegal drugs. Linear regression models will also be run, with the main predictor in this model will be an indicator of methamphetamine/Mandrax use. We will control for potential confounders of bacterial load, HIV status, radiographic findings, and smoking other substances for this model. A generalized estimating equation (GEE) approach will be used to account for matching on age and gender.

### Project status

Formative work was completed in February 2020, recruitment for the main study is ongoing (began April 2021 and is expected to end in August 2023).

## Discussion

This study will examine whether individuals with TB disease who smoke illicit drugs, particularly methamphetamine and Mandrax, are more likely to be infected by TB, progress to disease, and transmit *Mtb* compared to individuals with TB disease who do not smoke illicit drugs.

The main expected challenges in working with this target population is the timing of enrollment, attrition and a potentially lower number of TB cases than anticipated. Due to the fact that PWUD are a hidden population, there is a risk of not obtaining our sample size within the specified period due to challenges in engaging the target population. We will closely monitor enrollment patterns, and consequently implement strategies such as working closely with the community to generate interest around the study and extending the recruitment period until we are able to obtain our sample. The use of RDS should circumvent this challenge to a degree. Alternatively, interest may be greater, and enrollment faster than we anticipate, leading to challenges in managing participant flow. If enrollment is more rapid, we may hire additional staff. The risk of participant attrition is another challenge, but this will be limited by incorporating recruitment strategies that have been successful with previous studies that focused on PWUD, such as collecting detailed locator information and actively tracking participants between appointments, as well as the provision of reminders and reimbursement [64, 65]. COVID-19 also presents challenges in conducting study activities with South Africa having experienced a lengthy lockdown period in 2020 during which research activities were halted.

Through TOTAL’s approach of using RDS to estimate TB risk and disease prevalence in a sample of PWUD, we will utilise a well-established method for recruiting hard-to-reach populations in HIV research and apply this to examine TB and TB/HIV estimations. We will learn how to efficiently reach these and other high-risk networks for TB testing and linkage to care for future studies.

We will use novel approaches to increase understanding of TB disease in a potentially key population. This includes the use of host RNA signatures [39] to predict elevated risk of TB disease progression. We will leverage cutting-edge, Bayesian classification methods that use *Mtb* DNA WGS to create recent transmission probability scores. This approach will allow us to estimate the degree to which PWUD transmit TB to others and how frequently their infections reflect exposure in the network. We will also compare *Mtb* sequences isolated directly rather than cultured samples to maximize capture of *Mtb* genetic diversity. We will demonstrate that direct from specimen sequencing without enrichment is feasible [66] and assess whether additional linkages are captured between transmitter-contacts.

This cross-sectional investigation of the association between illicit drug use and TB transmission may reveal opportunities for innovative new interventions based on physiologic and behavioural factors. TOTAL’s study findings may inform new strategies for addressing the complex relationship between drug use, TB and HIV, such as investigating the roles of prompt identification and treatment of subclinical TB, increased HIV treatment, and increased awareness of drug use locations as sites of TB transmission.

## Supporting information

S1 Checklist Completed STROBE checklist for protocol. (PDF)

S1 File TOTAL protocol. This document provides the full protocol for the TOTAL Study as of May 2021.
(PDF)

S2 File (DOCX)

## Abbreviations

CFU: colony forming units
COVID: Coronavirus disease
DE: design effects
DNA: deoxyribonucleic acid
HIV: human immunodeficiency virus
MGIT: Mycobacteria Growth Indicator Tube
Mtb: mycobacterium tuberculosis
PCR: Polymerase Chain Reaction
PWUD: people who use drugs
RASC: respiratory aerosol sampling chamber
RDS: respondent driven sampling
RNA: ribonucleic acid
SUD: substance use disorder
TB: tuberculosis
WGS: whole genome sequencing
