# Supplementary material for: Transmission Of Tuberculosis Among illicit drug use Linkages (TOTAL): A cross-sectional observational study protocol using respondent driven sampling
Source: PLoS One. 2022 Feb 15;17(2):e0262440. doi: 10.1371/journal.pone.0262440 (PMC8846525; doi:10.1371/journal.pone.0262440)
Supplement: S1 File — This document provides the full protocol for the TOTAL Study as of May 2021. (PDF) [file pone.0262440.s002.pdf]

THE TRANSMISSION OF TUBERCULOSIS AMONG ILLICIT DRUG USE LINKAGES (TOTAL) STUDY SITE MANUAL OF PROCEDURES (STUDY SITE MOP)

V3.0 19 May 2021

UNITED STATES PI: DR. KAREN JACOBSON
SOUTH AFRICAN PI: PROF ROBIN WARREN

How to Use this Manual **5**
1 Introduction to the Study **5**
1.1 Study Sites 5
1.2 Study Team Organogram/Reporting Structure 6
2 Study Team Certification and Training Requirements **6**
3 Biometric Fingerprinting **7**
4 Eligibility Criteria **7**
5 Recruitment **7**
5.1 Initial Seed Selection 7
5.2 Aim 1 7
5.3 Aim 2 8
5.4 Aim 3 Arm 1 8
5.5 Aim 3 Arm 2 8
6 Visit Procedures **9**
6.1 Visit Procedures Overview Schematic 9
6.1.1 Initial Seeds 9
6.1.2 Aim 1 Visit 1 10
6.1.3 Aim 1 Visit 2 13
6.1.4 TB Results Visit 16
6.1.5 Aim 2 Visit 17
6.1.6 Aim 3 Arm 1 Visit 19
6.1.7 Aim 3 Arm 2 Visit 21
6.1.8 Participant Tracking for Visits 24
6.1.9 Suggested Visit Flow through Study Clinic 24
7 Appointments: Scheduling and Confirming **28**
7.1 Aim 1 Visit 1 28
7.2 Aim 1 Visit 2 28
7.3 Aim 2 Visit 28
7.4 Aim 3 Arm 1 Visit 28
7.5 Aim 3 Arm 2 Visit 29
8 Participant Contact Information **29**
8.1 Aim 1 Visit 1 and Aim 3 Arm 2 29
8.2 All Other Visits 30

9 Future TB Diagnoses 30
10 Blood Samples and Tests 30
10.1 Blood Draw Time Point and Procedures 30
10.1.1 HIV Testing 30
10.1.2 Serology 31
10.1.3 RNA (PAXgene® Tube) 34
10.1.4 Universal Precautions for Handling Blood Specimens 36
10.1.5 Venipuncture Procedure 37
11 Sputum Samples and Tests 37
11.1 Sputum Sampling Time Points and Procedures 37
11.2 GeneXpert Ultra *MTB*/RIF Testing 38
11.3Sputum Samples for *Mtb* Culture and Whole Genome Sequencing 41
11.4 Exhaled Bioaerosols Collection 45
11.4.1 Exhaled Bioaerosols Collection Procedure 46
11.4.2 Exhaled Bioaerosols Collection Procedure Dedicated Staff Training 46
11.4.3 Receipt, Handling, and Storage of Exhaled Bioaerosols 47
11.4.4 Short Term Storage of Collected Samples 47
11.4.5 Transport Instructions 47
11.4.6 Results 47
11.5 Urine Sampling 47
12 Chest X-Ray 49
13 TOTAL Lab Addresses and Contact Personnel for Shipping/Transport 50
14 Participant Visits for Collecting Missed Samples and Sample Collection Window's. 50
15 TOTAL Questionnaires 51
15.1 Aim 1 Visit 1 52
15.2 Aim 1 Visit 2 55
15.3 Aim 2 Visit 55
15.4 Aim 3 Arm 1 Visit 56
15.5 Aim 3 Arm 2 Visit 56
16 Siblings, Biological Relatives and Housemates (Aim 1 Visit 1) 56
17 Medical Record Data Abstraction 56
17.1 Medical Record Request Process 57
17.2 Handling Medical Records 57
18 Study Withdrawal Reasons 58

19 58

Study Site, Staff, and Participant Safety and Security Procedures 58

20 Adverse Events (AE) 59

20.1 Adverse Event Definition and Collection 59

20.1.1 Documentation and Review Process (AE Form) 59

20.1.2 Relatedness to Study Participation 60

20.1.3 Severity Grade Assignment 60

20.1.4 Serious Adverse Events (SAE) (SAE Form) 61

21 Protocol Deviations 63

21.1 Reportable Incidents: What Are Incidents? 64

22 TB Drug Resistance Incidental Findings 65

23 Consenting Guidelines 66

23.1Documentation of informed consent 67

23.2Additional considerations for illiterate participants 67

23.3Confidentiality 68

24 TOTAL Visit Scheduler Calendar 68

25 Guidelines for Conducting Participant Interviews 70

26 Infection Control for Staff and Participants 70

26.1 Risk classification of participants 71

26.2 General infection control measures 71

26.3 Physical cohorting 72

26.4 Safety protocol during specific procedures 73

26.5 Screening of Study Staff for Illness 73

27 Participant Transport 73

APPENDICES 73

Appendix I: Teaching Participants about Peer Recruitment 73

Appendix II: Linkage to TB Care 75

Appendix III: Reactive HIV Test Results: Counselling and Linkage to Care 75

Appendix IV: Referrals for Substance Use 77

Appendix V: Procedures for Identifying Distressed Participants and What to Do 78

Appendix VI: Utilizing the REDCap Database 85

How to Use this Manual

This manual outlines all **study implementation procedures** to be followed by the TOTAL field team. This manual is deemed final and ready for use by the field team upon approval by the PIs, conversion into a PDF, and stored on the field team Dropbox. The investigators may amend the manual. The manual version will increase each time it undergoes amending. The field team will receive an email memo indicating the manual has been amended, which sections were affected, and that the new version is available in the Dropbox. In addition to the electronic version, up to date paper versions in 3-ring binders must be kept at the TOTAL Office (Ukwanda) and TOTAL Study Clinic. To prevent waste, when amendments to the MOP are made, print and replace only the amended sections of the MOP, rather than the entire document. Maintenance of an up to date **electronic** MOP is the responsibility of the BMC Project Manager. Maintenance of an up to date **paper** MOP is the responsibility of the site Field Coordinator, and is subject to review at the quarterly audit.

IMPORTANT: THIS MANUAL FOCUSES ON STUDY IMPLEMENTATION FOR THE SUCCESSFUL DAY-TO-DAY OF STUDY ACTIVITIES. IT IS 1 OF 4 TOTAL STUDY MANUALS. THIS MANUAL IS ACCOMPANIED BY AN HR MANUAL, SAMPLE COLLECTION HANDBOOK, AND DATA MANAGEMENT/CLINICAL QUALITY MANANAGEMENT PLAN.

This manual is also accompanied by a number of SOPs that provide more detail about certain procedures such as infection control, waste management, sample packing and transport etc. These SOPs can be found in Dropbox-> Field Site SOPs

1 Introduction to the Study

TOTAL is a collaborative United States National Institutes of Health (NIH) funded research project. The overall objective of this study is to perform an investigation of People Who Use Drugs (PWUD) to identify the Tuberculosis (TB) disease burden in these groups, evaluate what proportion of these TB cases derive from recent transmission, and define mechanisms responsible for efficient transmission in this population.

There are 3 primary aims in TOTAL

Aim 1. To assess the TB exposure, risk of disease progression, and disease burden among people who use drugs (PWUD), specifically meth/Mandrax, with and without HIV.

Aim 2. To estimate the proportion of active TB cases resulting from recent transmission within the network identified in Aim 1.

Aim 3. Evaluate whether PWUD who have TB disease have physiologic characteristics associated with more efficient TB transmission compared to persons with TB disease who do not smoke drugs, with and without HIV.

1.1 Study Sites

The TOTAL Study Clinic

The TOTAL Study Clinic is the clinical site of the study, located in Worcester, Western Cape Province, South Africa. All recruitment, patient interviews, and clinical procedures (except for the RASC bioaerosol collection) will be conducted at this site. Sputum, blood, urine, and bioaerosol samples will be stored in a refrigerator at 2-4°C temporarily at this site in between transports.

Ukwanda Rural Clinical School, Stellenbosch University (Ukwanda)
The field team will have offices based at Ukwanda. This is also where all paper participant records and study documents will be securely stored. Laptops, tablets, and cell phones must also be securely stored at Ukwanda each night.

Worcester Radiology, Worcester
Worcester Radiology, located in Worcester, Western Cape Province, South Africa, is where chest x-rays (CXR) on study participants will be performed. A staff member will accompany participants to Worcester Radiology for this procedure, either by car or by foot.

Stellenbosch University (SUN)
Prof Robin Warren and his designated Post-Doctoral Fellow (Junior Scientist) are responsible for training and overseeing study staff, recruiting, enrolling, and retaining study participants, collecting and maintaining study data, overseeing sample collection, and retaining and storing all data. Archived electronic and paper participant records will also be stored securely at the SUN site.

Under the leadership of Dr. Robin Warren, SUN is also responsible for training and supervising staff in all sputum-based analyses (including culture, MIC, and *Mtb* DNA extraction), retaining and storing all sputum specimens, maintaining results in a secure database, and contributing to scientific output.

University of Cape Town (UCT) / Desmond Tutu Health Foundation
Under the leadership of Prof Robin Wood, UCT is responsible for training the field staff to conduct the RASC bioaerosol collections, developing related bioaerosol study documents, conducting bioaerosol analyses, and contributing to scientific output.

South African Medical Research Council (SAMRC)
Under the leadership of Drs. Tara Carney and Bronwyn Myers, SAMRC is responsible for running and analysing the focus groups to inform study design, developing the behavioural assessments, training the team on stigma in research, analysing RDS and behavioural data, and contributing to scientific output.

1.2 Study Team Organogram/Reporting Structure

Descriptions of the study team, reporting structure, and funding organization can be found in the **HR Manual.**

2 Study Team Certification and Training Requirements

For the TOTAL study, the primary South African Site, Stellenbosch University, will collect and track certifications. Certification requirements are dependent on an individual’s role in the study and/or the procedures he/she will perform.

The study field coordinator will be in charge of collecting, storing and tracking regulatory documents, including CVs, Good Clinical Practice (GCP) Certificates, TOTAL Training Certificates, and Delegation of Responsibility Logs (i.e., Delegation Logs).

Details on staff on-boarding and required trainings and certifications can be found in the **HR Manual.**

3 Biometric Fingerprinting

At each study visit participants will undergo biometric fingerprinting by the TOTAL study team upon arrival to the study clinic. Procedures for conducting the biometric fingerprinting can be found in dropbox, ***SOP: Registering and Managing a Participant Status on Biometric System.***

4 Eligibility Criteria

Up to date eligibility criteria can be found in the currently approved TOTAL Protocol. Eligibility criteria varies by study aim. Eligibility criteria is assessed at each aim through pre-screening and final screening, which are described in **Section: 6 Visit Procedures**.

5 Recruitment

5.1 Initial Seed Selection

Candidates for Aim 1 will be recruited via respondent driven sampling (RDS). RDS is a recruitment method often used to recruit hard to reach groups including people who use drugs. Via RDS, respondents recruit their peers, as in network-based samples, and researchers keep track of who recruited whom and their numbers of social contacts.

There will be 4-8 initial seeds selected for the study by the investigative team. Seeds are non-randomly selected members of the target population who initiate the RDS recruitment process. From each seed, a recruitment chain is expected to grow. Seeds play an extremely important role in conducting an RDS study. Seeds are selected based on their ability to recruit others into the study. A good seed should have a large social network. Seeds should be well connected to members of the peer group of interest. The investigative team will choose 4-8 seeds who they know use Tik and/or Mandrax and are well-networked from the TOTAL focus groups or other research done in the study area.

5.2 Aim 1

Aim 1 participants are recruited through RDS. Recruited participants will receive **2** recruitment coupons (see **Coupon** in Dropbox) to give to peers with whom they use tik and/or mandrax. Aim 1 participants, who have completed their own TOTAL visit, will then explain the process to their peers, who then decide for themselves whether or not they wish to participate. If they are interested, the study participant will give the peer the recruitment coupon, and instruct them to come to the study clinic to enroll in the study. It is critical that the study team provides adequate teaching to participants about who would be a "good" peer to approach *(See* ***Appendix I**: Teaching Participants about Peer Recruitment* *for more details and the **TOTAL Peer Recruitment Script** in Dropbox)*. Briefly, the peer should be someone who they use tik and/or mandrax with and is 15 years of age or older. The TOTAL team must also instruct the participant to keep their recruitment coupon safe, and when they hand the

coupon out to their peer, that they instruct the peer to keep the coupon safe. **Peers cannot enroll without a valid recruitment coupon**. The TOTAL team must make sure the participant is aware that they will only be reimbursed for **successfully recruited peers**, meaning the peer came to the study clinic, was found to be eligible for the study, and enrolled in the study. This again underlines the importance of properly instructing participants on who they can and should recruit.

5.3 Aim 2

Participants from Aim 1 who are found to have TB may be eligible for Aim 2 of the study. All participants who are found to have TB must be asked if they are interested in Aim 2, and if interested, consented and screened for Aim 2. The current eligibility criteria for Aim 2 can be found in the TOTAL Protocol. Eligibility criteria is assessed through Aim 2 screening, which is described in **Section 6: Visit Procedures****.**

5.4 Aim 3 Arm 1

Participants from Aim 1 who are found to have TB may also be eligible for Aim 3 Arm 1 of the study. All participants who are found to have TB must be asked if they are interested in Aim 3 Arm 1, and if interested, consented and screened for Aim 3 Arm 1. The eligibility criteria for Aim 3 can be found in the TOTAL Protocol. Eligibility criteria is assessed through Aim 3 screening, which is described in **Section 6: Visit Procedures****.** Participants who participate in Aim 3 **must also have participated** in Aim 2. Participants who participate in Aim 2 have the option to opt-out of Aim 3 *(consent for Aim 2 and 3 are on the same consent form, with the option to opt-out of Aim 3).*

5.5 Aim 3 Arm 2

Participants in the control arm of the study, Aim 3 Arm 2, will be recruited from local clinics in Worcester. We will use several methods to recruit these participants:

- Active recruitment

  We will identify participants via confirmation in their medical record of a positive TB result (evidence of MTB growth on Xpert Ultra, smear, or culture). We will approach the participant as they wait for their clinic appointment and let them know we are recruiting for a study, briefly describe the study, and let them know they may be eligible. If they are interested in learning more, we will re-approach them after their clinic visit and bring them to the RASC site in the back of the Worcester CDC for consent.

- Passive Recruitment

  We will hang flyers in the clinics and hospitals in the Worcester area which will have our study phone number on it. The flyer will indicate that if you were diagnosed with TB, to call the study phone number the day you were diagnosed to learn about a research study that you might be eligible for. On the phone we will ask a brief pre-screener. If the participant passes the pre-screener we will schedule to meet them at the Worcester CDC for consent. *(see the **Clinic Poster** in Dropbox for the IRB approved template)**.***

6 Visit Procedures

All visit procedures for each visit of the study discussed below are also listed in the **Study Visit Checklists** (*i.e Aim 1 Visit 1 Checklist, Aim 1 Visit 2 Checklist, Aim 2 Checklist, etc.)* to facilitate study staff in conducting study visits successfully.

6.1 Visit Procedures Overview Schematic

Figure 1: TOTAL Visit Procedures Schematic

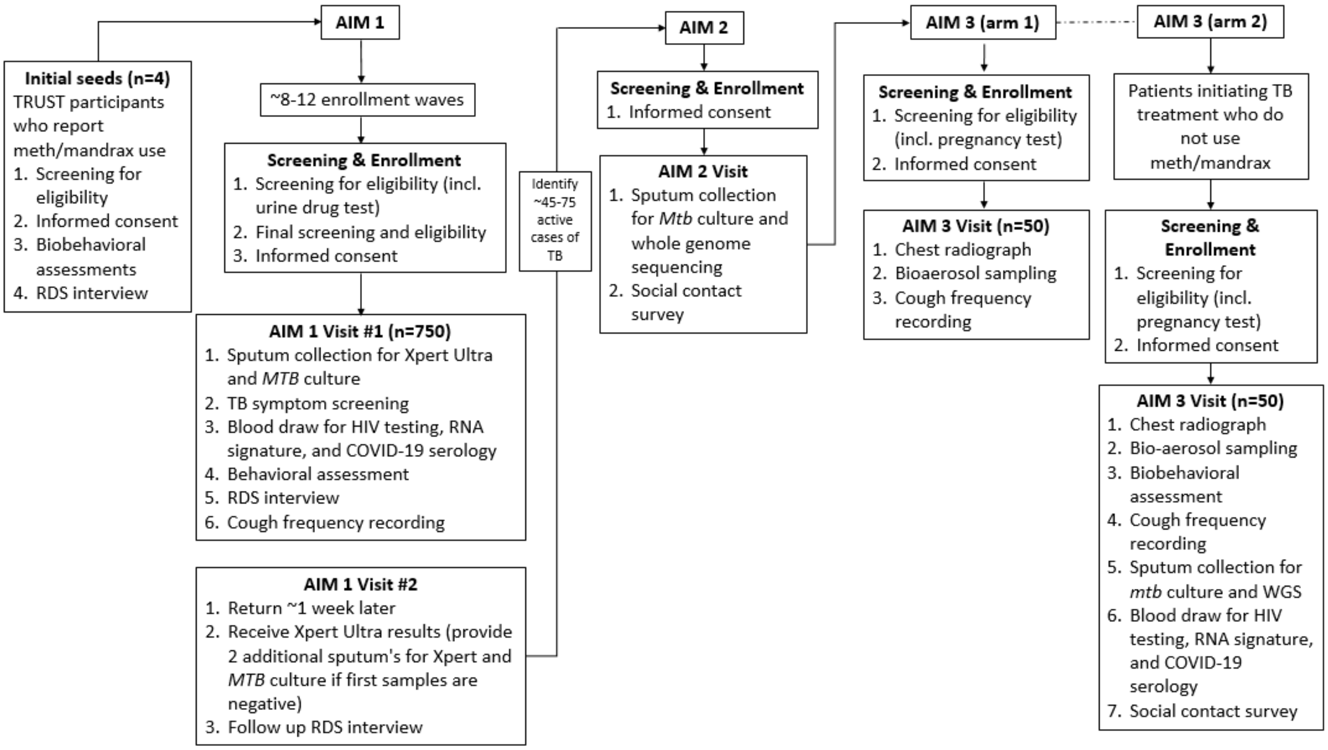

6.1.1 Initial Seeds

The investigative team will select initial seeds. A “good” seed in RDS recruitment is a person who fits the eligibility criteria and has a strong presence within the target network. TOTAL will recruit seeds identified from the TOTAL focus groups or the TRUST study. The initial seed will undergo the procedures outlined below for all other study participants. If the initial seed is identified through Aim 1 to have TB, they will follow the same procedures as other participants, and will be eligible for Aims 2 and 3 of the study.

The investigative team may choose to add additional seeds if initial recruitment chains end early or specific population characteristic(s) are underrepresented in the cohort. The Boston Based Project Manager will work with the investigative team using RDSAT software to determine if, and when reseeding should be done, and the required characteristics of the added seeds. Information on how the

group will use RDSAT software for Determining Equilibrium and Re-Seeding can be found in the **TOTAL Data Management/CQMP.**

6.1.2 Aim 1 Visit 1

Pre-Screening

Thorough screening of potential participants is critical for RDS. For a participant to be eligible to consent they **must** meet the following (confirmed via the **Aim 1 Pre-Screening Log**):

- Have a valid recruitment coupon
- Have not previously been enrolled in the study (*confirmed with biometric fingerprint, See* ***SOP: Registering and Managing a Participant Status on Biometric System***),
- Be 15 years or older

Every participant who arrives with a valid recruitment coupon should be added to the **Aim 1 Pre-Screening and Coupon Tracking Log** and assigned a **Screen ID**, regardless of if they pass the rest of the pre-screening and final screening. This is critical for linking the participant to their recruiter.

Consent and Screening

Prior to confirming eligibility for the rest of the study activities (drug screening), the participant needs to be consented.

- If the participant passes pre-screening and is interested in enrolling, the next step is to consent the participant in the language of their choice (Afrikaans, isiXhosa, or English). Follow the guidelines in Section 23: **Consenting Guidelines****.** This section also includes details on parental consenting and assent of minors (participants ages 15-17).
- Once the participant is consented, they must be added to the biometric fingerprint system (*See* ***SOP: Registering and Managing a Participant Status on Biometric System****)*
- Once the participant is consented, they need to pass the final screening for participation. This involves completing the **Aim 1 Screening Form** (REDcap) and providing urine for a urine drug test. Participants must have a positive urine drug test for either **methaqualone (mandrax) and/or methamphetamine (tik)** to be eligible to continue in the study (*for full list of inclusion and exclusion criteria see the current* ***TOTAL Protocol***).
- If a participant continues to be eligible after final screening (**Aim 1 Screening Form**), move on to the Aim 1 Visit 1 procedures.

  All participants who consent to the study will be assigned a unique Participant ID (PID). This PID needs to be added to the **Aim 1 Pre-Screening and Coupon Tracking Log** as it serves as the link between PIDs and identifying information. Even if a participant fails drug screening **they will still have a PID, as they were consented**.

Procedures

The first procedure to be conducted at Aim 1 Visit is sputum induction for sputum collection. This is so that if the participant cannot produce enough sputum in the first attempt, or requires a break, the rest of the sputum can be collected in a second attempt, later in the visit.

The following **biological tests** need to be completed during Aim 1 Visit 1 (in addition to the urine drug test which occurs at screening):

- Sputum Induction (2 samples) – Sputum is collected via sputum induction. Two samples will be collected, one to be sent for GeneXpert Ultra for *MTB*/RIF and cycle threshold, and one to be sent to Prof Warren's lab for smear and *Mtb* culture.
- Blood draw for RNA Signature (PAXgene) – PAXgene is collected for evaluation of the host RNA signature for incipient TB infection.
- Blood draw for HIV testing - All participants, including those who report known HIV infection, will undergo rapid testing (for diagnosis or to confirm their status). All results will be confirmed with a second test. Discordant results will be confirmed with a 3rd confirmatory test.
- Blood draw for COVID-19 serology – We will collect blood from all participants for serum aliquoting for future SARS-CoV-2 serology.

NOTE: All blood should be drawn from 1 needle stick

- *TBD: Cough frequency recording- participants will be audio recorded for one hour of their visit, during the questionnaires, to assess the frequency and phenotype of their cough.*

The following **questionnaires** need to be completed at Aim 1 Visit 1 (in addition to those completed during pre-screening and final screening).

NOTE: Surveys should be administered in the language of choice (Afrikaans, isiXhosa, English) of the participant, by a team member fluent in that language. Paper versions of the surveys in the various languages are available to be read aloud to the participant, but responses must be recorded on REDcap (which is in English).

- Important People Instrument
- The Alcohol, Smoking and Other Substance Involvement Screening Tool (ASSIST)
- RDS Network Size
- Timeline Follow Back (TLFB)
- TB Symptom Screen
- Social Support Survey
- Household Hunger Scale
- Center for Epidemiologic Studies Depression Scale (CES-D)
- Demographic Survey
- Medical History Survey
- K10 For Anxiety
- HIV Risk Survey
- Gang Association Survey

The following **logs and forms** (found on Dropbox) need to be completed during the Aim 1 Visit

- Coupon Sign Out Log- This log tracks which team member handed out recruitment coupons to participants, the date the participant was given their 2 recruitment coupons, the coupon numbers, and who the coupons were given to (PID)
- Aim 1 Pre-Screening and Coupon Tracking Log- This log is a critical component of RDS. This log indicates the coupon number of the coupon the participant received and brought in to be eligible for recruitment. It also indicates the coupon numbers of the 2 coupons the participant receives to go out and recruit their peers if they were enrolled (the coupons signed out in the Coupon Sign Out Log). This log tracks who recruits who in the network, as well as tracks who has come to the site with a valid coupon and was successfully enrolled, and who has picked up their reimbursement for successfully recruited peers. This log contains Protected Health Information (PHI) and therefore cannot be stored on Dropbox. This log is stored on the Microsoft Teams, is password protected, and can only be accessed via encrypted study laptops.
- ICF Checklist – this checklist is completed during consent to indicate all components of informed consent have been completed and the consent form signed according to GCP.
- ICF Log- This log is completed after the study visit to indicate which version of the consent form the participant signed (to monitor tracking of necessary re-consenting if required later), and to indicate if the participant consented to storage of samples and future contact.
- Coupon Receipt- The coupon receipt is a hard copy form that includes the coupon numbers of the coupons given to the participant for peer recruitment. This receipt is stored with the participant's hard copy study documents.
- Participant Visit Tracking Form- This log tracks which visit participants have finished, and if they started any visits but need to return to visit procedures, indicates that the participant needs to finish a visit. Located on REDcap
- Aim 1 Visit 1 Checklist- this checklist needs to be completed as the participant completes each component of their visit, by the RA or RN responsible for that part of the visit.
- Participant Clinic Form- The nurse/RA must fill out this form with the participant present. The participant reports any clinic they were diagnosed/treated at for TB/HIV/or COVID-19 in the past or presently. This form also records where participants were referred to, if they were referred for further TB, HIV, or substance use care. This form is critical as it will be used when requesting medical records for medical history verification. The data from this form is transcribed onto the Participant Clinic Log once per week.
- SATVI Form - This form is to be filled out for all participants who report having ever been in a SATVI STUDY. We use this information to collect information about the participant's time in that SATVI study, and what medication they may have received. This data in this form must be added to the SATVI Log once per week.
- PAXgene Procedures Data Collection Form – this form is on REDCap and tracks PAXgene sample collection and is available by the Walzl lab to record the time and date of the future RNA extraction. The field team completes the first half of the form while the Walzl lab completes the second half during RNA extraction.
- Blood Serology Data Collection Form- This form is on REDCap and tracks blood sample collection for serology. The field team completes the form when collecting whole blood, and when processing blood for serum aliquoting.
- Sputum Specimen Collection Data Collection Form- this form is on REDCap and tracks how, for which purpose, and when sputum was collected and specimen IDs.
- Urine Collection Form- This form is on REDcap and tracks whether urine was transferred to and stored in a 50 mL vial.

- Sample Tracking System – All samples collected (with the exception of blood for HIV testing, which is not stored) need to be added to the Sample Tracking System. Each sample will be assigned a unique barcode. The sample tracking system allows us to easily track where a participant's sample is at any given time (Study Clinic, in transit, Stellenbosch, etc.)
- Reimbursement Log- the reimbursement log needs to be filled out any time a participant receives reimbursement, including the baseline visit reimbursement.
- Reimbursement Receipt- Each time a participant receives a reimbursement the participant and the staff member assigning the reimbursement must sign two paper receipts, one for the participant and one for the study records.
- Locator Form- This form is critical for participant tracking if we need to finish components of the study or contact the participant in the future. Each time a participant attends a study visit their Locator Form must be updated to ensure the information is up to date.

Additional data collection forms are filled out after the Aim 1 Visit 1, upon receipt of sputum results. Results are filled out on the following REDcap forms:

- **Xpert Results DCF-** this form is filled out by the field team when Xpert results are obtained from NHLS.
- **Culture Results DCF**- this form is filled out by the SUN lab technologist and records information on culture growth, and if growth, DST/MIC testing results.

<u>Participant Reimbursement</u>

The participant must be reimbursed for their visit only once ALL surveys and procedures have been completed. This means if the participant's visit must span over several days, they are not reimbursed until the final day when they have completed everything.

6.1.3 Aim 1 Visit 2

Participants will receive their first round of sputum results at this visit, and if their results are negative, will provide additional sputum for a second round of TB testing. Participants will also be asked about who they have successfully and unsuccessfully tried to recruit and will be given their reimbursement for any successfully recruited peers.

<u>Participant Check-In</u>

Anytime a participant arrives at the clinic for a visit, they must be checked-in.

The Research Assistant must:

- Confirm if the participant has arrived for a scheduled visit or is a walk-in (check **TOTAL Visit Scheduler**)
- Confirm the participant's ID through biometric fingerprinting
- If the participant is a walk-in, confirm that the participant has not already had their Aim 1 Visit 2 on REDCap.

If GeneXpert or *Mtb* culture results are not available (check REDCap)

- Reschedule the participant to come back. GeneXpert results are typically ready within 1 to 2 weeks. Culture results are typically ready within 1 to 4 weeks. If it has not yet been 1 to 2 weeks, schedule the participant to come back after one to two weeks from their baseline visit. If it has been 1-2 weeks, the Xpert results should be in, if they are still not, you should check with the NHLS lab.

- Ask the participant if they have handed out any of their recruitment coupons

- If the participant says they have handed out their coupons, check the **Pre-Screening and Coupon Tracking Log.**

- If a coupon they handed out resulted in an enrollment, alert the Field Coordinator to complete the reimbursement (see Procedures section below for reimbursement and follow-up RDS questionnaire procedures)

If GeneXpert or *Mtb* culture results are available (check REDCap)

- The participant is eligible to have their Aim 1 Visit 2
- If the participant is here for a scheduled visit, have them sit in the waiting room to await their appointment.
- If the participant is a walk-in, confirm with the study clinic's daily (**TOTAL Visit Scheduler**) schedule that there is a timeslot available for an Aim 1 Visit 2 appointment. If there is availability, instruct the participant to wait in the waiting room for their nurse or RA.
- If there are no timeslots available for a full Aim 1 Visit 2, the participant must still be made aware of their TB results (if they are TB positive, check on REDcap).
- If there are no slots available, and the participant has negative TB results, you can reschedule them to return to the clinic at a later date. If the participant's TB results are positive, you must let a study nurse know so the nurse can give the participant their TB results and refer and link them to proper TB care.

Procedures

For participants who are GeneXpert Ultra and *Mtb* Culture *Negative* (on their first round of samples) (NOTE: you may only have Xpert Ultra results that are negative, while the culture is pending, this is OK, still continue to Aim 1 Visit 2 and collect two additional sputum samples)

The following **questionnaires** need to be completed:

- If a participant indicates they handed out coupons, the **Post-RDS Rejection Recruitment Questionnaire** must be completed,

The following **biological tests** need to be completed:

- Sputum Induction (2 samples) – Sputum is collected via sputum induction. Two additional samples will be collected, one to be sent for GeneXpert Ultra for *MTB*/RIF and cycle threshold, and one to be sent to Prof Warren's lab for smear and *Mtb* culture.

The following **logs and forms** need to be completed:

- Aim 1 Visit 2 Checklist- this checklist needs to be completed as the participant completes each component of their visit, by the RA or RN responsible for that part of the visit. This document needs to be reviewed for completeness and signed by the admin RA upon participant check-out.
- Sputum Specimen Collection Data Collection Form- This form is on REDCap and is filled out anytime sputum is collected to indicate sample type, date of collection, Specimen IDs, and how the sample was collected (induction or not).
- Sample Tracking System – All samples collected (with the exception of blood for HIV testing, which is not stored) need to be added to the Sample Tracking System. Each sample will be assigned a unique barcode. The sample tracking system allows us to easily track where a participant's sample is at any given time (Study Clinic, in transit, Stellenbosch, etc.)
- Reimbursement Log- the reimbursement log needs to be filled out any time a participant receives reimbursement, whether it is for a completed study visit or for successfully recruiting peers.
- Reimbursement Receipt- Each time a participant receives a reimbursement the participant and the staff member assigning the reimbursement must sign two paper receipts, one for the participant and one for the study records.
- Aim 1 Pre-Screening and Coupon Tracking Log- The coupon tracking log columns indicating reimbursement for participants who received reimbursement for successfully enrolled peers must be completed if the participant receives reimbursement at this visit for handing out coupons that resulted in successful enrollment.
- Locator Form- This form is critical for participant tracking if we need to finish components of the study or contact the participant in the future. Each time a participant attends a study visit their Locator Form must be updated to ensure the information is up to date.

Additional data collection forms are filled out after the Aim 1 Visit 2, upon receipt of the second round of sputum results. Results are filled out on the following REDcap forms:

- **Xpert Results DCF-** this form is filled out by the field team when Xpert results are obtained from NHLS.
- **Culture Results DCF**- this form is filled out by the SUN lab technologist and records information on culture growth, and if growth, DST/MIC testing results.

NOTE: These participants must be told that they will be contacted again to return to the study clinic if their second round of sputum results comes back positive.

For participants who are GeneXpert Ultra OR *Mtb* Culture *Positive*

Participants positive for TB are eligible for Aim 2 and possibly Aim 3 of the study. After the study nurse gives the participant their TB test results and conducts TB counselling, they should ask the participant if they are interested in participating in the other aims of the study. If the participant is interested, the

study nurse should consent the participant for the other aims of the study before continuing the visit, as they may be able to combine the Aim 1 Visit 2 and Aim 2 Visit, depending on the participant's schedule.

The following **questionnaires** for **Aim 1 Visit 2 only** need to be completed (see section on Aim 2 Visit and Aim 3 visits for their associated questionnaires and procedures, even if visits are combined):

- If a participant indicates they handed out coupons, the Post RDS Rejection Recruitment Questionnaire must be completed

The following **biological tests** should be completed:

- None. See section below for Aim 2 and 3 biological tests for participants who choose to enroll in subsequent aims.

The following **logs and forms** need to be completed:

- Aim 1 Visit 2 Checklist- this checklist needs to be completed as the participant completes each component of their visit, by the RA or RN responsible for that part of the visit. This document needs to be reviewed for completeness and signed by the admin RA upon participant check-out.
- Participant Visit Tracking Form- This log tracks which visit participants have finished, and if they started any visits but need to return to visit procedures, indicates that the participant needs to finish a visit. Located on REDcap
- Reimbursement Log- the reimbursement log needs to be filled out any time a participant receives reimbursement, including the baseline visit reimbursement.
- Reimbursement Receipt- Each time a participant receives a reimbursement the participant and the staff member assigning the reimbursement must sign two paper receipts, one for the participant and one for the study records.
- Aim 1 Pre-Screening and Coupon Tracking Log- The coupon tracking log columns indicating reimbursement for participants who received reimbursement for successfully enrolled peers must be completed if the participant receives reimbursement at this visit for handing out coupons that resulted in successful enrollment.
- Locator Form- This form is critical for participant tracking if we need to finish components of the study or contact the participant in the future. Each time a participant attends a study visit their Locator Form must be updated to ensure the information is up to date.

These participants must be linked to TB care *(See* ***Appendix II:* Linkage** ***to TB Care****).* These participants are also eligible for Aim 2 of the study, and possibly Aim 3. If the participant was eligible for Aim 2 of the study but did not have time to complete the visit at the same time as their Aim 1 Visit 2, they should be scheduled to return for their Aim 2 Visit, **as soon as possible.**

Participant Reimbursement

If a coupon the participant handed out resulted in an enrollment, the RA/RN must alert the Field Coordinator to complete the reimbursement and follow-up RDS questionnaire procedures.

6.1.4 TB Results Visit

If a participant has negative GeneXpert and sputum culture results when they return for their Aim 1 Visit 2, they will be asked to give two more samples (one for another Xpert and one for another culture). If the results of either of the additional testing are positive, the participant will need to be traced in the community and brought back into the study clinic for a TB Results Visit. This participant will have already completed their Aim 1 Visit 2 questionnaires, so at this visit will only need to be given their positive TB test results, and be linked to TB clinical care. These participants are eligible to enroll into Aim 2 (and possibly Aim 3) if they are interested. Complete the **TB Results Visit Checklist** during this visit.

6.1.5 Aim 2 Visit

Participant Check-In and Screening

Participants are eligible for Aim 2 if **any** of their up to 4 sputum samples (2 GeneXpert and 2 culture) from Aim 1 are positive for TB or they have a medical record confirmation of current active TB from less than 30 days ago. To enroll in Aim 2 participants must be consented. The consent form for Aim 2 and Aim 3 are combined, with the option for the participant to opt-out of participating in Aim 3 if they choose.

Every attempt should be made at Aim 1 Visit 2 to schedule a participant for their Aim 2 visit. There will be cases where a participant is negative at Aim 1 Visit 2, but their second round of samples comes back positive, and the participant will need to be traced in the community and asked to return for a TB results visit. At the TB results visit, participants who are interested in Aim 2 should be scheduled for an Aim 2 visit.

It is also allowed for the Aim 1 Visit 2 and Aim 2 visit to be combined (or TB results visit and Aim 2 Visit) pending the study clinic's staff capacity to do so and whether the participant agrees to hold the visit before being linked and transported to the clinic for further TB care. If a participant is scheduled for an Aim 2 visit and attend their visit, the RA checking in the participant must indicate their arrival on the visit scheduler, and instruct the participant to wait in the waiting room until they are called in for their visit.

If a participant is a walk-in for their Aim 2 Visit (missed their scheduled appointment and attends at a later time or date), you must first check that they are indeed eligible for Aim 2 (check REDCap that they have at least one positive TB test). Next, the study clinic schedule must be checked to see if there is a slot for an Aim 2 visit. If there is no availability the participant should be scheduled for another appointment date, as soon as possible.

Consent and Screening Survey

Eligibility criteria for Aim 2 includes indication of TB on any of the up to 4 sputum samples provided in Aim 1. (See Section **Inclusion and Exclusion Criteria of the TOTAL Protocol** for full list of criteria) or evidence of a positive TB result from a clinic.

- If the participant is eligible and is interested in enrolling, the next step is to consent the participant. Follow the guidelines in Section 23: **Consenting Guidelines**.
- The Screening Survey for Aim 2 must be filled out after consent, to indicate that this participant was eligible, and did or did not enroll in Aim 2 of the study.

Procedures

The following **questionnaires** need to be completed at the Aim 2 Visit (in addition to that completed during screening):

- Social Contact Survey

The following **biological tests** need to be completed during the Aim 2 Visit (in addition to the urine drug test which occurs at final screening):

- Sputum Induction (2 samples) – Sputum is collected via sputum induction. Two samples will be collected and transported to Prof Warren's Lab for interim storage, and shipped to TGen in the USA for analysis, one sample is for *Mtb* culture and WGS from the culture, and one for WGS from direct isolate.

The following **logs and forms** need to be completed during the Aim 2 Visit:

- ICF Checklist – this checklist is completed during consent to indicate all components of informed consent have been completed and the consent form signed according to GCP.
- ICF Log- This log is completed after the study visit to indicate which version of the consent form the participant signed (to monitor tracking of necessary re-consenting if required later), and to indicate if the participant also consented to Aim 3 of the study.
- Aim 2 Visit Checklist- this checklist needs to be completed as the participant completes each component of their visit, by the RA or RN responsible for that part of the visit. This document needs to be reviewed for completeness and signed by the admin RA upon participant check-out.
- Sputum Specimen Collection Data Collection Form- This form is on REDCap and is filled out anytime sputum is collected to indicate sample type, date of collection, Specimen IDs, and how the sample was collected (induction or not).
- Sample Tracking System – All samples collected (with the exception of blood for HIV testing, which is not stored) need to be added to the Sample Tracking System. Each sample will be assigned a unique barcode. The sample tracking system allows us to easily track where a participant's sample is at any given time (Study Clinic, in transit, Stellenbosch, etc.)
- Reimbursement Log- the reimbursement log needs to be filled out any time a participant receives reimbursement.
- Reimbursement Receipt- Each time a participant receives a reimbursement the participant and the staff member assigning the reimbursement must sign two paper receipts, one for the participant and one for the study records.
- Aim 1 Pre-Screening and Coupon Tracking Log- (if required, for tracking and reimbursement of successfully recruited peers)
- Locator Form- This form is critical for participant tracking if we need to finish components of the study or contact the participant in the future. Each time a participant attends a study visit their Locator Form must be updated to ensure the information is up to date.

Participant Reimbursement

The participant must be reimbursed for their visit only once ALL surveys and procedures have been completed. This means if the participant's visit must span over several days, they are not reimbursed until the final day when they have completed everything. The participant must also be reimbursed if a

coupon they handed out resulted in an enrollment. The RA/RN must alert the Field Coordinator to complete the reimbursement and follow-up RDS questionnaire procedures.

6.1.6 Aim 3 Arm 1 Visit

Participant Check-In and Screening

Participants are eligible for Aim 3 Arm 1 if **any** of their up to 4 sputum samples (2 GeneXpert and 2 culture) from Aim 1 are positive for TB, have started TB therapy less than 5 days ago, participated in Aim 2, and they are a non-pregnant woman. To enroll in Aim 3 Arm 1 participants must be consented. The consent form for Aim 2 and Aim 3 are combined, with the option for the participant to opt-out of participating in Aim 3 if they choose.

Every attempt should be made at the Aim 2 visit to schedule a participant for their Aim 3 visit. Once participants are given their positive TB test results and linked to care, they will likely repeat testing at the clinic and start treatment within a week. A participant should complete Aim 3 Arm 1 before they initiate TB therapy, and at the absolute latest a maximum of 5 days after treatment initiation. For this reason, every attempt should be made to schedule the participant for their Aim 3 visit, **as soon as is possible after their Aim 2 visit.**

It is also allowed for the Aim 2 and Aim 3 visit to be combined pending the study staff's capacity to do so. It's also possible that Aim 3 procedures are scheduled before Aim 2 procedures, such as the RASC, if

If a participant is scheduled for an Aim 3 visit and shows up for their scheduled visit, the RA checking in the participant must indicate their arrival on the online visit scheduler, and instruct the participant to wait in the waiting room until they are called in for their visit.

If a participant is a walk-in for their Aim 3 Visit (missed their scheduled appointment and attends at a later time or date), you must first check that they are indeed eligible for Aim 3 (check REDCap that they have at least one positive TB test) and that they consented to Aim 3 on the Aim 2 and 3 consent form. If they are a pre-menopausal woman they also must provide urine for a pregnancy test to confirm eligibility. If the participant continues to be eligible, the clinic schedule must be checked to see if there is enough staff to hold an Aim 3 Arm 1 visit. If there is no availability the participant should be scheduled for another appointment date, preferably for the next day.

Consent and Screening Survey

Eligibility criteria for Aim 3 includes indication for TB on any of the up to 4 sputum samples provided in Aim 1, initiated TB therapy < 5 days, participated in Aim 2, and negative urine pregnancy test for pre-menopausal women. (See Protocol for full list of Inclusion and Exclusion Criteria). The participant will be confirmed as eligible regarding TB through the Aim 2 screening, and eligible based on the pregnancy criteria after consent, through urine pregnancy testing.

- If the participant is eligible and is interested in enrolling, the next step is to consent the participant. Follow the guidelines in Section 23: **Consenting Guidelines****.**

- After consent, and upon check-in for the Aim 3 Arm 1 visit, pre-menopausal women must provide urine for pregnancy testing, to confirm continued eligibility.
- The Screening Survey for Aim 3 must be filled out to indicate that this participant was or was not eligible, and did or did not enroll in Aim 3 of the study.

Procedures

The following **questionnaires** need to be completed at the Aim 3 Arm 1 Visit (in addition to that completed during screening):

- No additional questionnaires need to be completed at the Aim 3 Arm 1 Visit, aside from the Screening Survey completed after consent.

The following **biological tests** need to be completed during Aim 3 Arm 1 (in addition to the urine drug test which occurs at final screening):

- Exhaled Bioaerosol collection in the Respiratory Aerosol Sampling Chamber (RASC) – Participants will sit in the RASC for one hour wearing a specialized suit, and encouraged to breathe normally for the duration of the sampling. This system collects exhaled Bioaerosols for *Mtb* analysis.
- Cough Frequency Testing – Cough frequency testing occurs in the RASC, at the same time as the bioaerosol collection. The participants' spontaneous coughs are recorded during the one-hour sampling period.
- Chest Radiograph – Participants will be transported to Worcester Radiology for a chest x-ray.

The following **logs and forms** need to be completed during the Aim 3 Visit:

- ICF Checklist – this checklist is completed during consent to indicate all components of informed consent have been completed and the consent form signed according to GCP. This may have been checked during the Aim 2 Visit if the participant consented to Aim 3 then.
- Aim 3 Arm 1 Visit Checklist- this checklist needs to be completed as the participant completes each component of their visit, by the RA or RN responsible for that part of the visit. This document needs to be reviewed for completeness and signed by the admin RA upon participant check-out.
- Participant Visit Tracking Form- This log tracks which visit participants have finished, and if they started any visits but need to return to visit procedures, indicates that the participant needs to finish a visit. Located on REDcap
- Sample Tracking System – All samples collected (with the exception of blood for HIV testing, which is not stored) need to be added to the Sample Tracking System. Each sample will be assigned a unique barcode. The sample tracking system allows us to easily track where a participant's sample is at any given time (Ukwanda, in transit, Stellenbosch, etc.)
- Reimbursement Log- the reimbursement log needs to be filled out any time a participant receives reimbursement.
- RASC Procedure Data Collection Form- This form is on REDCap and is filled out anytime the RASC procedure is conducted to indicate date of collection, Specimen ID etc.
- Chest Radiograph DCF- the field team fills out the first half of this form when a participant is sent for a chest radiograph. The study radiologist, to record her read of the radiograph, completes the second part.

- Reimbursement Receipt- Each time a participant receives a reimbursement the participant and the staff member assigning the reimbursement must sign two paper receipts, one for the participant and one for the study records.
- Aim 1 Pre-Screening and Coupon Tracking Log- (if required, for tracking and reimbursement of successfully recruited peers)
- Locator Form- This form is critical for participant tracking if we need to finish components of the study or contact the participant in the future. Each time a participant attends a study visit their Locator Form must be updated to ensure the information is up to date.

Participant Reimbursement

The participant must be reimbursed for their visit only once ALL surveys and procedures have been completed. This means if the participant's visit must span over several days, they are not reimbursed until the final day when they have completed everything. The participant must also be reimbursed if a coupon they handed out resulted in an enrollment. The RA/RN must alert the Field Coordinator to complete the reimbursement and follow-up RDS questionnaire procedures.

6.1.7 Aim 3 Arm 2 Visit

Aim 3 Arm 2 is the control arm of TOTAL. These participants are recruited external to the main cohort and have active TB disease and must **not** use any of the smoked substance we test for. These participants may be recruited from clinics and hospitals in Worcester or may be discovered through community contacts.

Pre-Screening

For a participant to be eligible to consent they **must** meet the following (confirmed via the Aim 3 Arm 2 Screening Survey):

- Have microbiologic confirmation of pulmonary Tuberculosis
- Started TB therapy <5 days ago
- Have never been enrolled in the study previously (pre-checked by first and last)
- Be 15 years or older

*(for full list of inclusion and exclusion criteria see the current **TOTAL Protocol**).*

Final screening for inclusion criteria (including drug screening (urine and self-report) and pregnancy testing (pregnant women will be excluded due to x-ray scheduled for this visit), will occur after consent.

Every participant who is pre-screened for enrollment must be added to the **Aim 3 Arm 2 Pre-Screening Log** and assigned a **Screen ID**, regardless of if they pass the rest of the pre-screening criteria. This helps us to track participants who have been screened previously and the reasons they were or were not eligible or did or did not participate.

Consent and Screening

Prior to confirming eligibility for the rest of the study activities (drug screening and pregnancy testing), the participant needs to be consented.

- If the participant passes pre-screening and is interested in enrolling, the next step is to consent the participant. Follow the guidelines in Section 23: Consenting Guidelines**.**
- Once the participant is consented they need to pass the final screening for participation. This involves confirming this is a new enrollment with biometric fingerprint, completing the **Aim 3 Screening Form**, and providing urine for a urine drug test and pregnancy test.
- Participant Visit Tracking Form- This log tracks which visit participants have finished, and if they started any visits but need to return to visit procedures, indicates that the participant needs to finish a visit. Located on REDcap
- If a participant continues to be eligible after screening, move on to the Aim 3 Arm Visit 2 procedures.

Only participants who consent to the study will be assigned a unique Participant ID (PID). This PID needs to be added to the pre-screening log as it serves as the link between PIDs and identifying information. Even if a participant does not pass final screening, they must have a unique PID, as they have been consented.

Procedures

Participants in Aim 3 Arm 2 receive nearly the same surveys as those enrolled in Aim 1 and Aim 2. The following **questionnaires** need to be completed at the Aim 3 Arm 2 visit (in addition to those completed during screening and pre-screening):

- Social Contact Survey
- The Alcohol, Smoking and Other Substance Involvement Screening Tool (ASSIST)
- TB Symptom Screen
- Social Support Survey
- Household Hunger Scale
- Center for Epidemiologic Studies Depression Scale (CES-D)
- Demographic Survey
- Medical History Survey
- K10 For Anxiety
- HIV Risk Survey
- Gang Association Survey

The following **biological tests** need to be completed during the Aim 3 Arm 2 visit (in addition to the urine pregnancy and drug test which occurs at final screening):

- Sputum Induction (2 samples) – Sputum is collected via sputum induction. Two samples will be collected, one for *Mtb* culture for WGS and one for WGS from direct isolate.
- Exhaled Bioaerosol collection in the Respiratory Aerosol Sampling Chamber (RASC) – Participants will sit in the RASC for one hour wearing a specialized suit, and encouraged to breathe normally for the duration of the sampling. This system collects exhaled Bioaerosols for *Mtb* analysis.
- Cough Frequency Testing – Cough frequency testing occurs in the RASC, at the same time as the bioaerosol collection. The participants' spontaneous coughs are recorded during the one-hour sampling period.
- Chest Radiograph – Participants will be transported to Worcester Radiology for a chest x-ray.

- Blood draw- for COVID-19 serology, RNA signature (PAXgene), and HIV testing if no medical record confirmed positive diagnosis.

The following **logs and forms** need to be completed during the Aim 3 Arm 2 visit (in addition to the Pre-Screening Log and Aim 3 Arm 2 Screening Form):

- ICF Checklist – this checklist is completed during consent to indicate all components of informed consent have been completed and the consent form signed according to GCP.
- ICF Log- This log is completed after the study visit to indicate which version of the consent form the participant signed (to monitor tracking of necessary re-consenting if required later), and to indicate if the participant consented to sample storage and future contact.
- Aim 3 Arm 2 Visit Checklist- this checklist needs to be completed as the participant completes each component of their visit, by the RA or RN responsible for that part of the visit. This document needs to be reviewed for completeness and signed by the admin RA upon participant check-out.
- Participant Visit Tracking Form- This log tracks which visit participants have finished, and if they started any visits but need to return to visit procedures, indicates that the participant needs to finish a visit. Located on REDcap
- Participant Clinic Form- The nurse/RA must fill out this log with the participant present. The participant reports any clinic they were diagnosed/treated at for TB/HIV/or COVID-19 in the past or presently. This form also records where participants were referred to, if they were referred for further TB or HIV care. This form is critical as it will be used when requesting medical records for medical history verification. This log must be transcribed once per week to the Participant Clinic Log.
- SATVI Form - This form is to be filled out for all participants who report having ever been in a SATVI STUDY. We use this information to collect information about the participant's time in that SATVI study, and what medication they may have received. This data in this form must be added to the SATVI Log once per week.
- Sputum Specimen Collection Data Collection Form- This form is on REDCap and is filled out anytime sputum is collected to indicate sample type, date of collection, Specimen IDs, and how the sample was collected (induction or not).
- PAXgene Procedures Data Collection Form – this form is on REDCap and tracks PAXgene sample collection and is available by the Walzl lab to record the time and date of the future RNA extraction. The field team completes the first half of the form while the Walzl lab completes the second half during RNA extraction.
- Blood Serology Data Collection Form- This form is on REDCap and tracks blood sample collection for serology. The field team completes the form when collecting whole blood, and when processing blood for serum aliquoting.
- RASC Procedure Data Collection Form- This form is on REDCap and is filled out anytime the RASC procedure is conducted to indicate date of collection, Specimen ID etc.
- Chest Radiograph DCF- the field team fills out the first half of this form when a participant is sent for a chest radiograph. The study radiologist, to record her read of the radiograph, completes the second part.
- Urine Collection DCF- this team is filled out to confirm whether urine was transferred to a 50 mL plastic vial for future analyses.
- Sample Tracking System – All samples collected (with the exception of blood for HIV testing, which is not stored) need to be added to the Sample Tracking System. Each sample

will be assigned a unique barcode. The sample tracking system allows us to easily track where a participant's sample is at any given time (Study Clinic, in transit, Stellenbosch, etc.)

- Reimbursement Log- the reimbursement log needs to be filled out any time a participant receives reimbursement, including the baseline visit reimbursement.
- Reimbursement Receipt- Each time a participant receives a reimbursement the participant and the staff member assigning the reimbursement must sign two paper receipts, one for the participant and one for the study records.
- Locator Form- This form is critical for participant tracking if we need to finish components of the study or contact the participant in the future. Each time a participant attends a study visit their Locator Form must be updated to ensure the information is up to date.

Participant Reimbursement

The participant must be reimbursed for their visit only once ALL surveys and procedures have been completed. This means if the participant's visit must span over several days, they are not reimbursed until the final day when they have completed everything.

6.1.8 Participant Tracking for Visits

Aim 1

Participants must be instructed at the Aim 1 Visit 1 to return in approximately 1-2 weeks for their Aim 1 Visit 2. Some participants may not return. If participants have not returned within 2 weeks, they should be tracked and reminded to return for their visit. Once a week the Field Coordinator must run the "Aim 1 Participants to Track" report on REDCap. Visit nurses who performed the Aim 1 Visit 1 should try to contact the participant by phone. If they are unavailable by phone, the logistics coordinator must track them in the community (using the information from the locator form).

Aim 2 and 3

Participants who have positive sputum on any of their Aim 1 samples (Aim 1 V1 or Aim 1 V2) or current TB confirmed by the medical record must be tracked and brought in to receive their results, and to see if they are willing to participate in Aim 2 (and 3). As soon as we know these participants have TB (from any of their sputum, or medical record confirmation of current TB) they should be contacted to bring them in for their results and recruitment into Aim 2 (and 3). Once a week the Field Coordinator must run the "Aim 2 Participants to Track" report on REDCap. Visit nurses who performed the either Aim 1 V1 or Aim 1 V2 should try to contact the participant by phone. If they are unavailable by phone, the logistics coordinator must track them in the community (using the information from the locator form).

6.1.9 Example Visit Flow through Study Clinic

Below are examples of how participants should flow through the study clinic for each visit type. For more details see the **Visit Checklists** (Dropbox), which describes the step-by-step procedures for all tasks that need to be done before, during, and after the visit.

Aim 1 Visit 1

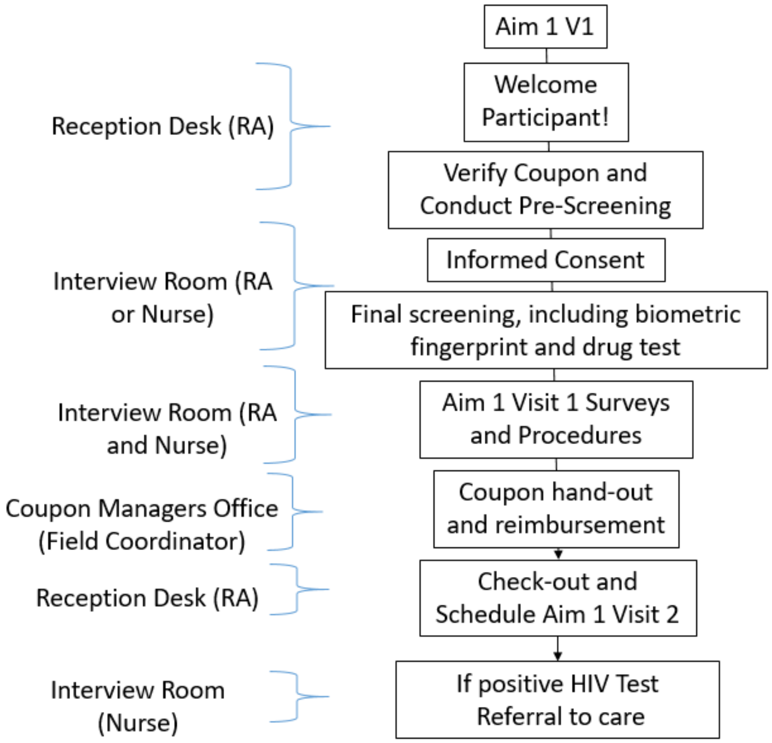

Aim 1 Visit 2

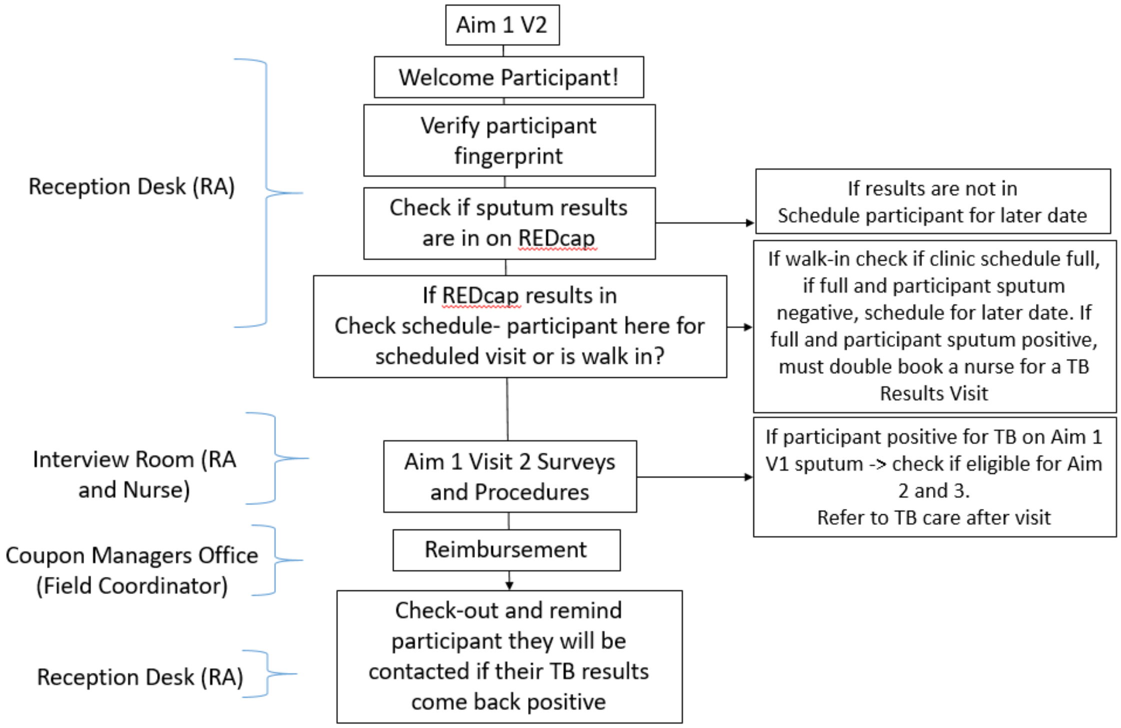

TB Results Visit

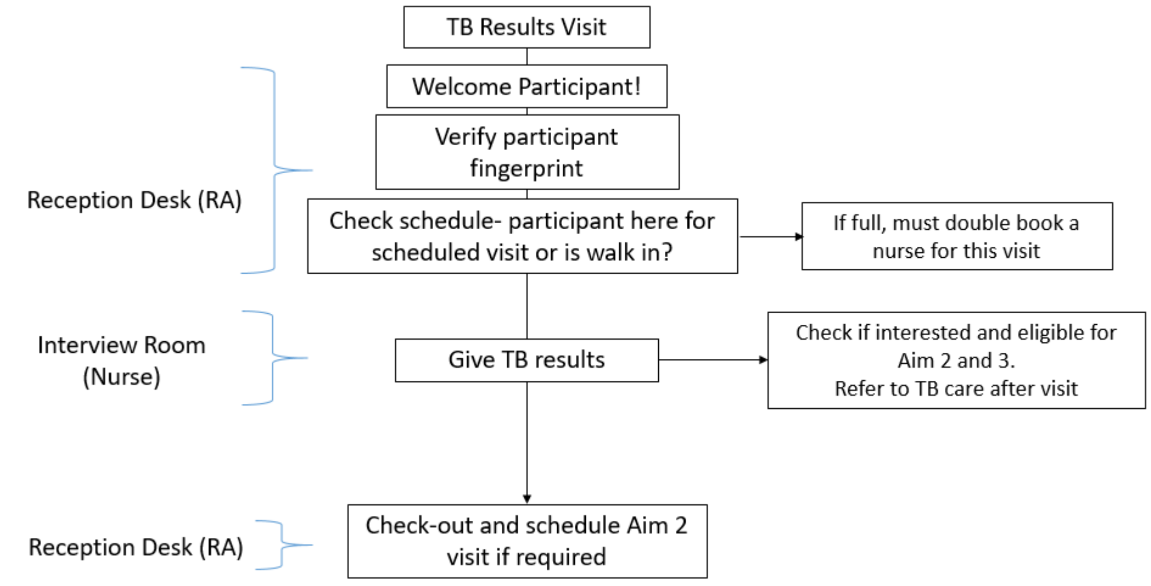

Aim 2 Visit

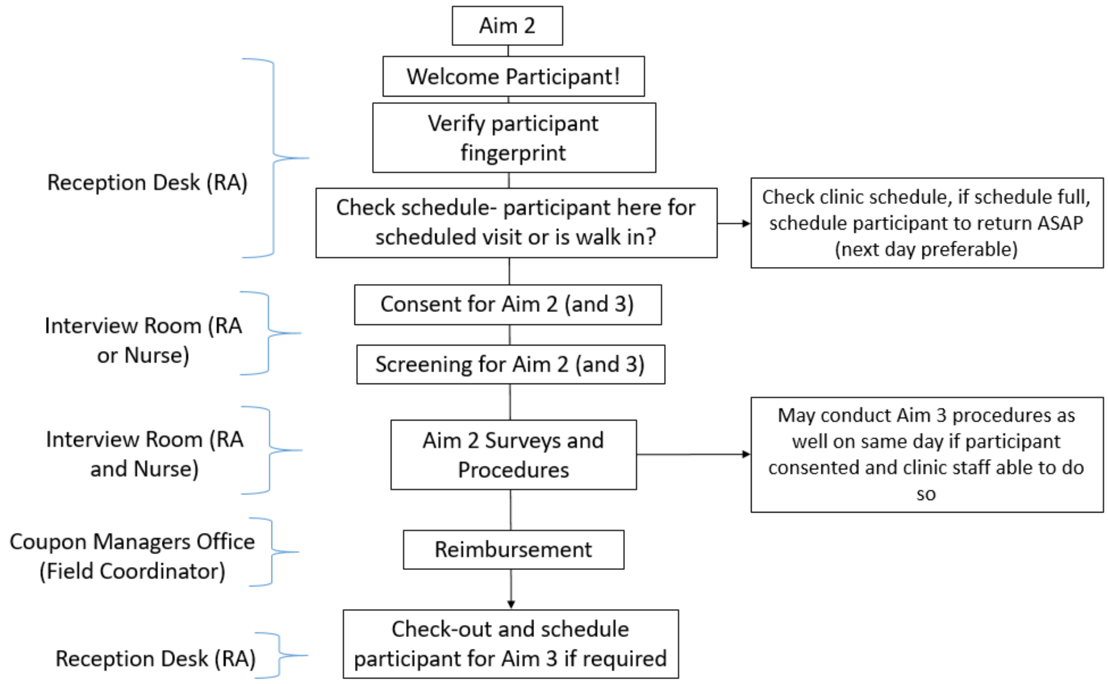

Aim 3 Arm 1 Visit

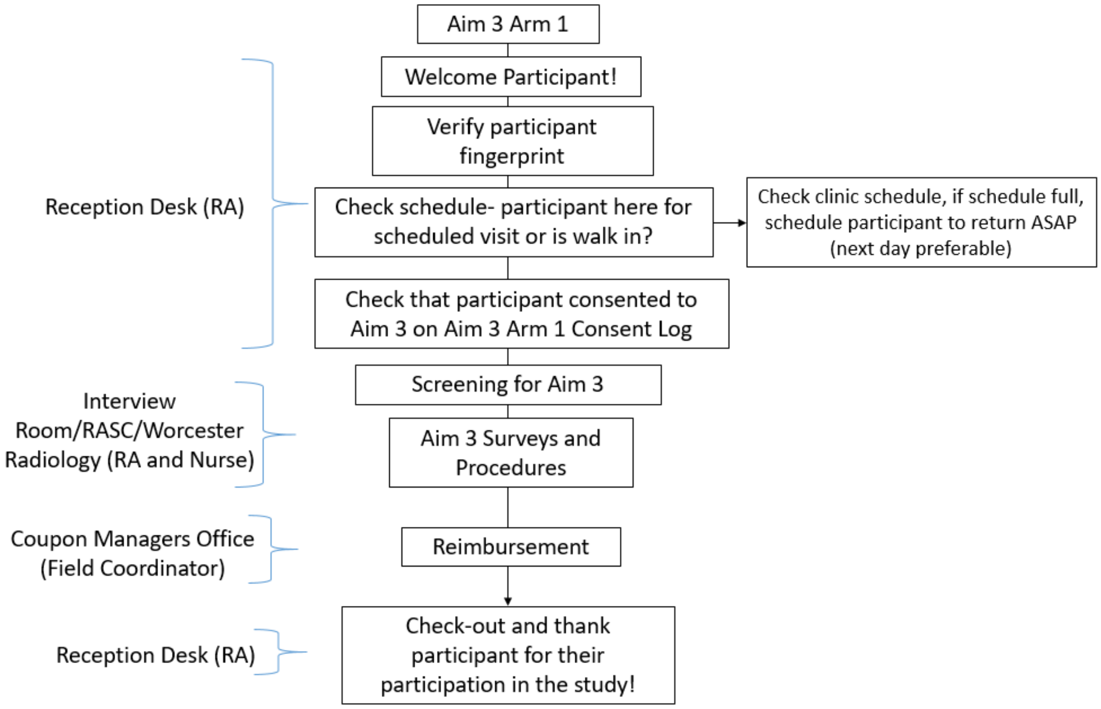

Aim 3 Arm 2 (Controls)

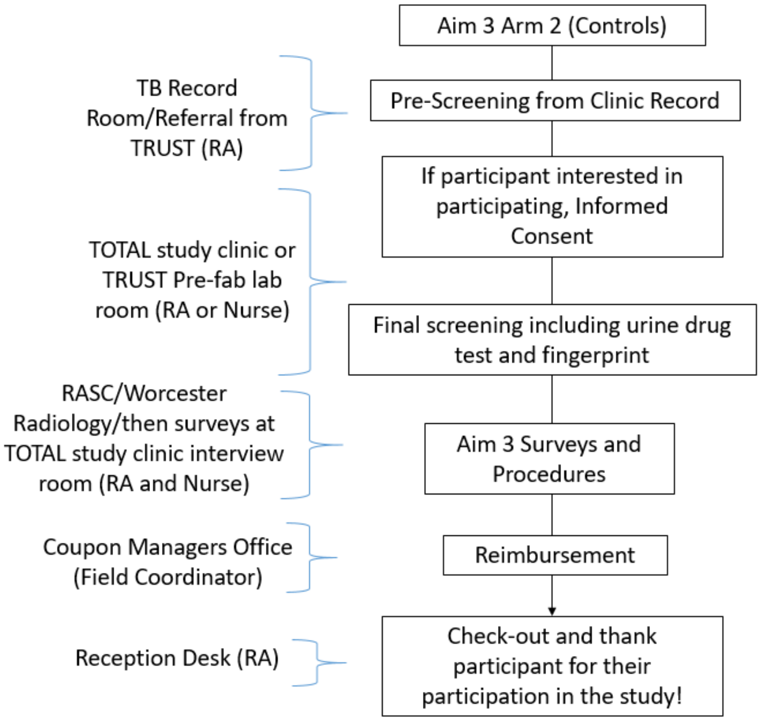

7 Appointments: Scheduling and Confirming

This section describes procedures the team must follow to track participants and for scheduling and confirming study visits.

7.1 Aim 1 Visit 1

In Aim 1 of TOTAL, candidates who receive a recruitment coupon from an enrolled peer or seed **(initial seeds are pre-identified by the study investigative team)**, are eligible to come to the study site to be screened and, if they meet the additional eligibility criteria, consented into the study. Prior to candidates coming to the study site and consenting, we **cannot** contact them for enrollment, even if we know who they are based on details from their recruiter. We **can**, however, encourage and support their recruiter to check-in with their peer about the study and ask if there are obstacles to them attending the visit. If the recruiter reports back that the candidate is willing to attend the study visit, but cannot make it due to a transportation barrier, we can arrange transport for the candidate.

7.2 Aim 1 Visit 2

It is critical that participants return to the study clinic for their Aim 1 Visit 2, at a minimum of one week after their baseline visit, and a maximum of two months from their baseline visit, to receive their TB testing results. Once the team sees that sputum results are available, every effort should be made to contact the participant and schedule them for their Aim 1 Visit 2. At Aim 1 Visit 1, it is critical to fill out the participant's **Locator Form**, with as much detail as possible. This would include their contact details: phone numbers where they can be contacted, home addresses, and other places where they can be contacted. At the participant's Aim 1 Visit 1, study staff should attempt to schedule the participant's Aim 1 Visit 2 in approximately 1-2 weeks. Write the scheduled date on the participants scheduling card and encourage them to keep the card safe, and enter the scheduled visit date into the study site's electronic scheduling calendar (the **TOTAL Visit Scheduler**). By consenting to the study, participants consent to be contacted about study appointments. We will utilize all contact information provided in the locator form to reach a participant to remind them of or schedule their Aim 1 Visit 2. Methods include phone call to the participant, phone call to all other numbers provided by the participant, and home visits (if permitted by the participant).

7.3 Aim 2 Visit

If a participant is found to be eligible for Aim 2 and chooses to enroll, every effort should be made to have the Aim 2 visit on the same day as their Aim 1 Visit 2. This will help to prevent loss to follow-up between Aim 1 and Aim 2. If a participant is interested in enrolling in Aim 2, but cannot stay for the visit that day, the participant should be scheduled for the Aim 2 visit as soon as possible, and given a participant scheduling card. The study staff should use every contact method to remind the participant of their scheduled visit. If the participant misses their scheduled visit, every attempt should be made to reach the participant to reschedule their Aim 2 Visit.

7.4 Aim 3 Arm 1 Visit

Participants can consent to Aim 3 during their Aim 1 Visit 2 while consenting to Aim 2 (both Aims are on the same consent form). If possible, the Aim 1 Visit 2, Aim 2 Visit, and Aim 3 Visit can be held on the same day. This scenario may often not be practical, as the Aim 3 visit includes the chest x-ray and

bioaerosol sampling, however; it is allowed. It is also permitted to conduct Aim 3 procedures before Aim 2, such as the RASC and X-Ray before the social contact survey, if necessary. Similarly, if the patient attends their Aim 1 Visit 2 and cannot stay for their Aim 2 visit, they can, and should be scheduled for a combined Aim 2 and Aim 3 visit as soon as possible (the next day preferred). The Aim 3 Visit can also be held individually. Please keep in mind participants must complete their Aim 3 Visit prior to beginning TB treatment or at a maximum of 5 days after starting treatment. Participants identified as possibly having TB through Aim 1 will be referred to their preferred clinic. The clinic will likely complete TB testing and the participant will likely return to the clinic within a week to receive their clinic TB test results and start treatment. This is a short time to get the participant into Aim 3, and thus all attempts should be made to schedule the Aim 1 Visit 2, Aim 2 Visit, and Aim 3 visit together, and at a minimum, to schedule the Aim 2 and Aim 3 visit together. As with the other aims, all contact methods should be used to reach the participant for visit scheduling and reminders.

7.5 Aim 3 Arm 2 Visit

Participants will be identified as eligible for Aim 3 Arm 2 (control arm, TB positive patients who do not use drugs) through one of the clinics or hospitals in Worcester. It is crucial that we approach participants as soon as possible about study enrollment. If a participant is interested they should be consented and complete the bioaerosol assessment on the day of diagnosis and a maximum of 5 days after treatment initiation. If a participant does not have time to go for a chest-x-ray and and/or complete the questionnaires, these can be scheduled for another day, within one week of TB treatment initiation. As with the other aims, all contact methods should be used to reach the participant.

8 Participant Contact Information

8.1 Aim 1 Visit 1 and Aim 3 Arm 2

A detailed and up to date **Locator Form** is critical for RDS and participant tracking in the community because it allows us to capture up to date and accurate information on how to find our participants for subsequent visits, or if we need to contact them later.

Its purpose is to collect pertinent participant identification information including full name, address, and telephone number in as much detail as possible. In the case that participants live in informal housing, where sometimes there are not clear street names, include directions using any landmarks when adding information to the locator form. When possible, alternate phone numbers should be secured. If a cell number is provided, ask the participant if he/she can accept text messages and document the participants preferred means of communication.

Information for alternative (emergency) contacts (such as other family members, close friends, or neighbors) is also collected. These individuals will be contacted only if study personnel are unable to reach the participant through all other means.

General Information

- The Locator Form serves as source documentation proving the existence of the participant. It **must** be completed.

- It is important to obtain complete and accurate addresses, phone numbers etc. for the participant during Aim 1 Visit 1. The participant will need to be contacted if they miss any scheduled visits, or if their second round of Aim 1 sputum sampling is TB positive, and they need to be traced in the community. Similarly, it is critical to collect this information for the control arm, Aim 3 Arm 2 participants, as participants may be unable to enroll and have the visit the day they are approached, and we will have to contact them to schedule their visit for the next day.

- There should be a hard copy locator form filled out for each participant consented into Aim 1 and Aim 3 Arm 2. Store the paper Locator Forms in the TOTAL Locator Form study binder (organized with dividers, by PID). This form contains the participant's name, address, and other identifying information and is therefore **confidential** and should ONLY be accessed by TOTAL research staff involved with recruitment and participant interaction.

8.2 All Other Visits

Anytime a participant attends the study clinic, the RA completing the visit must update the Locator Form. If there are no updates, please indicate "no updates" on the line.

9 Future TB Diagnoses

After Study Completion

It will be described to participants during consent that if the participant is diagnosed with TB or COVID-19 within 5 years of their final study visit, we will access information on their diagnosis, treatment plan, GeneXpert Ultra results, and culture and smear status. The study team will collect this information from NHLS, the medical record, and/or the Western Cape Provincial Health Data Centre.

10 Blood Samples and Tests

10.1 Blood Draw Time Point and Procedures

A blood draw is required at Aim 1 Visit 1 (cases) and Aim 3 Arm 2 Visit (controls). Blood is drawn in one tube for HIV testing, one tube for COVID-19 serology, and a third tube for PAXgene RNA.

10.1.1 HIV Testing

HIV Testing is conducted at Aim 1 Visit 1 and Aim 3 Arm 2 Visit (if participant does not have a document HIV test in the past one month). For the detailed procedure on how to conduct HIV testing see the **HIV Testing Section of the Sample Collection Handbook.**

<u>Overview</u>

Obtain blood sample for HIV testing (venipuncture)
Conduct pre-test counselling
Conduct HIV testing per procedures defined in **HIV Testing Section of the Sample Collection Handbook.**

Conduct post-test counselling
Fill out HIV questions on **Medical History Survey CRF**
Disposal of biohazardous waste
After the visit, linkage to care if required

Results

The results of the HIV test must be provided to the participant at the end of their study visit, along with HIV counselling for reactive test results, and linkage to clinical care *(See **Appendix III**: Reactive HIV Test Results: Counselling and Linkage to Care).*

Blood Sample Collection, Processing and Interim Storage

Blood Tube List

**Item**	**# Per Collection**
2 mL **Purple Top** EDTA blood collection tube (for venepuncture only)	1

Sample ID Labels
Blood tubes for HIV testing only need to be labelled with a participant ID label as they are disposed of immediately after HIV testing.

Procedures
See **HIV Testing Section of the Sample Collection Handbook** *for full supply list and detailed procedures.*

Sample, Test Kits, and Supply Disposal
All materials used during HIV testing, including: lancets, phlebotomy kits, blood tubes, gloves, HIV testing kits, etc. need to be disposed of in a biohazard waste container. Biohazard waste containers are considered "full" when the fill line on the container is reached, no further waste is to be added to the container at that time. Full containers must be scheduled for pick up by an external biohazardous waste company chosen for use by the TOTAL study.

10.1.2 Serology

Blood for serology is collected at Aim 1 Visit 1 and Aim 3 Arm 2 visit. For the detailed procedure on how to collect blood for serology see the **Blood Draw for Serology section of the Sample Collection Handbook.**

Overview
Obtain blood sample for serology (10 mL gold top SST tube)
Enter sample into Sample Tracking System
Complete Blood Serology DCF on REDcap
Process blood at study clinic (centrifuge)
Interim storage of separated whole blood

Transport separated whole blood to SUN
SUN aliquoting of serum from separated whole blood
Store aliquots at -80 at SUN.

Results

Blood serum aliquots will be transported to the Stellenbosch University, Tygerberg for storage and future analyses. No results will be provided to the study participants.

Sample, Test Kits, and Supply Disposal
All unused tubes will be stored at room temperature unless otherwise noted on the package label. Tubes should not be exposed to extreme temperatures, as this negatively impact the effectiveness of tubes and results. Stock will be rotated when tubes expire. Dispose of materials used during the procedure following the ***Procedures for Biohazards Disposal SOP.***

Blood Sample Collection, Processing and Interim Storage

Blood Tube List

**Item**	**# Per Collection**
10 mL **gold top** SST tube	1
1.5 mL serum aliquot vials	As many as needed for # serum aliquots collected (~3)

Sample Labels

**STS Sample Type**	**Sample ID**	**Sample Purpose**	**# Labels Needed Per Collection**
Whole blood for serology	TCOVID.__ __ __ __ __. __ __<br>P or CT (4 # PID) aliquot #	Whole blood collected	1
Serum	TSERO.__ __ __ __ __. __ __<br>P or CT (4 # PID) aliquot #	Serum aliquots for future serology	As many as needed for # serum aliquots collected (~3)

Note: The label must also include the date of the blood draw (dd/mm/yyyy).

Whole blood will be processed at the study clinic (via centrifuging), processed separated whole blood will be stored in the interim at 2-4C at the study clinic before transport transported to Stellenbosch University. Serum from separated whole blood will be aliquoted at SUN and will be frozen at -80 degrees until future analyses are conducted. See the **COVID-19 Serology section of the Sample Collection Handbook** for more details on whole blood processing (centrifuging and aliquoting) and interim serum storage at the study clinic.

Transport and Long-Term Storage
Separated whole blood in Gold Top SST Tubes should be transferred from interim storage at the study clinic to long-term storage at Stellenbosch University (Tygerberg) **within 3 days of the sample collection.** They can be couriered along with the regular weekly sputum specimens.

1. Separated whole blood should be packed in accordance with South African guidelines on infection control. They should be packed in a cooler box with enough cold packs sufficient to maintain a 2 – 8 ˚C temperature range during transport.
2. See ***SOP: Specimen Packing for Transport*** for more details on proper specimen packing prior to transport.
3. The cooler box with the vials should be put into the cold room of the MBHG department (same location that sputum specimens are left for transfer of custody). The designated Stellenbosch Lab Technologist will retrieve these samples within 24 hours.

Long-term storage of serum aliquots

Serum from the separated whole blood will be aliquoted at SUN. Serum aliquots will be stored long term at SUN in a -80 freezer until further analyses is conducted.

Laboratory procedures for serology

SUN will perform SARS-CoV-2 serology on serum pending future funding. Detailed procedures to be added.

Serology Results Data Upload

Transcriptomic data will be shared with the participating institutions via a method to be determined.

Preparing a shipment inventory for blood for Serology

To create a shipment (shipment in this context indicates samples for transport) in the database application, the field coordinator will run the **Serology Shipping Report** on REDCap which includes a listing of the barcodes in the shipment and corresponding participant ID. The team member responsible for preparing the shipment will check-out each Serology sample listed on the Shipping Report in the Sample Tracking System.

The team member must generate a Shipment Inventory Form (See Form: **Shipment Inventory Form** in Dropbox) for the serology samples that contains: details on type of samples (PAXgene, sputum etc.) date of shipment, transporter initials, and the Serology Shipping Report, and print and include these documents in the shipment container.

When site personnel prepare a shipment for transport, the Field Coordinator must notify the receiving lab via e-mail with a summary of the contents of the shipment, the expected time of arrival of the shipment, and an electronic version of the **Serology Shipping Report** listing the specimen IDs (and associated participant IDs) included with the shipment.

Confirmation of Received Samples

The lab technologist receiving the serology samples will enter “Sample Received” and the date received on the participant’s REDCap **Blood Serology Collection Form.**

Overview of Packaging Blood for Serology Sample Shipments

Blood for serology will be centrifuged prior to shipment, and must be handled carefully as to not mix the sample. Proper packaging is critical to ensure no mixing or accidental breakage. See ***SOP: Specimen Packing for Transport*** for detailed instructions on how to pack blood for serology for transport.

10.1.3 RNA (PAXgene® Tube)

PAXgene collection is conducted at Aim 1 Visit 1 and Aim 3 Arm 2 visit. For the detailed procedure on how to collect blood for PAXgene see the **PAXgene section of the Sample Collection Handbook.**

<u>Overview</u>
Obtain blood sample for RNA (one 10 mL labelled PAXgene® Blood RNA tube)
Enter sample into Sample Tracking System
Fill out PAXgene Collection DCF
Store sample in interim at study clinic
Transport sample for long-term storage at SUN Immunology Research Group (SU-IRG)

<u>Sample, Test Kits, and Supply Disposal</u>
Store the unused PAXgene Blood RNA Tubes at room temperature. Do not use past the expiration date. Dispose of all materials properly following the ***Procedures for Biohazards Disposal SOP.***

<u>Results</u>

PAXgene® tubes will be transported to the SU-IRG, Tygerberg, for processing and analyses. No results will be provided to the study participants.

<u>Blood Sample Collection, Processing and Interim Storage</u>

Blood Tube List

Item	# Per Collection
10 mL PAXgene® Blood RNA tube (CE)<br>BD #762165 (16x100 mm)	1

Sample Labels

STS Sample Type	Sample ID	Sample Purpose	# Labels Needed Per Collection
TOTAL_RNA	TRNA . __ __ __ __ __ .__ __<br>P or CT (4 # PID) RNA sample #	RNA Expression Studies	1

Sample labels must be pre-printed with TRNA and 7 underscores. E.g TRNA.__ __ __ __ __. __ __. PAXgene samples are labelled with the participants PID and the number PAXgene sample this is for the participant. Most, if not all participants will have only 1 PAXgene sample, however the option is open for labelling in case a second sample is requested by the investigators. For example, the barcode for the first PAXgene sample for Aim 1 participant (case) P0123 is TRNA.P0123.01. The second PAXgene sample (if requested by the PIs to take a second sample) for Aim 3 Arm 2 participant

(control) C0011 is TRNA.C0011.02. The label must also include the date of the blood draw (dd/mm/yyyy).

Interim storage of PAXgene tubes

1. Collected PAXgene tubes should always be stored upright
2. After collection, store the PAXgene tube at room temperature (i.e., 18-25C) for a minimum of two hours, but no more than 72 hours, before transferring to the 2-8 degree fridge
3. The PAXgene tube can be stored at 2-8 degree fridge for a **maximum of 5 days** before transport to long term storage at -20 or -80.

Transport and Long-Term Storage

PAXgene tubes should be transferred from interim storage at the study clinic to long-term storage at Stellenbosch University (Tygerberg) once per week. A maximum of 10 PAXgene blood tubes will be stored at any time in the study clinic refrigerator. They can be couriered along with the regular weekly sputum specimens.

1. The PAXgene tubes should be packed in accordance with South African guidelines on infection control. They should be packed in a cooler box with enough cold packs sufficient to maintain a 2 – 8 ˚C temperature range during transport. It is imperative that the tubes remain upright during transport
2. See ***SOP: Specimen Packing for Transport*** for more details on specimen packing for transport.
3. The samples are dropped of to the Immunology Lab (PI Gerhard Walzl) on the second floor in the BMRI, in a dedicated sample receipt area with 4 to 5 research assistants managing the space.
4. The samples may be dropped off (08:00 and 16:30) Monday to Friday.

Long-term storage of PAXgene tubes

1. PAXgene tubes should always be stored upright, and should be stored long-term in a wire rack or paper box. They should not be stored in Styrofoam trays, as this can cause the tubes to crack.
2. PAXgene tubes can be stored long-term (up to 11 years) at either -20 ˚C or -80 ˚C
3. If the PAXgene tubes are to be stored at -80 ˚C, they must be transferred into a -20 ˚C freezer for a minimum of 24 hours before going into a -80 ˚C freezer
4. Once stored, the Lab Technologist should update REDCap to indicate the receipt and storage location of each sample in the Sample Tracking System.

Laboratory procedures for PAXgene tube RNA analysis

Gerhard Walz's laboratory at Stellenbosch, Tygerberg will conduct mRNA extraction and RT-PCR experiments to capture host RNA signature and for preservation of purified RNA and cDNA. Detailed procedures to be added.

RNA Results Data Upload

Transcriptomic data will be shared with the participating institutions via a method to be determined.

Preparing a shipment inventory for PAXgene

To create a shipment (shipment in this context indicates samples for transport) in the database application, the field coordinator will run the **PAXgene Shipping Report** on REDCap which includes a listing of the barcodes in the shipment and corresponding participant ID. The team member responsible for preparing the shipment will check-out each PAXgene sample listed on the Shipping Report in the Sample Tracking System.

The team member must generate a Shipment Inventory Form (See Form: **Shipment Inventory Form** in Dropbox) for the PAXgene samples that contains: details on type of samples (PAXgene, sputum etc.) date of shipment, transporter initials, and the PAXgene Shipping Report, and print and include these documents in the shipment container.

When site personnel prepare a shipment for transport, the Field Coordinator must notify the receiving lab via e-mail with a summary of the contents of the shipment, the expected time of arrival of the shipment, and an electronic version of the PAXgene Shipping Report listing the specimen IDs (and associated participant IDs) included with the shipment.

Receiving Confirmation of Shipments

The Walzl Lab will email a log of all received samples. Using the log, the admin RA must navigate to the participants' PAXgene Collection Form on REDcap and mark "sample received" and the "date received" (this info, including date received, will be on the Walzl Lab Log).

Overview of Packaging PAXgene Sample Shipments

PAXgene tubes are glass and can break easily. Proper packaging is critical to ensure no accidental breakage. See ***SOP: Specimen Packing for Transport*** for detailed instructions on how to pack PAXgenes for transport.

10.1.4 Universal Precautions for Handling Blood Specimens

The TOTAL study recommends the following general laboratory safety protocol for the field site laboratories:

- Use non-permeable lab coats, latex (or nitrile) gloves, and face shields when handling any blood in any situation in which splashes, spray, splatter, or droplets of blood may be generated and eye, nose, or mouth contamination can be reasonable anticipated.
- Disposable latex gloves and lab coats are worn when collecting and processing specimens. Hands are washed thoroughly with disinfectant soap prior to handling samples and afterward prior to leaving the work area. Skin cuts or abrasions should be covered.
- Follow "Standard Precautions" when handling any blood, urine or tissue products. All specimens must be handled as potentially infectious for laboratory workers.
- Contaminated needles and sharps shall be immediately placed in a puncture-resistant, leak proof biohazard container. Never recap or break needles.
- Leftover biologic/medical waste should be immediately discarded in appropriate medical waste receptacles
- Bleach is used to clean up any spills of blood, plasma, or serum; and all laboratory work surfaces at the completion of work activities.

10.1.5 Venipuncture Procedure

See the **Venipuncture Procedures section of the Sample Collection Handbook** for details on the necessary supplies, how to properly draw blood, and guidelines for any blood draw complications.

11 Sputum Samples and Tests

11.1 Sputum Sampling Time Points and Procedures

Two sputum samples must be collected at Aim 1 Visit 1, one for *Mtb* culture and one for GeneXpert Ultra, both used for TB testing. If a participant is negative on both GeneXpert Ultra or *Mtb* culture, the tests are repeated at Aim 1 Visit 2 following the flowchart below.

Sputum is collected via sputum induction whenever possible. For each participant, the **Pre-Sputum Induction Participant Health Checklist** is completed to identify contraindications to sputum induction. In the case a participant has a contraindication to sputum induction, sputum will be collected through traditional deep cough collection. For more details *see* ***Sputum Induction Procedures section of the Sample Collection Handbook.***

Figure 2: (Flow Chart for Sputum Collection for Aim 1 and 2)

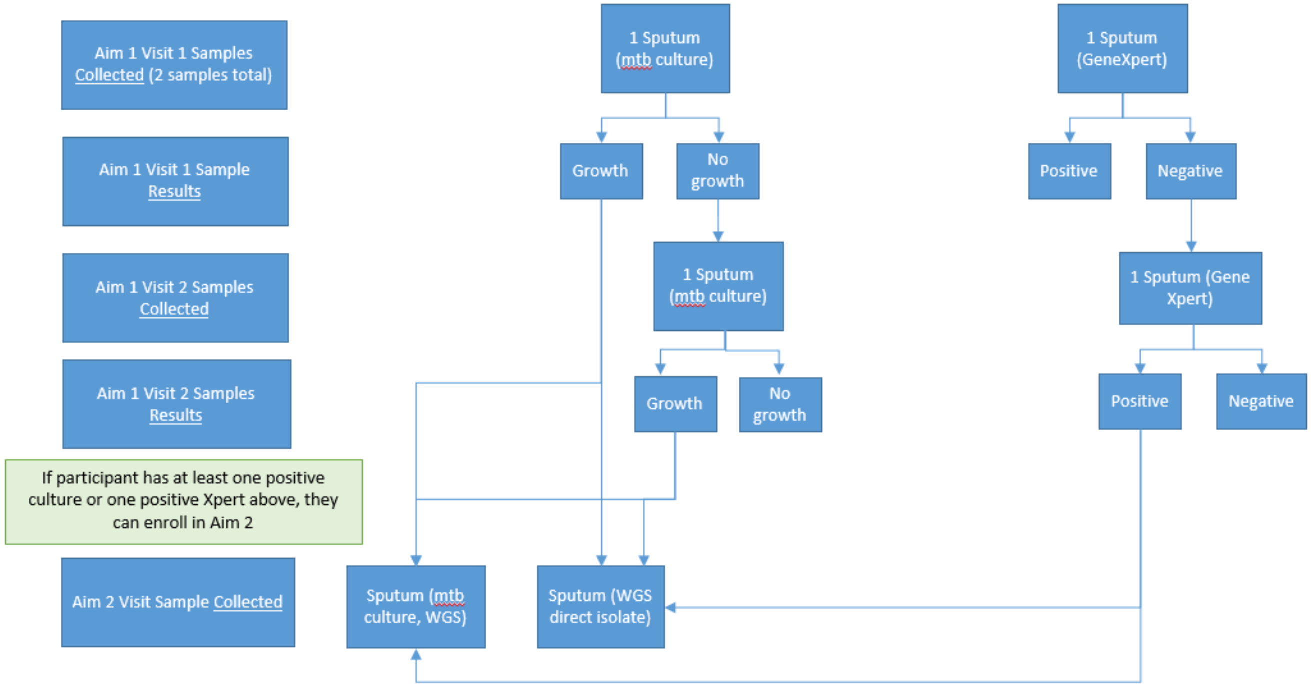

Participant's with either a positive GeneXpert or *Mtb* growth in culture on any of the 4 samples they provide in Aim 1, are eligible to enroll in Aim 2, and to provide sputum for whole genome sequencing (WGS) from culture and direct isolate (pending funding).

11.2 GeneXpert Ultra *MTB*/RIF Testing

The World Health Organization (WHO) recommended the use in all settings of a next-generation Xpert® MTB/RIF assay (called Xpert® MTB/RIF Ultra) as a replacement for the current Xpert MTB/RIF® cartridge as of March 2017.

The Ultra cartridge, that has been developed by Cepheid (Sunnyvale, USA), showed significantly better performance (increased sensitivity) compared to the current Xpert® MTB/RIF cartridge for the detection of *Mycobacterium tuberculosis* in specimens with low numbers of bacilli, especially in smear-negative, culture-positive specimens (such as those from persons with HIV co-infection), in paediatric specimens and in extra-pulmonary specimens (notably cerebrospinal fluid).

All participants enrolled in Aim 1 will provide sputum for GeneXpert Ultra MTB/RIF testing. Samples will be collected by the study team, but analysis will occur through the National Health Laboratory Services (NHLS) in Worcester.

Overview

Obtain sputum with induced sputum procedure (2 cups) (***See Sputum Induction Procedures section of the Sample Collection Handbook for detailed procedures)***
Store samples in the interim at the study clinic
Transport samples to NHLS Worcester
Retrieve and input data into REDcap from NHLS results
Results

Sputum collected for MTB/RIF Ultra testing should be conducted at Aim 1 Visit 1. The results of the testing should be provided to the participant at the end of their Aim 1 Visit 2, when results are in, along with linkage to TB clinical care *(**See Appendix II**: Linkage to TB Care).*

Please note that participants with an Aim 1 Visit 1 negative Xpert and culture will be asked to provide additional samples at their Aim 1 Visit 2 (see flowchart for sputum collection above). If the second round of samples are negative, the participant does **not** need to be contacted further, and are not eligible for any of the subsequent aims. If any of the round two samples are positive, the participant **must be** traced in the community and brought in to receive their results and be linked to care. These participants will also be eligible for enrollment into Aim 2 and possibly Aim 3.

Sputum Sample Collection, Processing and Interim Storage

Induced Sputum Supply List

**Item**	**# Per Collection**
Sputum Cup	1
Biohazard bag	1

Nebulizer	1
Disposable nebulizer tubing and mask	1
3% hypertonic saline solution (20 mL)	May need several 20 mL bullets for complete procedure

Sample Labels

**STS Sample Type**	**Sample ID**	**Sample Purpose**	**#Labels Needed Per Collection**
TOTAL_XPERT	TXPERT. __ __ __ __ __. __ __<br>(P) (4# PID) (#Xpert Sample)	GeneXpert Ultra MTB/RIF testing	1

Xpert Ultra testing is only performed on participants in Aim 1. Labels should be pre-printed with TXPERT and 7 underscores for labelling by study staff. Sputum for Xpert Ultra is labelled TXPERT, the participants PID, and the sequential number sample this is for Xpert Ultra testing for that participant. For example, the first sputum for Xpert Ultra testing for participant P0099 will be labelled TXPERT.P0099.01. If this participant provides a second sample because their first sputum samples were negative, their second sample for Xpert Ultra testing will be labelled TXPERT.P0099.02. The label must also include the date of the sputum collection (dd/mm/yyyy).

<u>Procedures</u>

See Sputum Induction Procedures section of the Sample Collection Handbook *for full supply list and detailed procedures.*

Briefly, samples are collected via sputum induction. Each time sputum is collected, for any reason, the Sputum Specimen Collection DCF must be completed. Samples must be properly labelled and checked-in to the TOTAL Sample Tracking System immediately after collection.

<u>Sample, Test Kits, and Supply Disposal</u>

Store all supplies at room temperature (mask, tubing, cups, and saline solution). Do not let saline solution be exposed to sunlight or prolonged heat.

All materials used during sputum induction, including: gloves, tubing, masks, etc. need to be disposed of in a biohazard waste container. ***See SOP: Procedures for Biohazard Disposal*** for additional information.

<u>Short Term Storage at the Study Clinic</u>

Sputum samples for GeneXpert ultra will be stored at the study clinic site in a sample specific refrigerator (8 ˚C). Samples should be transported to NHLS at the **end of each day**. If NHLS is unable to collect samples for any reason that day, samples must remain stored in the specimen refrigerator at the study clinic until they can be brought to NHLS, which must occur on the **next possible day.**

Transport to NHLS
Sputum samples for GeneXpert Ultra should be transported from the study clinic to NHLS Worcester at the end of each day unless NHLS is unable to receive samples that day, in which case they remain in the study clinic refrigerator before transport the next day.

1. The sputum samples should be packed in accordance with South African guidelines on infection control. They should be packed in a cooler box with enough cold packs sufficient to maintain a 2 – 8 ˚C temperature range during transport.
2. The samples will be transported to NHLS and sample hand-off will occur following procedures to be determined.

Laboratory procedures for GeneXpert Ultra MTB/RIF testing and cycle threshold
Sputum samples will be run by NHLS for *Mtb* presence (High, Medium, Low, Very Low, Trace, Not Detected), RIF susceptibility, and cycle threshold results.

GeneXpert Ultra Results Data Retrieval and Upload
We will access participant NHLS Xpert Ultra results from the NHLS data portal (insert website here). Information for logging in to the NHLS portal will be given to the appropriate team members by either the SUN Junior Scientist or Boston based Project Manager. Data, including *Mtb* detection, RIF susceptibility, and cycle threshold values, will be added to the TOTAL REDCap database **Xpert Results DCF** by the research team.

Preparing a shipment inventory

To create a shipment (shipment in this context indicates samples for transport) in the database application, the field coordinator will run the **Xpert Shipping Report** on REDCap which includes a listing of the barcodes available for shipment and the corresponding participant IDs. The team member responsible for preparing the shipment will check-out each sputum sample in the Sample Tracking System for Xpert listed on the Xpert Shipping Report.

The team member must generate a Shipment Inventory Form (See Form: Shipment Inventory Form in Dropbox) for the sputum for Xpert samples that contains: details on type of samples (PAXgene, sputum etc.) date of shipment, transporter initials, and the Xpert Shipping Report, and print and include these documents in the shipment container.

When site personnel prepare a shipment for transport, the Field Coordinator must notify the receiving lab via e-mail with a summary of the contents of the shipment, the expected time of arrival of the shipment, and an electronic version of the Xpert Shipping Report listing the specimen IDs (and associated participant IDs) included with the shipment.

Packaging the shipment

See ***SOP: Specimen Packing for Transport*** for how to pack sputum specimens for transport to NHLS.

11.3 Sputum Samples for *Mtb* Culture and Whole Genome Sequencing

Sputum samples are collected for *Mtb* culture for TB testing during Aim 1 (up to 2 samples for *Mtb* culture are collected in Aim 1) (see Diagram 1: Flow Chart for Sputum Collection above). Sputum samples for *Mtb* culture are also collected from participants enrolled in Aim 2 and Aim 3 Arm 2 for whole genome sequencing of the TB bacteria.

<u>Overview</u>

Obtain sputum with induced sputum procedure (***See Sputum Induction Procedures section of the Sample Collection Handbook for detailed procedure)***
Store samples in the interim at the study clinic
Transport samples to Stellenbosch University
Short-term storage at Stellenbosch University
Sample culture via MGIT (Aim 1, one sample from Aim 2, and one sample from Aim 3 Arm 2)
Store whole sputum (one sample from Aim 2, and one sample from Aim 3 Arm)
DNA extraction at Stellenbosch University from MGIT and whole sputum (Aim 2 and Aim 3 Arm 2)
Transport DNA from culture and whole sputum (Aim 2 and Aim 3 Arm 2) to TGen, Arizona, USA
Long-term storage of cultures at Stellenbosch University, Prof Rob Warren's culture bank

<u>Results</u>

Sputum collected for *Mtb* culture for the purpose of TB diagnosis is conducted at Aim 1 Visit 1. Sputum results are entered into the **Culture Results DCF** by the SUN lab technologist. Positive culture results should be given to participants once results are available, along with linkage to TB clinical care ***(See Appendix II: Linkage to TB Care).*** A participant may be negative on GeneXpert Ultra but positive on culture. It is best to indicate to participants at Aim 1 Visit 2, who are negative with GeneXpert that we will contact them when their culture results come in, if their culture results are positive for TB. Sputum is collected at Aim 1 Visit 2 if the samples from visit 1 are negative for TB testing on Xpert and mtb culture. If either of the participants round 2 samples are positive, the participant must be contacted and asked to return for a TB Results Visit, where we will provide results and link to TB care. At this time the participant will also be eligible for Aim 2 and possibly Aim 3.

Sputum is also collected and cultured in Aim 2 for DNA extraction from MTB culture for whole genome sequencing, the results of whole genome sequencing are **not** provided to participants.

<u>Sputum Sample Collection, Processing and Interim Storage</u>

*See **Sputum Induction Procedures section of the Sample Collection Handbook** for full supply list.*

Sample Labels

**STS Sample Type**	**Sample ID**	**Sample Purpose**	**# Labels Needed Per Collection**
TOTAL_SPUTUM_AIM1_CULTURE	TCUL. __ __ __ __ __ . __ __<br>P (4 # PID) sample #	*Mtb* culture for TB diagnosis	1

TOTAL_SPUTUM_ WGS_CULTURE	TWGSCUL. __ __ __ __ __ . __ __ P or C (4 # PID) sample #	Sputum for DNA extraction and whole genome sequencing from culture	1
TOTAL_SPUTUM_ WGS_DIRECT	TWGSDIR. __ __ __ __ __ . __ __ P or C (4 # PID) sample #	Sputum for DNA extraction and whole genome sequencing directly from sputum	1

Labels should be pre-printed with TCUL, TWGSCUL, and TWGSDIR and 7 underscores for labelling by study staff. Sputum for mtb culture is labelled TCUL, TWGSCUL, or TWGSDIR, the participants PID, and the sequential number sample this is for mtb culture in that Aim for that participant. For example, the first sputum for mtb culture for TB testing (AIM 1) for participant P0099 will be labelled TCUL.P0099.01. If this participant provides a second sample because their first sputum samples were negative, their second sample for mtb culture for TB testing will be labelled TCUL.P0099.02. If this participant enrolls in AIM 2 and provides 2 sputum's for whole genome sequencing, the samples will be labelled TWGSCUL.P0099.01 and TWGSDIR.P0099.01 and for those in Aim 3 Arm 2 who provide sputum for WGS, TWGSCUL.C0010.01 and TWGSDIR.C0010.01 for example. The label must also include the date of the sputum collection (dd/mm/yyyy).

Procedures

*See **Sputum Induction Procedures section of the Sample Collection Handbook** for detailed procedures.*

Sample, Test Kits, and Supply Disposal

Store all supplies at room temperature (mask, tubing, cups, and saline solution). Do not let saline solution be exposed to sunlight or prolonged heat.

All materials used during sputum induction, including: gloves, tubing, masks, etc. need to be disposed of in a biohazard waste container. ***See SOP: Procedures for Biohazard Disposal*** for additional information.

Short Term Storage at the Study Clinic

Sputum samples for culture will be stored at the study clinic site in a sample specific refrigerator (8 ˚C).

1. Double-check the label on the sputum container to ensure that specimen ID and dates and times have been completed appropriately and legibly. If not, if you were the nurse who collected the sample, please re-label, or find the nurse who did collect the sample to re-label.

*Note: If the nurse indicates that he/she did not collect a sputum sample, do not add the sample ID into the Sample Tracking System or forward the sample for analysis; it should be returned to storage as an unused sputum container. On the REDCap **Sputum Specimen Collection DCF** indicate that no sputum was collected and provide a reason/comment on the form.*

2. Store the sputum specimens, in their individual biohazard bags, in a 2 – 8˚C refrigerator

Transport to Prof Warren's Lab, Stellenbosch University, Tygerberg, Cape Town

Sputum samples for *Mtb* culture must be transported to Prof Warren's Lab once **every 3 days.**

1. The sputum samples should be packed in accordance with South African guidelines on infection control. They should be packed in a cooler box with enough cold packs sufficient to maintain a 2 – 8 ˚C temperature range during transport.
2. The samples will be transported to Prof Warren's Lab at Stellenbosch University, and sample hand-off will occur following procedures to be determined.

Laboratory Procedures for *Mtb* Culture for Aim 1
Sputum samples will be run by the laboratory technician at Stellenbosch University and analyzed by MGIT for appearance, growth, concentrated and MGIT ZN, contamination (smear microscopy and blood agar), and confirmatory capillia testing for contaminated samples. Samples with *Mtb* growth will also receive DST/MIC for EMB, RIF, INH, and PZA. Full SOP for sputum culturing is available in the **TOTAL Laboratory MOP.**

Laboratory Procedures for *Mtb* Culture for Aim 2
Specimens collected in Aim 2 are cultured and stored for DNA extraction for eventual transport and whole genome sequencing at TGen in Arizona, USA. Standard culturing techniques as described in the **TOTAL Laboratory MOP** are to be followed.

Mtb Culture (Aim 1) Results Data Retrieval and Upload
Mtb culture results for Aim 1 will be entered into the REDCap database, **Sputum Results DCF**, by the Stellenbosch Laboratory Technician. Data to be entered include sputum sample quality, MGIT growth results, ZN smear results, DST/MIC, etc.
Sputum Banking of *Mtb* Culture (Aim 1)
Sputum samples from Aim 1 that have *Mtb* growth will be stored at Prof Robin Warren's sputum bank once all analyses for TOTAL have been finished. Cultures are to be stored according to **Sputum Culture Long Term Storage Procedures** detailed in the **TOTAL Laboratory MOP.**

Interim Storage of *Mtb* Culture (Aim 2)
Mtb cultures will be stored at Stellenbosch University until DNA extraction and transport of mtb DNA to TGen.

For full material lists and detailed procedures, see **TOTAL Laboratory MOP**.

Transport of DNA from *Mtb* Culture (Aim 2) to TGen, Arizona
Mtb DNA will be transported to TGen upon execution of a Material Transfer Agreement and the obtainment of a South Africa Export Permit. Detailed procedures for airline transport will be defined at a later date, but will follow IATA regulations.

Laboratory Procedures for WGS from Culture at TGen
DNA will be analyzed for *Mtb* whole genome sequencing. Detailed procedures from TGen are to be defined at a later date.

WGS from Culture Results Data Retrieval and Upload
Whole genome sequencing data will be provided by TGen to the participating institutions via a method to be determined.

Preparing a shipment inventory

To create a shipment (shipment in this context indicates samples for transport) in the database application, the field coordinator will run the Sputum Shipping Report on REDCap which includes a listing of the Sample IDs available for shipment and the corresponding participant IDs. The team member responsible for preparing the shipment will check-out each sputum sample in the Sample Tracking System for sputum listed on the Sputum Shipping Report.

The team member must generate a Shipment Inventory Form (See Form: for Shipment Inventory Form) for the sputum for MTB culture samples that contains: details on type of samples (PAXgene, sputum etc.) date of shipment, transporter initials, and the Sputum Shipping Report, and print and include these documents in the shipment container.

When site personnel prepare a shipment for transport, the Field Coordinator must notify the receiving lab via e-mail with a summary of the contents of the shipment, the expected time of arrival of the shipment, and an electronic version of the Sputum Shipping Report listing the specimen IDs (and associate participant IDs) included with the shipment.

Confirmation of Received Samples
The lab technologist receiving the sputum samples must navigate to the participants **Sputum Results Form (Lab)** on REDcap and enter that the specimen was received and the date received.

Packaging the shipment
<u>Sputum Transport from Study Clinic to Stellenbosch, Tygerberg Campus</u>

See ***SOP: Specimen Packing for Transport*** for how to pack sputum specimens for transport to Tygerberg.

<u>MTB DNA Transport from Stellenbosch, Tygerberg to TGen, Arizona, USA</u>

The following shipping materials are required for shipment of stored *Mtb* DNA from Stellenbosch University to TGen:

Item	Vendor	Catalog #	# Per Shipment
TBD			

Shipments must be packed according to IATA regulations. Detailed procedures to be added.

11.4 Exhaled Bioaerosols Collection

Aim 3 Arm 1 and Aim 3 Arm 2

Exhaled bioaerosols are collected with the Respiratory Aerosol Sampling Chamber (RASC) (see image below). The RASC is located on the Worcester CDC grounds. Participants wear a specialized suit (see image below) and are instructed to sit in the RASC for 60 minutes while the sampling of their natural breathing occurs. The RASC is equipped with a small television for the participants to watch during the procedure. Detailed procedures must be followed when conducting the procedure see **SOP: RASC Bioaerosol Collection** and for sterilizing the RASC after use, see **SOP: Sterilizing RASC after Use.**

Image: RASC Sampling Chamber

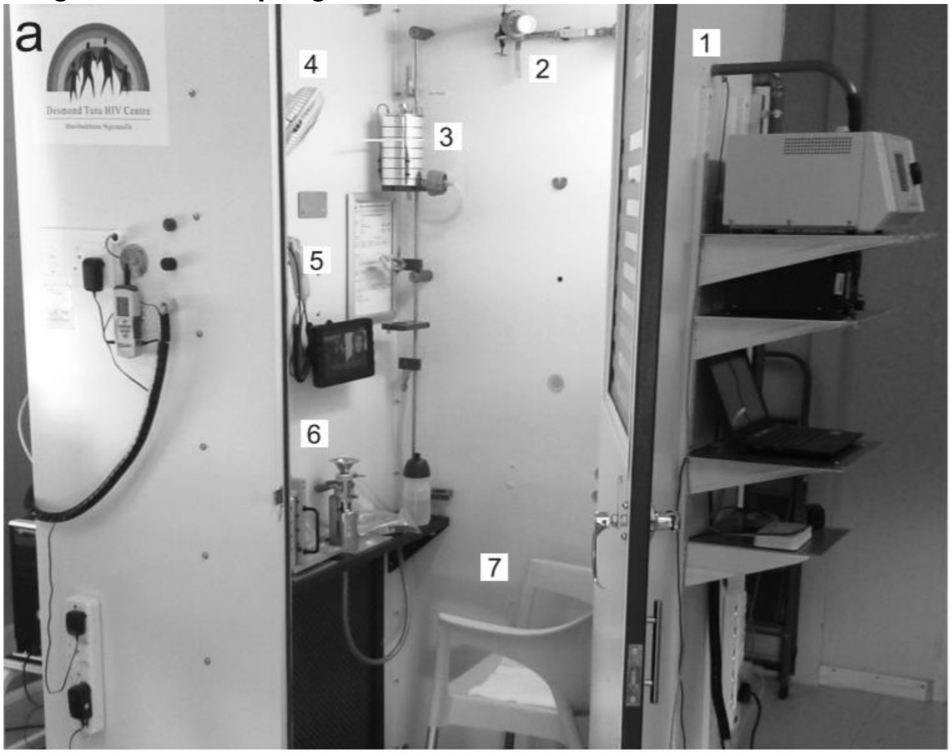

Image: RASC Specialized Suit for Participant

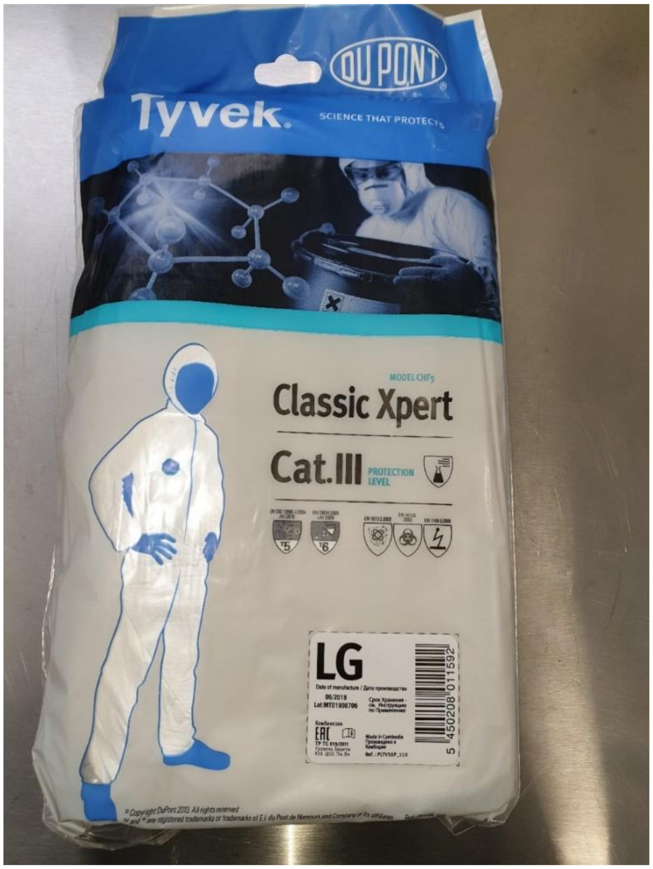

<u>Overview</u>
Staff must be properly trained before conducting RASC procedure
RASC procedure conducted at Worcester CDC grounds
Sample stored in interim at study clinic
Sample transported to ***TBD*** for handover to Desmond Tutu team

11.4.1 Exhaled Bioaerosols Collection Procedure

See **SOP: RASC Bioaerosol Collection** for detailed list of supplies and step-by-step instructions on the sampling procedure.

11.4.2 Exhaled Bioaerosols Collection Procedure Dedicated Staff Training

Before conducting their first bioaerosol collection, study staff must be trained. Initially, the team at Desmond Tutu HIV Foundation will train the field coordinator, RNs, and RAs on the procedure. Following the initial training, if there is any staff turnover, it is the responsibility of the field coordinator to ensure the new staff member is trained. The staff member must observe at least 3 bioaerosol collections before being permitted to lead the procedure. These 3 observed procedures must be documented and initialled by the new staff member and the staff member they observe. Upon finishing their 3rd observation they should submit their form to the field study coordinator who will sign and store the form in a dedicated training binder.

11.4.3 Receipt, Handling, and Storage of Exhaled Bioaerosols

See **SOP: RASC Bioaerosol Collection for detailed list of supplies** needed for procedure.

Aim 3 Arm 1 and Aim 3 Arm 2

The following table lists the TOTAL barcode labels needed for the exhaled bioaerosol collection (generated through ST module):

ST Sample Type	Barcode Number	Sample Purpose	# Labels Needed Per Collection
TOTAL_AERO	AERO.__ __ __ __ __. __ __<br>P or CT, 4 # PID, Sample #	Exhaled bioaerosol *mtb* analyses	1

Labels should be pre-printed with AERO and 7 underscores for labelling by study staff. Bioaerosols are labelled AERO, the participant's PID, and the sequential number bioaerosol sample this is (which almost always will be 01, one sample per participant).

11.4.4 **Short Term Storage of Collected Samples**

This section is TBD.

11.4.5 Transport Instructions

This section is TBD.

11.4.6 Results

Desmond Tutu HIV foundation will conduct all analyses on the exhaled bioaerosols. Results will be shared with the other investigators via a method to be determined. Results will not be given to study participants.

11.5 Urine Sampling

Urine samples are collected for drug testing and pregnancy testing. For Aim 1 participants and Aim 3 Arm 2 participants, we will store 50 mL of urine in plastic vials for future analyses.

Overview

Obtain urine in a specimen cup (***See Urine Collection section of the Sample Collection Handbook for detailed procedure)***
Perform necessary testing pending study visit
Transfer 50 mL of urine to plastic vial
Short-term storage at study clinic
Transport to SUN Tygerberg
Long-term storage at SUN Tygerberg

Results
Participants are provided with pregnancy testing results but not drug testing results.

Urine Sample Collection, Processing and Interim Storage

*See **Urine Collection section of the Sample Collection Handbook** for full supply list.*

Sample Labels

**STS Sample Type**	**Sample ID**	**Sample Purpose**	**# Labels Needed Per Collection**
TOTAL_URINE	TURINE. __ __ __ __ __ . __ __<br>P (4 # PID) sample #	Urine for pregnancy and drug testing.	2, one for sputum cup and one for 50 mL vial

Labels should be pre-printed with TURINE and 7 underscores for labelling by study staff. The label must also include the date of the sputum collection (dd/mm/yyyy).Both the specimen collection cup and 50 mL vial must be labelled the same.

Procedures

Urine will be collected for drug and pregnancy testing into a specimen cup.
After the participant visit, using a plastic funnel, fill a 50 mL labelled vial with the urine.
This urine will be transported to SUN Tygerberg for future analyses.

Sample, Test Kits, and Supply Disposal
Drug Kits/Test Strips: Store at room temperature in the sealed pouch up to the expiration date. Keep away from direct sunlight, moisture and heat. DO NOT FREEZE.

Pregnancy Tests: Store pregnancy tests at normal room temperature. Discard unused pregnancy tests after the expiration date, which is stamped on each sealed pregnancy test. Avoid prolonged exposure to direct sunlight, moisture, heat, and other related conditions.

All materials used during sputum induction, including: gloves, tubing, masks, etc. need to be disposed of in a biohazard waste container. ***See SOP: Procedures for Biohazard Disposal*** for additional information.

Short Term Storage at the Study Clinic
Urine sample after they have been used for their TOTAL procedures will be stored at the study clinic site in a sample specific refrigerator (8 ˚C). Urine will be transported with sputum at a maximum of every 3 days.

1. Double-check the label on the container to ensure that specimen ID and dates and times have been completed appropriately and legibly. If not, if you were the nurse who collected the sample, please re-label, or find the nurse who did collect the sample to re-label.
2. Store the urine specimens, in their individual biohazard bags, in a 2 – 8˚C refrigerator
3. After the study visit, transfer the urine using a funnel to a pre-labelled 50 mL vial.
4. Store the 50 mL vial in an individual biohazard bag in the refrigerator.

Transport to Prof Warren's Lab, Stellenbosch University, Tygerberg, Cape Town
Sputum samples for *Mtb* culture must be transported to Prof Warren's Lab once every 3 days.

3. The sputum samples should be packed in accordance with South African guidelines on infection control. They should be packed in a cooler box with enough cold packs sufficient to maintain a 2 – 8 ˚C temperature range during transport.
4. The samples will be transported to Prof Warren's Lab at Stellenbosch University, and sample hand-off will occur following procedures to be determined.

Laboratory Procedures for Urine
Future laboratory analyses are TBD.

Preparing a shipment inventory

To create a shipment (shipment in this context indicates samples for transport) in the database application, the field coordinator will run the Urine Shipping Report on REDCap which includes a listing of the Sample IDs available for shipment and the corresponding participant IDs. The team member responsible for preparing the shipment will check-out each sputum sample in the Sample Tracking System for sputum listed on the **Urine Shipping Report.**

The team member must generate a Shipment Inventory Form (See Form: for **Shipment Inventory Form**) for the sputum for MTB culture samples that contains: details on type of samples (PAXgene, sputum etc.) date of shipment, transporter initials, and the Sputum Shipping Report, and print and include these documents in the shipment container.

When site personnel prepare a shipment for transport, the Field Coordinator must notify the receiving lab via e-mail with a summary of the contents of the shipment, the expected time of arrival of the shipment, and an electronic version of the Urine Shipping Report listing the specimen IDs (and associate participant IDs) included with the shipment.

Packaging the shipment

Urine Transport from Study Clinic to Stellenbosch, Tygerberg Campus

See ***SOP: Specimen Packing for Transport*** for how to pack urine specimens for transport to Rob Warren Lab.

12 Chest X-Ray

On the day of their Aim 3 visit, a study research assistant will accompany the participant to Mediclinic for a chest X-ray. Mediclinic will provide the study with the X-ray image and interpretation for each participant via a CD. If a participant is unable to go to Mediclinic for a chest X-ray on the day of their visit, every effort should be made to find a time for them to have an X-ray taken within one week of treatment initiation.

13 TOTAL Lab Addresses and Contact Personnel for Shipping/Transport

Lab	Contact Name	Lab Address	Phone Number	Email	Sample Type
Rob Warren Lab	Noorjahn Rawoot<br><br>Nandi Niemand	ROOM RM5026, LEVEL 5, TEACHING BUILDING<br><br>BMRI, Office 2030, Lab 2.2	0219389409<br><br>0837562707	noorjahn@sun.ac.za<br><br>nniemand@sun.ac.za	Sputum for MTB culture and WGS<br><br>Blood for COVID serology<br>Urine
Gerhard Walzl Lab	Ilze Louw	Fisan Building, K Floor	0 21 938 9839	Ilzel@sun.ac.za	PAXgene
NHLS	Natalie Nissen	Worcester Hospital	023 348 1402	natalie.nissan@nhls.ac.za	Sputum for Xpert
Robin Wood Lab					RASC

14 Participant Visits for Collecting Missed Samples and Sample Collection Window's

There may be occasions when a participant is unable to provide a sample at their scheduled visit.

Possible reasons include:

- For Blood Draws: dehydration leading to the inability to draw up blood

- For Sputum Induction: adverse reaction to the procedure including wheezing, coughing, or dyspnoea, which does not resolve within 30 minutes of the procedure.

Other reasons not described above may occur, including reasons that affect the ability to conduct the exhaled bioaerosol collection or chest x-ray.

If a participant does not complete a component of their study visit, every attempt should be made to reschedule that procedure. However, due to the nature of the study, some procedures need to be completed in a certain time-frame. See below for time-frame for all procedures:

- Blood draw for HIV testing (Aim 1 V1) - Blood can attempt to be drawn whenever a participant comes back to pick up reimbursement for coupons, or any subsequent study visit.
- Blood draw for PAXgene (Aim 1 Visit 1) - Blood can attempt to be drawn when the participant comes back to pick up reimbursement for coupons, and at the latest **1 week after TB treatment initiation.**
- Sputum for GeneXpert (Aim 1 Visit 1 and Aim 1 Visit 2 if required) - Sputum is required for TB diagnosis. Our questionnaires are to be administered on the same day that sputum is taken. Sputum should always be taken before questionnaires are administered, and if sputum can not be collected **the entire visit should be rescheduled.** The induced sputum procedure should never be attempted more than twice in one day.
- Sputum for *Mtb* culture for diagnosis (Aim 1 Visit 1 and Aim 1 Visit 2 if required) -This sample is collected at the same time as the sputum for GeneXpert. Our questionnaires are to be administered on the same day that sputum is taken. Sputum should always be taken before questionnaires are administered, and if sputum can not be collected **the entire visit should be rescheduled.**
- Sputum for *Mtb* culture for WGS (Aim 2) – Once participants are diagnosed with TB, it is likely they will start treatment within 1 week. We must collect sputum for WGS **at a maximum of 5 days after treatment initiation.**
- Sputum for WGS from direct isolate (Aim 2) – Once participants are diagnosed with TB, it is likely they will start treatment within 1 week. We must collect sputum for WGS **at a maximum of 5 days after treatment initiation.**
- Exhaled bioaerosol collection (Aim 3 Arm 1 and 2)- Every attempt should be made to conduct the RASC procedure **before** the participant has started TB treatment, it can be collected at a maximum of **5 days after treatment initiation**
- Chest X-ray (Aim 3 Arm 1 and Aim 3 Arm 2): The chest x-ray can be collected up to **one week** after tuberculosis treatment initiation.

If there is ever a question about visit windows or whether you may still collect a sample, the BMC project manager must be contacted ASAP with details of the request.

If any samples are missed and unable to be collected, subsequent samples should still be collected and submitted for analysis.

15 TOTAL Questionnaires

The TOTAL questionnaires for all visits will be administered verbally to participants with the responses entered via electronic data capture on REDCap. The questionnaires will be in English, and any open-ended responses should be written in English as well. There will be paper translations of all questionnaires available in isiXhosa and Afrikaans for the interviewer to read verbatim from when administering the survey. All changes made to surveys will be tracked with REDCap's internal audit trail. Anytime a question is changed on REDCap after a questionnaire has been saved, an accompanying comment on the field (using the REDCap comment option) **must** be made explaining the change. See the **TOTAL Data Management/CQMP** for details about using REDcap and QC/QA procedures.

All staff responsible for conducting participant interviews will be trained to approach participants in a friendly, respectful manner so that they feel comfortable. Staff will be trained by the SAMRC PI in working with participants with substance use, including stigma training. Staff will be trained to remain neutral when asking questions and recording responses. This will help with collection of accurate data. At the beginning of the interview, the participant will be reminded of how long the interview will take to complete. Participants will be asked to confirm which language they prefer the interview (English, Afrikaans, isiXhosa) and the preferred language will be used. If a participant is confused about a question or task, the interviewer will be trained to stop and ask them if they understand. If the interviewer is still unsure of whether they understand or not, the participant will be asked to explain the question to the interviewer in their own words. Interviewers will be trained to identify inconsistencies in a response set. If a participant says one thing for one question, and then answers completely differently for another question on the same topic, the interviewer will stop and try to see if the participant understood the question. If necessary, the interviewer will go back to the previous question and check their answer. At certain times, participants may become less willing to answer questions, participate in the session or they may seem to just be giving an answer to finish quickly. Interviewers will be trained to pause the interview and ask the participant if they are alright or if they need a break. In addition, staff will be trained to read the questions exactly as they are written and accurately record participants' answers as stated by the participant. If a participant changes their response, the original answer will be crossed out and initialled/dated if on paper, or changed to the correct response if direct entry on REDCap.

15.1 Aim 1 Visit 1

- Aim 1 Screening Survey – The screening survey is completed after pre-screening and consent to assess final eligibility for the Aim 1 Visit, including results of the urine drug testing.

- Demographic Survey – The demographic survey collects self-reported information on race, gender, age, employment status, household conditions, etc.

- Medical History Survey- The medical history survey was created for TOTAL to collect information on comborbities, concomitant medications, HIV status, and TB history. This survey is initially filled out through participant self-report. HIV status and ART status is verified by HIV blood testing and the participant's medical record. Self-reported current TB

diagnosis is also verified by the medical record, including information on the participant's current TB treatment plan.

- Important People Instrument- This survey has been modified for our study setting and assesses meth/Mandrax and other substance use within the participant's social network.

  The IPI is an interviewer-assisted instrument designed to be utilized in a face to face interview format and requiring, on average, approximately 12 minutes to complete. There are specific guidelines and tips for each question provided by the creators of the instrument in ***SOP: Important People Instrument: How to Administer.*** These guidelines must be followed when administering the survey.

- The Alcohol, Smoking and Other Substance Involvement Screening Tool (ASSIST) - This is a widely used survey which assesses the extent of substance dependence and risk of harms.

  This is a validated survey and designed to be administered face to face. The interviewer must read the questions as they are written. There are direction boxes on the survey with instructions for the interviewer and additional instructions that should be read aloud to the participant. The participants must be given the ASSIST Response Card to facilitate their responses to the questions *(See Form: ASSIST Response Card)* in Dropbox folder Logs and Forms.

- RDS Network Size- This survey is critical in estimating the participant's network (defined as those who use illicit drugs, 15 years or older) size for RDS data analysis.

  It is important to give participants enough time to think about their responses thoughtfully. Participants should be given a sheet of paper pre-numbered to write their responses out and then to verbally give the interviewer the answer to enter into REDCap. The interviewer can ask clarifying questions on answers the participant seems unsure about.

  For example, Q1 "Think about the people in Worcester, who you know by name and they know you by name. Of these people, think about the ones who use Tik and/or Mandrax. How many people are these?"

  If the participant responds, 0, you may want to ask about their recruiter, who knew them enough for them to recruit you, and this person also uses tik/mandrax. You can also tell the participant to think about friends, family members, or other people in the community who they use with or they have bought substances from, those people should be included here.

  For Q2 "Of these ____ people, how many are 15 years or older (still in school/school age)?" the interviewer must fill the blank with the number the participant provided in Question 1. If the participant is unsure, or does not know the people in Q1s ages, ask them to think about which ones are still in school, versus those that are older. You can also probe them on which of those in Q1 are "younger kids" versus teenagers or adults.

  Q3 "Of these ____ people, how many have you seen in the last 1 month?" the interviewer must fill in the blank with the number from Q2 (those the participant knows who are 15 or

over). The participant may be probed here. Ask them to think about who they have seen, even briefly, to buy substances, or if they saw someone who use substances but did not use together, they should still be included. This is anyone they know who is over 15 who uses substances who they saw, even briefly.

- Timeline Follow Back (TLFB) – This survey will be used to assess meth/Mandrax behavior in the past month

  The timeline follow back must be accompanied by a calendar marking the previous 2 weeks (the 2 weeks before the current visit). The timeline follow back is only completed for participants who admit to use in the past 2 weeks. The participant and the interviewer will work together to go through each day to outline which drugs were smoked that day and to quantify the amount. Quantifying the daily intake will involve calculating the number of times that drug was smoked that day.

  For detailed instruction on administering the TLFB, see ***SOP: Timeline Follow Back (TLFB): How to Administer.***

- TB Symptom Screen- We will use the WHO four-symptom screening to evaluate for clinical versus subclinical TB; participants will be asked about current cough (including hemoptysis), a cough lasting ≥2-3 weeks, weight loss, night sweats, or fever.

  The TB symptom screen will assess current TB symptoms. The participant can be probed if they answer yes to a question but do not know for how many days. Helpful probes are: "did you have ___ last weekend? Did you have it last month?" If there was a recent holiday, you can ask if they had the symptom on that day, and if so, how long did they have it before then? From here you can begin to calculate how long they had the symptom for.

- Social Support Survey- the Medical Outcomes Survey Social Support measure is a well-established instrument that consists of 19 items, and measures: (1) emotional support, (2) informational support, (3) tangible support, (4) positive social interaction, and (5) affectionate support.

  The social support survey probes the extent to which an individual has access to social support in a variety of situations and may be uncomfortable for some participants to answer. If a participant does not answer the question the interviewer may repeat it, but the participant has the option to skip questions if they do not feel comfortable responding. It is important that this survey is asked later in the line of questionnaires once a level of comfort and trust has been established between the interviewer and participant. Again, establishing a welcoming, open, and comfortable atmosphere is critical for the administration of the study questionnaires.

- Household Hunger Scale- the Household Hunger Scale is a 3-item instrument used to assess food deprivation and security.

  Because the HHS questions cover topics about which respondents may be sensitive, it is recommended that the HHS module be placed towards the end of the survey instrument, to

be administered after a certain degree of rapport has been established between the interviewer and the participant.

USAID provides the following guidelines for administering the HHS:

Although there are pre-coded response options, these should be read only for the first HHS question, as suggested response options. The respondent should be allowed to answer in his or her own words. The enumerator will then select the most appropriate response option based on the respondent's reply. For instance if, after asking an occurrence question, the respondent says "no," but adds that it only happened a few times, then the correct code is "1" (yes). The frequency-of occurrence question should then be asked. If the respondent describes a frequency that would translate to "3–10 times" in the past 30 days, the correct response selection for the frequency-of-occurrence question is "sometimes," and the correct response code is "2." If the respondent has difficulty replying, then the interviewer can encourage a response by listing the set of options again.

For the USAID full report on how to administer the HHS see ***SOP: Household Hunger Survey: How to Administer.***

- Center for Epidemiologic Studies Depression Scale (CES-D) – The CES-D is a 20-item scale which will be administered to measure depressive feelings and behaviours during the past week.

  The CES-D should take approximately 10 minutes to complete. Have the participant report on how often he/she's experienced the following 20 feelings within the past week. Study staff will read the questionnaire aloud and guide the participant through each item and answer. Please encourage participant to respond to all items, however a participant has the right to skip a question if they choose.

- Kessler Psychological Distress Scale (K10)- This is a 10-item questionnaire intended to yield a global measure of distress based on questions about anxiety and depressive symptoms that a person has experienced in the most recent 4 week period. Please encourage participant to respond to all items, however a participant has the right to skip a question if they choose.

- HIV Risk Survey- The HIV Risk-taking Behaviour Scale (HRBS) is a brief, 11 item questionnaire developed to measure the behaviour of people who inject drugs that puts them at risk of either contracting, or passing on, human immunodeficiency virus (HIV). Two predominant areas of concern exist in relation to the spread of HIV amongst this population and, via this route, to the broader community: injection use behaviour and sexual behaviour. Please encourage participant to respond to all items; however a participant has the right to skip a question if they choose.

- Gang Association Survey- this survey assesses participant gang affiliation and knowledge of if family/friends are in gangs. Please encourage participant to respond to all items, as it is important to find out about these activities, which were prevalent in our formative work.

.

15.2 Aim 1 Visit 2

- Follow up RDS Interview (Post RDS Rejection Recruitment Questionnaire):

At Aim 1 Visit 2 and **all** visits where a participant comes to collect reimbursement for successfully recruited peers, the follow up Post RDS Rejection Recruitment Questionnaire will be administered. This questionnaire collects information on who the participant tried to recruit, and if any people were not interested, why they were not interested.

15.3 Aim 2 Visit

- Aim 2 Screening Survey:

The screening survey is completed to confirm final eligibility for the Aim 2 Visit, including reporting evidence of active TB disease.

- Social Contact Survey:

The Social Contact Survey was originally developed to investigate a TB outbreak in Canada- and has been modified for use in South Africa. We further adapted for our study setting to include substance use. The survey must be administered with a map to identify locations specified by the participant and mark their full address or GPS coordinates on the questionnaire. Because the questionnaire is long, for time saving it is best to mark the spot on the map with the question number, and go back after the visit has ended to capture the address or GPS coordinates.

15.4 Aim 3 Arm 1 Visit

- Aim 3 Screening Survey

The screening survey is completed to confirm final eligibility for the Aim 3 Visit, including pregnancy-testing results.

15.5 Aim 3 Arm 2 Visit

Participants in Aim 3 Arm 2 are administered the same surveys as those in Aim 1 and Aim 2 except for the RDS Questionnaires (Network Size and Coupon Rejection Survey), Important People Instrument, and the Timeline Follow Back (for Meth and Mandrax). These participants are asked the ASSIST, to assess alcohol, tobacco and other drug use. The Aim 3 Arm 2 Screening Form is the only new survey that needs to be filled out for these participants.

16 Siblings, Biological Relatives and Housemates (Aim 1 Visit 1)

There are no eligibility exclusions for enrollment of siblings, other biological relatives, or individuals who are not related but share the same household. Each participant will be evaluated for study eligibility and allowed to enroll if all criteria are met.

In order to accurately measure network linkages related to drug use versus linkages related to household contacts, it is necessary to identify enrolled participants who share a household. The Demographic Survey question on recruiter relationship facilitates the identification of important relationships by asking questions about their relationship with their peer recruiter. However, if

household contacts were never linked through coupons by peer recruitment, we would not know that two participants share a household.

To accurately capture participants who share a household, we will utilize the **Locator Form.**

17 Medical Record Data Abstraction

In TOTAL, most data is captured via case report forms collected using our IRB approved TOTAL questionnaires. Some data captured on these tools may be subject to inconsistencies that we can verify using the participant medical record. The questions that need verification with the medical record are indicated on the **Medical History Survey.**

The participant medical record must attempt to be collected for participants who:

1. Have a history of HIV, or are newly diagnosed with HIV through our testing
2. Have a history of TB, attend their first visit already sick with Tuberculosis, or are newly diagnosed with Tuberculosis through our testing.
3. Have a history of COVID-19 diagnosis

If a participant was diagnosed with HIV, TB, or COVID-19 before enrolling in TOTAL in a clinic not located in Worcester, we may be unable to access their medical record to verify their data. For participants diagnosed in a clinic in Worcester, every attempt should be made to access their medical record for data verification. For participants diagnosed outside of TOTAL, confirm with the participant where they were diagnosed and received care for their HIV/TB/COVID-19 and note this clinic in the **Participant Clinic Form.** For participants who we find to have HIV and/or TB, we will refer them to a Worcester clinic of their choice, and will note the clinic in the **Participant Clinic Form** in order to request the medical records to access the records later.

17.1 Medical Record Request Process

Medical records will be collected from local clinics to supplement information collected in the Medical History Survey and TB Diagnosis Data Collection Form. See table x below for details on how to collect and return medical records for abstraction.

Table 1: How to Request Medical Records for Record Abstraction

Clinic	How to collect records	How to return records	Contact Person and Phone Number
Worcester CDC	If participant was seen in the CDC TB room that day, study team can go to the TB room and fetch the participant's folder from Kobus (Aim 3 Arm 2)<br><br>For participants who did not attend the CDC clinic that day (Aim 1) we must email Kobus a list of participant's records we need on Wednesday. For each participant we must send their First Name, Last Name, and Date of Birth. We can then pick the	Study team takes the folders back and hands it over to Kobus personally. He then files it accordingly.	Willem (Kobus) Viljoen - willem.viljoen@westerncape.gov.za<br><br>Xolelwa Jack - xolelwa.jack@westerncape.gov.za<br><br>Jeanne Olivier -

	files up on Friday. The files must stay on the CDC grounds, so we must make the necessary scans at the TRUST pre-fab.		jeanne.olivier@westerncape.gov.za
Empilisweni Clinic	*TBD*	*TBD*	*TBD*
Worcester Hospital	*TBD*	*TBD*	*TBD*
Brewelskloof Hospital	*TBD*	*TBD*	*TBD*

17.2 Handling Medical Records

Medical records contain confidential medical information as well as participant identifiers. Participant medical records must always be handled with vigilance. Records should be transported from the study clinic to Ukwanda in a secure container with a snap lid in the boot of the car so that they are not visible. Medical records should be reviewed to identify the information necessary for TOTAL only. Staff should never review more of the record than needed to abstract the necessary HIV/TB/COVID-19 information. When staff identify the pages of the medical record containing the necessary information, they should copy these pages, redact any identifying information, and file the source document in the participant file. Once the necessary pages are copied, the team must ensure the record is organized and neat before returning it to the clinic. Medical records should never be out of the clinic for more than 6 hours.

18 Study Withdrawal Reasons

Premature termination from the study (defined as withdrawal prior to completion of any Aims enrolled into (signed consent for) may occur for the following reasons:

- The participant does not meet inclusion/exclusion criteria but was mistakenly enrolled (also a protocol deviation) *Note: this should not occur as we have created our screening tools to include checks that help to prevent this from happening.*

- The participant or participant’s parent/guardian elects to withdraw consent for all future study activities, at any time and for any reason

- The participant is deemed “lost to follow-up” (they can no longer be contacted for study participation)

  A participant will be considered lost to follow-up:

  Aim 1 and Aim 2

  If the study team is unable to make contact with the participant for **3 months.**

  Aim 3 Arm 1

If he/she does not return for their Aim 3 Arm 1 Visit within **one week of starting TB treatment.**

Aim 3 Arm 2

If he/she does not return for their Aim 3 2 Visit within **one week of starting TB treatment.**

- The participant dies
- The investigator no longer believes participation is in the best interest of the participant

Any time a participant is finished in the study, either after finishing all study activities, or being withdrawn early for the reasons above, an **End of Study Participation (ESP) CRF** must be completed.

19 Study Site, Staff, and Participant Safety and Security Procedures

We will ensure the safety of our sites, staff, and participants during study visits through a number of means. Detailed information on our safety and security procedures are found in **SOP: Safety and Security Procedures.**

20 Adverse Events (AE)

If a participant experiences any adverse events between signing the informed consent form (original signature date) and finishing in the study, the event will be recorded on the **Adverse Event Log.**

A medical history is taken during Aim 1 Visit 1 and Aim 3 Arm 2. As part of this history, it is important to probe for pre-existing conditions of any type. This baseline knowledge is necessary to determine if conditions experienced during the TOTAL study should be considered an adverse event. Pre-existing conditions should not be recorded on the Adverse Event Log, but they should be noted in the Medical History Survey.

20.1 Adverse Event Definition and Collection

A clinical adverse event (AE) is defined as any untoward or unfavourable medical occurrence associated with an individual's participation in the research, whether or not it is considered related to the participation in the research. In general, new conditions that arise during the study, or pre-existing conditions that worsen in severity or frequency, will be captured as AEs. We will also capture nonmedical events that may be behavioral (e.g., violence) or social (e.g., arrest, imprisonment) on the Adverse Event Log.

All adverse events will be recorded in the Adverse Event Log regardless of their relationship to study treatments or study procedures. In general, all adverse events, occurring after the participant has signed the informed consent form will be recorded until the time the participant completes or withdraws from the study.

Adverse events may be discovered through several means:

- Observing the participant during a visit
- Receiving an unsolicited complaint from the participant
- Reviewing abnormal values or results from clinical or laboratory evaluations

20.1.1 Documentation and Review Process (AE Form)

Each adverse event will be documented on the Adverse Event (AE) Log. The AE Log captures the text description of the event, severity grade, start and stop dates, whether the event is ongoing, whether the event is intermittent or continuous, the relationship to study participation and type of relationship, outcome of the event and any actions that were required to address the event. If the event is identified as a possible Serious Adverse Event (SAE) (severity grade 3 or higher according to the National Institute of Allergy and Infectious Diseases Division (DAIDS) Table for Grading the Severity of Adult and Paediatric Adverse Events version 2.0/November 2014)) an individual **SAE Form** must be filled out and sent to the Principal Investigator within **24 hours** of the study team becoming aware of the event. The SAE Form is a concurrent form that is not associated with any given study visit. It is a repeating form such that each event serves as its own record. This form also captures whether the event qualifies as an SAE that requires immediate reporting to the ethics committee (which is a severe event that is probably or definitely related to the individual's participation in the study) versus reporting at the annual continuing review. If the PI determines the SAE to be probably or definitely related to the participant's participation in the study, the SAE must be reported to the Stellenbosch and Boston Medical Center HREC/IRB within **24 hours of the PI's notification of the event** (See **Form: SAE Form**). See **Figure 3: Flow Diagram of Adverse Event Reporting** below for an overview of reporting procedures.

20.1.2 Relatedness to Study Participation

For this study, a related AE will include any untoward or unfavourable medical occurrence associated with:

- Study Mandated Procedures

  Any AE occurring within 24 hours after a study-mandated procedure (e.g., sputum induction, blood draw, questionnaires) that is possibly or definitely associated with the procedure.

The following clinical situations, when associated with study procedures, are defined as AEs and will be recorded on the AE form as related to the specific procedures. These situations do not limit the investigator from reporting any other events as AEs, whether they are associated with these procedures or not.

Blood Draws

- Fainting/vasovagal events
- Bruising at puncture site larger than 2 cm diameter
- Bleeding from puncture site lasting more than 5 minutes
- Swelling at puncture site larger than 2 cm

Sputum Induction

- Wheezing, coughing, or dyspnoea, which does not resolve within 30 minutes of the procedure.

The relationship of an AE to the study procedures is recorded on the AE Log as unrelated, possible, or definite relationship. The definitions of these categories follow:

- Unrelated: The AE is clearly not related; there is insufficient evidence to suggest a causal relationship

- Possible: The AE has a reasonable possibility to be related; there is evidence to suggest a causal relationship

- Definite: The AE is clearly related

The study nurse will initially determine the relatedness of the AE, and record it on the AE Log. The Principal Investigator will decide on the final determination of attribution for safety reporting.

20.1.3 Severity Grade Assignment

The severity of an adverse event (AE) describes its intensity. The severity of each TOTAL AE will be graded according to the criteria set forth in the National Institute of Allergy and Infectious Diseases Division (DAIDS) Table for Grading the Severity of Adult and Paediatric Adverse Events version 2.0/November 2014. The DAIDS document provides a common language to describe levels of severity, to analyse and interpret data, and to articulate the clinical significance of all adverse events. Using the DAIDS tables for safety data reporting ensures consistency in the evaluation of AEs across personnel. Adverse events will be graded on a scale from 1 – 5 according to the following standards in the DAIDS manual:

AE scale	DAIDS Severity Grade Description
Grade 1	Mild symptoms causing no or minimal interference with usual social and functional activities with intervention not indicated
Grade 2	Moderate symptoms causing greater than minimal interference with usual social and functional activities with intervention indicated
Grade 3	Severe symptoms causing inability to perform usual social and functional activities with intervention or hospitalization indicated
Grade 4	Potentially life-threatening symptoms causing inability to perform basic self-care functions with intervention indicated to prevent permanent impairment, persistent disability
Grade 5	Death

Specific AEs should be compared to the DAIDS table to determine severity grade. For example, using the table, if a participant has pain related to the blood draw that causes greater than minimal limitation of the use of his arm, but the pain does not prevent him to perform his usual activities, then his AE would be assigned a severity grade 2 (moderate).

If a specific event is not included in the DAIDS table or if staff is uncertain about the AE grade, staff should contact the study Principal Investigator, and the study Principal Investigator should use the general categories above and his/her clinical discretion to assign the appropriate severity grade.

20.1.4 Serious Adverse Events (SAE) (SAE Form)

Definition

An adverse event (AE) is considered “serious” if it is grade 3 or higher or in the view of either the Principal Investigator or Study Sponsor, it results in any of the following outcomes:

- Death
- A life-threatening event

  An AE is considered “life-threatening” if, in the view of either the investigator or Study Sponsor, its occurrence places the participant at immediate risk of death. This does not include an AE or SAR that, had it occurred in a more severe form, might have caused death.
- Inpatient hospitalization or prolongation of existing hospitalization

  Elective hospitalizations are not to be reported as serious adverse events (SAEs) unless hospitalization is prolonged due to complications.
- Persistent or significant incapacity or substantial disruption of the ability to conduct normal life functions
- Important medical events that may not result in death, be life threatening, or require hospitalization may be considered serious when, based upon appropriate medical judgment, they may jeopardize the study participant and may require medical or surgical intervention to prevent one of the outcomes listed above

SAE Reporting Procedures

Serious adverse events (SAEs) are reported on the AE Log and on an individual Serious Adverse Event (SAE) Form. The event will be classified as serious based on the responses to the AE Log responses and the definitions above. The staff member who completes the AE Log and individual SAE Form should also provide any relevant source documentation prior to scanning/ emailing the SAE form to the PIs (SU and BMC), SU Junior Scientist, and BMC Coordinator. The BMC PI must review and initial on all SAEs.

All adverse events deemed serious by the above definition must have an individual SAE form completed by a study nurse and this form emailed to the individuals listed above within 24 hours of the study team’s notification of the event. This holds for all serious events**, regardless of its’ expectedness or relationship to the study.** This also holds even if pertinent details are lacking at the time of the initial report. It is expected that updates will need to be made to the SAE forms and materials as further details of the event become available.

In the event that site personnel cannot establish a connection (example, no internet) to report SAE Forms, the Project Manager at BMC should be contacted via phone (WhatsApp) to report the event. If feasible, the related forms should be sent via secure e-mail or as pictures on WhatsApp to the Project Manager to expedite the reporting process to the PI. When the field team is back online, the appropriate logs and forms should be emailed to those individuals listed above, as soon as possible.

Expectedness

An AE is considered an “unexpected” event if the event is not listed at the specificity, severity or rate of occurrence that has been observed in the published literature; or is not consistent with the risk information described in the general investigational plan (protocol or informed consent document).

Coughing, coughing up blood, and chest pain or trouble breathing are common in individuals with TB. Participants identified as having TB at baseline, or through any of their Aim 1 sputum samples, who show the above symptoms, should not have their TB diagnosis or symptoms reported as AEs unless their symptoms increase from their baseline status during their time in the study. For example, the events of coughing or coughing up blood after being diagnosed with TB, would not be considered unexpected AEs, unless these symptoms were increased from their baseline status. *(For example: A participant is diagnosed with TB and has been experiencing shortness of breath. This symptom is not an AE and is not unexpected; however, if their shortness of breath increases during a study visit to where they need a bronchodilator or need to attend the clinic or hospital, this* ***is*** *an unexpected adverse event and must be reported on the AE Log, and pending severity, on an individual SAE form).*

If there is ever a doubt about whether an event should be reported as an AE, it is always safer to include the event on the AE Log. If there is ever a doubt about whether an AE should be classified as an SAE, it is always safer to complete an SAE Form and email it to the PI’s (SUN, BMC, SAMRC), Junior Scientist, and Boston Project Manager, and they will determine the appropriate course of action.

Figure 3: Flow Diagram of Adverse Event Reporting

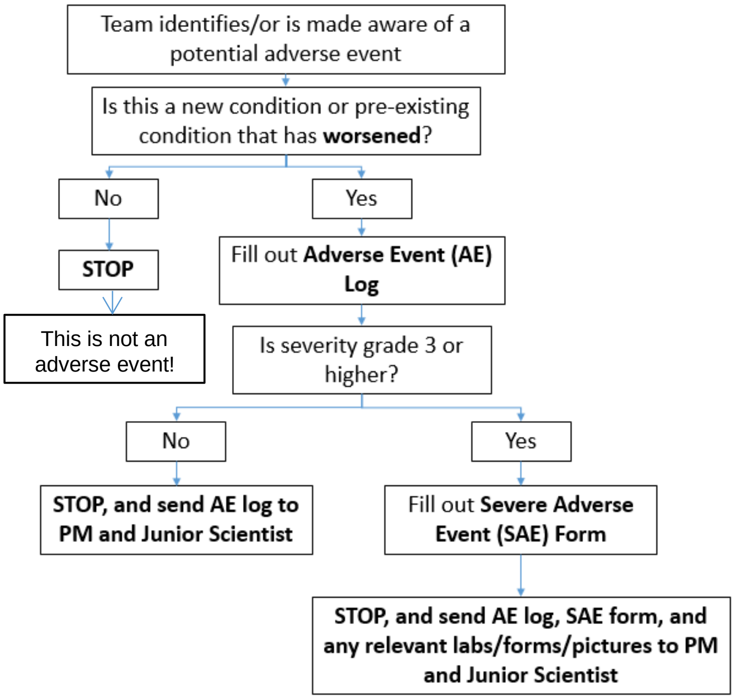

21 Protocol Deviations

A Protocol Deviation is as an unanticipated departure from approved protocol procedures, whereas anticipated departures are requested by the investigator as "protocol exceptions" on a one-time basis only. Protocol exception requests must be reviewed and approved by the IRB/REC prior to implementation.

The protocol includes, but is not limited to, such components as the INSPIR and sponsor's protocol, the recruitment procedures, subject eligibility assessments, the consent form and process, study interventions and any other information relating to the conduct of the study.

Minor protocol deviations are any unapproved changes in the research study design and/or procedures that do not have a major impact on the participant's rights, safety or well-being, or the reliability of the study data. Minor protocol deviations must be reported in aggregate to the IRB/REC at the time of continuing review. Minor protocol deviations are tracked in real time on the google sheet **titled "TOTAL Protocol Deviation Log".**

Major protocol deviations (protocol violations) are any unapproved changes in the research study design and/or procedures that may affect the participant's rights, safety or well-being, or the reliability of the study data. Major Protocol Deviations must be reported to the Boston Project Manager and SUN Junior Scientist within 24 hours of the team becoming aware of the event using the **Major Protocol Deviation/Violation Reporting Form**. Major protocol deviations must be reported to the IRB/REC within 5 days of the PI becoming aware of the event.

The criteria for defining major deviations include any of the following:

- The deviation has harmed, or posed a significant or substantive risk of harm to, the research participants.
- The deviation resulted in a change to any participant's clinical or emotional condition or status.
- The deviation has damaged the scientific completeness or soundness of the data collected for the study.
- The deviation is evidence of willful or knowing misconduct on the part of the investigator(s).
- The deviation involves serious or continuing noncompliance with federal, state or local regulations.

During the course of any study, unintentional changes may occasionally occur. Any change to the approved study that has not been reviewed and approved by the IRB prior to its initiation is a protocol deviation and must be logged on the Protocol Deviation Log. If deemed a major protocol deviation the event must be reported to the PI (BMC, SUN, SAMRC), BMC Project Manager, and SUN Junior Scientist as soon as the team is made aware of the event (within 24 hours).

The report of the incident on the Protocol Deviation Log must:

- Include a detailed description of what the deviation was, why it occurred and how extensive it was (how many subjects were affected).
- Include a corrective action plan (CAP) describing the steps that have been taken/will be taken to prevent a reoccurrence of the deviation in this or other studies
- Indicate whether or not the event resulted in an adverse event
- Indicate whether or not the event changed risk/benefit ratio of the study

Minor protocol deviations should be submitted to all institutions' IRBs/RECs in their respective formats at the annual continuing review.

21.1 Reportable Incidents: What Are Incidents?

Major (reportable) incidents are any problematic or unanticipated events involving the conduct of the study or participant participation that may occur during the course of the research project. Report all major incidents to the HRPP/IRB.

Examples

Some examples of study-related incidents include, but are not limited to:

- Receipt, and subsequent resolution by the study team, of a participant complaint regarding late study payment.
- Receipt of a significant complaint or concern from a potential or enrolled study participant, such as a complaint/concern that may adversely impact the participant's safety, rights or welfare. Reminder: Investigators are obligated to make a good faith effort to resolve any study-related concern or complaint they receive.
- Inappropriate behaviour of study participants and/or research personnel.
- Problems during study recruitment or the informed consent process.
- Problems with the study design in which a majority of participants have difficulty adhering to the study schedule of procedures.
- Potential breach of study participant's privacy or confidentiality (depending on extent, may be an adverse event)
- Withdrawal or significant reduction in resources necessary to adequately and safely conduct study activities.
- Changes to the protocol to eliminate or reduce an apparent immediate hazard to the safety of research participants or others

:

You must also report any unanticipated problem involving potential risk to participants or others. All incidents must be reported to PI as soon as the staff is made aware of the incident (within 24 hours), using the **Reportable Incident Form**, and the PIs must report all incidents within 48 hours of learning of the incident, to their respective IRB (SUN and BMC), using their IRB's preferred reporting system and format.

Minor (non-reportable) incidents are any events involving the conduct of the study or participant participation that may occur during the course of the research project but which is not problematic or involve significant potential to harm the participant(s) or others. Always report minor incidents to the BMC Project Manager and SUN Junior Scientist as soon as you are made aware using the **Reportable**

Incident Form. The BMC Project Manager in conjunction with the PI will determine whether the event is a major incident and needs immediate reporting.

22 TB Drug Resistance Incidental Findings

Through TOTAL *mtb* culturing, we may identify participants with drug resistant tuberculosis, particularly to INH, EMB, and/or PZA (as RIF resistance will likely be picked up on the NHLS Xpert Ultra testing and reporting). These findings will likely occur after a participant has started TB therapy. Drug resistance is a critical issue in tuberculosis care, and as such, we report incidental findings of resistance to the clinic the participant is receiving TB care at. The table below describes the reporting procedures for the clinics we anticipate our participants will receive care at.

Drug resistance results will only be transferred to clinic for those that Prof Warren's lab finds as having resistance. We do not need to provide information to clinic if a participant is found to be susceptible. As soon as we identify a participant as having drug resistance, a letter must be immediately drafted and transferred to the clinic. Participants are NOT to be told that we have found them to be drug resistant.

Drug resistance results will be written up in the form of a letter. The templates for these letters are saved in Dropbox > TOTAL Field Team > 'Letters to Clinic'. The bolded/highlighted fields should be updated to reflect the appropriate participant and relevant information.

Because the clinic does not recognize our participants by their PID number, we have to make sure identifiers are added back to each page that we give to clinic. This will include their first name and surname. All of these fields can be found in the Pre-Screening Log. This information is added directly into the letter template.

It is the duty of the clinic to determine what next steps are based on the information we provide.

A copy of the letters transferred to clinic should be added to the participant's consent form folder (remember, these files now have identifiers on them!). No electronic copy should be saved of these letters.

Table: Reporting Procedures for Incidental Findings of TB Drug Resistance

Clinic	How to Report	Contact Person and Phone Number
Worcester CDC	Complete TOTAL Resistance letter. Information is transferred directly to Sr Olivier. All pages transferred to her should be put in an envelope. Her name should be written on the outside of the envelope. This envelope should be handed directly to her. If she is unable to be located and is not in her office (her office should be unlocked), you should put the envelope in the bottom drawer of the small set of gray drawers located on her desk. Make sure the drawer is closed once you place results within it. She will routinely look in that drawer to check for results from us.	Person: Sr. Olivier<br><br>Phone:
Empilisweni Clinic	Complete TOTAL Resistance letter. Further procedures TBD.	Person:

		Phone:
Worcester Hospital	Complete TOTAL Resistance letter. Further procedures TBD.	Person:<br><br>Phone:
Brewelskloof Hospital	Complete TOTAL Resistance letter. Further procedures TBD.	Person:<br><br>Phone:

23 Consenting Guidelines

The informed consent process involves three key elements: (1) disclosing to potential participant sufficient information needed to make an informed decision; (2) facilitating the understanding of what has been disclosed; and (3) promoting the voluntariness of the decision about whether or not to participate in the research. Informed consent must be prospectively obtained. Informed consent is an active process between the study staff and the potential participant. Potential participants should be provided with ample opportunity to ask questions and seek clarification from the study staff. The potential participant should be in a position to freely decide whether to initially enroll in the research, or later, to withdraw or continue participating in the research. The informed consent process should ensure that all critical information about a study is completely disclosed, and that prospective subjects or their legally authorized representatives adequately understand the research so that they can make informed choices.

The process of informed consent should be conducted in a manner understandable to the study population, e.g., offering information in Afrikaans, isiXhosa, and/or English. For individuals 18 years or older, written informed consent must be obtained. For individuals younger than 18 years of age, written assent from the participant as well as informed consent from their parent or legal guardian must be obtained.

The informed consent process should include that the following elements be clearly conveyed to the potential participant:

1. A statement that the study involves research, an explanation of the purposes of the research and the expected duration of the participant's participation, a description of the procedures to be followed, and identification of any procedures which are experimental;
2. A description of any reasonably foreseeable risks or discomforts to the participant;
3. A description of any benefits to the participant or to others which may reasonably be expected from the research;
4. A disclosure of appropriate alternative procedures or courses of treatment, if any, that might be advantageous to the participant;
5. A statement describing the extent, if any, to which confidentiality of records identifying the participant will be maintained;
6. An explanation as to whether any compensation or medical treatments are available if injury occurs and, if so, what they consist of, or where further information may be obtained;
7. An explanation of whom to contact for answers to pertinent questions about the research and research participants' rights, and whom to contact in the event of a research-related injury to the subject; and

8. A statement that participation is voluntary, refusal to participate will involve no penalty or loss of benefits to which the participant is otherwise entitled, and the participant may discontinue participation at any time without penalty or loss of benefits to which the subject is otherwise entitled.

23.1 Documentation of informed consent

Each participant will be provided the copy of the consent form describing their involvement and the study will retain the signed copy of the informed consent form. These will be maintained as part of each participant's file of documents with personal identifiers in locked cabinets at the Ukwanda study site.

23.2 Additional considerations for illiterate participants

Assessment of literacy
Potential participants will be asked about their reading comprehension when provided the informed consent form. If they feel uncomfortable with the level of reading, the study nurse will find a suitable witness and then read and discuss the study and consent with the participant.

If a patient is found to be unable to read and articulate the informed consent form, the study nurse will inform the participant to return with a witness whom they feel comfortable with (and must understand the witness will know they consented to be in this study). The study nurse will then read and discuss the study and informed consent sheet with the potential participant and the witness will be asked to confirm that, to his/her knowledge, the participant understood what was discussed and was able to make an independent informed decision about their participation. Participants who are unable to write their name will be asked to provide an ink fingerprint on the signature line instead. The witness must also sign the consent form.

Assessing participants' comprehension of information communicated
The study nurse should make an effort to confirm that the participant understood all information provided to him/her. The study nurse should give the participant ample time to ask questions and take initiative to reframe any information he/she senses the participant did not adequately comprehend.

23.3 Confidentiality

All participant documents will be kept in locked cabinets at the Ukwanda study site. Each participant will have two folders dedicated to them that will be kept in separate locked locations. Documents that have personal identifiers (e.g., locator forms, ICFs) will be kept in one separate folder, while "everyday" documents that are de-identified with participant IDs will be kept in a separate folder in a different locked cabinet. It is critical that any documents that have identifying information are stored separately from study data. For more information on proper data storage, see the **Data Management and CQMP.**

24 TOTAL Visit Scheduler Calendar

For purposes of managing the daily activities at the Study Site, a Google calendar (called the TOTAL Visit Scheduler) has been set up to allow the TOTAL team to record upcoming scheduled visits in one place. The account under which the calendar lives is **total.scheduling1@gmail.com**. The Visit Scheduler calendar is to be used as described below.

Entering Visit Information

Once set-up, the TOTAL Visit Scheduler Calendar will appear under "Other calendars" on the left-hand side when viewing Google Calendar using the total.scheduling1@gmail.com login. If the Visit Scheduler is not showing, click on the downward arrow next to "Other calendars" to expand the list.

IMPORTANT NOTE: When adding visits to the shared calendar or editing existing information, be sure that only the TOTAL Visit Scheduler calendar is checked in the calendar list on the lefthand side. This ensures that information is being placed in the correct calendar. If multiple calendars are checked, information from all checked calendars will overlay on top of each other. Be sure ONLY the TOTAL Visit Scheduler calendar is being viewed, then enter/edit information accordingly.

Note, many participants will be walk-in's, and will not have a previously scheduled appointment. For all walk-in's the RA checking-in participants for that day must still add the participant to the Scheduler. It is at the discretion of the RA checking-in to determine whether the clinic visits and waiting room are too full for that day and if the participant should be scheduled for a future date. If that is the case the RA should schedule the participant, add them to the calendar, and give them a reminder card (See Section 7: Appointments: Scheduling and Confirming for more information)

When entering visit information into the shared calendar, the following guidelines should be followed to keep the calendar neat, readable and useful for its intended purpose:

• Enter information for the following key visits: AIM 1 VISIT 1, AIM 1 VISIT 2, TB RESULTS VISIT, AIM 2 VISIT, AIM 3 ARM 1 VISIT, AIM 3 ARM 2 VISIT, OTHER VISIT
• The following abbreviations and time-blocks are to be used for each visit on the calendar

- AIM 1 VISIT 1: AIM1V1
  (2 hours)
- AIM 1 VISIT 2: AIM1V2
  (1 hour)
- TB RESULTS VISIT: TBVISIT
  (30 minutes)
- AIM 2 VISIT: AIM2
  (2 hours)
- AIM 3 ARM 1 VISIT: AIM3A1
  (2 hours)
- AIM 3 ARM 2 VISIT: AIM3A2
  (4 hours)

The following information should accompany each log into the google calendar:

- Record whether the visit is a WALK IN or SCHEDULED
- Record the visit abbreviation, for example, "AIM1V1" followed by the participant's Screen ID or PID (if they have one)

- For example: AIM1V1: SC0001

(Richard is an Aim 1 V1 and does not yet have a PID, so put his SCREEN ID)

- If the participant is attending two visits (combined visit) on one day list the abbreviations with a – in between and followed by the participant's ID. For example: AIM1V2-AIM3A1: P0006
- If a participant is scheduled in advance, mark color yellow.
- After check-in record that participant is "in waiting room", and mark color blue.
- If participant is brought into a clinic room by the team, change status to "In progress, and study nurses name and RAs name" and mark color green
- When participant checks out, change status to completed, and mark color red.

Summary of Color Standardization:

Yellow = Scheduled (not yet arrived)
Blue = In waiting room
Green = In progress
Red= Completed

Figure 4: Example Daily View of TOTAL Visit Scheduler

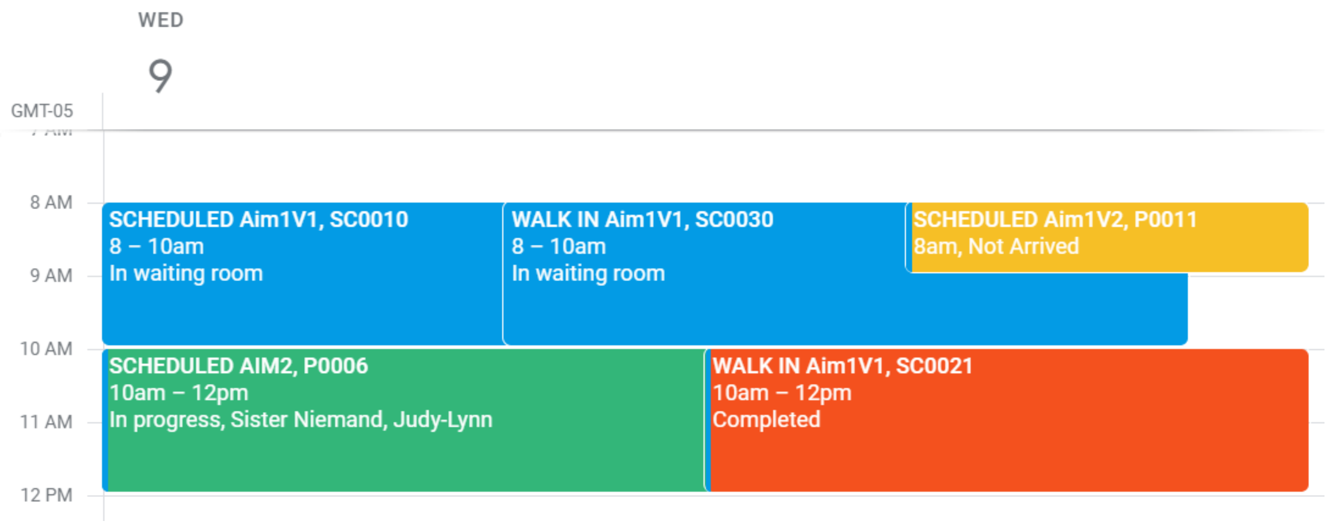

A **Visit Scheduler Cheat Sheet** is available and should be printed, laminated, and stored at the front desk to reference when adding participants to the Visit Scheduler.

25 Guidelines for Conducting Participant Interviews

All staff responsible for conducting the behavioural assessments will be trained to approach participants in a friendly, respectful manner so that they feel comfortable. This will help with collection of accurate data. At the beginning of the interview, the participant will be reminded of how long the interview will take to complete. Participants will be asked in which language they prefer the interview (isiXhosa, Afrikaans, or English) and the preferred language will be used. The interviewer must read the questions from the reference, paper version of the CRF in the language of choice, while filling out the correct responses on REDcap.

If a participant is confused about a question or task, the interviewer will be trained to stop and ask them if they understand. If the interviewer is still unsure of whether they understand or not, the participant will be asked to explain the question to the interviewer in their own words. Interviewers will be trained to identify inconsistencies in a response set. If a participant says one thing for one question, and then answers completely differently for another question on the same topic, the interviewer will stop and try to see if the participant understood the question. If necessary, the interviewer will go back to the previous question and check their answer. At certain times, participants may become less willing to answer questions, participate in the session or they may seem to just be giving an answer to finish quickly. Interviewers will be trained to pause the interview and ask the participant if they are alright or if they need a break. In addition, staff will be trained to read the questions exactly as they are written. If a participant changes their response, the staff member must update to the correct response on REDcap.

At the end of the interview, participants will be thanked for their time and for answering the questions. If appropriate for that appointment, they will be given their study voucher. They will also be reminded of upcoming study appointments and the expectations around those appointments will be explained to the participant.

26 Infection Control for Staff and Participants

Study participants, study staff, and hospital patients may be infectious and may transmit communicable diseases such as Tuberculosis, COVID-19, Hepatitis B, and HIV to each other and to study staff. The research environment needs to be as safe as possible; therefore, specific measures need to be taken to minimize the probability of disease transmission.

Infection control measures have 3 main purposes:
1. Prevention of disease transmission from participants to study staff
2. Prevention of transmission between participants
3. Prevention of transmission between participants and patients

Infection control measures are discussed in 4 sub-sections:
1. General measures, e.g., hand washing, gloves, masks, waste management
2. Physical cohorting
3. Specific procedures e.g. sputum collection, phlebotomy
4. Study staff occupational health screening

26.1 Risk classification of participants

Due to COVID-19, all staff, participants, and their samples will be treated as if they have COVID-19. See **SOP: Infection Control Guidelines for COVID-19 Pandemic** for details on all infection control measures to be taken by the field team under the current COVID-19 climate.

26.2 General infection control measures

26.2.1 Hand washing

Hand washing is the single most effective way to reduce risk of infection. It should always be performed in the following scenarios:
- Upon entering any study sites (study clinic, Ukwanda offices etc.)
- Before and after contact with a participant
- Before and after eating

- After using the toilet
- Before and after using gloves
- After contact with used equipment
- Immediately following contact with bodily fluids

Below is standard hand washing procedure:
1. Remove jewelry
2. Wet hands thoroughly all over
3. Use pH neutral soap and cold water
4. Lather soap all over hands
5. Rub hands together vigorously for 15-20 seconds. Pay particular attention to the fingertips, thumbs, wrists, finger webs and the backs of the hands
6. Rinse under running water
7. Pat hands dry with paper towels

26.2.2 Keeping skin intact
The best protection against bacteria is intact skin. Wounds, sores, cuts, or abrasions on the skin should be covered using a waterproof dressing. Care should be taken to avoid wounds on the hands and fingers in particular.

26.2.3 Gloves
Gloves should always be worn when expecting contact with bodily fluids or non-intact skin, e.g., participants with sores or skin lesions, or during sputum collection and phlebotomy procedures.

- For most scenarios on the TOTAL study sites, non-sterile latex gloves are adequate
- Check for holes or tears before using gloves
- Hand washing should be carried out before and after glove use as gloves may have pinholes, tears, or other defects
- Workers who develop an allergy or sensitivity to glove powder or contact with latex proteins need to advise their supervisor that they require alternative gloves
- Gloves should be used only once and discarded after use in the designated waste management containers

26.2.4 Masks
These requirements reflect updates to typical mask protocols due to COVID-19. Masks provide protection from inhalation of air-borne organisms and particulates. They are also worn to prevent dispersion of air-borne organisms from a potentially infectious participant or study staff member. Two types of masks are available on site: Surgical masks and N95 particulate masks.

- The surgical mask is more comfortable, can be worn for extended periods of time, and is effective in most low to medium risk scenarios (no patient interaction) to protect against inhalation of air-borne organisms.
- The N95 mask is more effective, but less comfortable and more expensive, and is used for moderate to high-risk scenarios, e.g., worn by staff around participants.

General measures for wearing masks:
- Cover the nose and the mouth securely
- Secure correctly behind the head with the provided fastenings
- Do not remove temporarily during use, e.g., for talking or sneezing

26.2.5 Protective garments and eye wear
Plastic aprons should be worn whenever there is a possibility of splashing of bodily fluids, e.g., handling of leaking specimen containers, contact with excretions. White lab coats should be worn in the lab to prevent contact with spilled specimens or reagents. A face shield/ goggles should be worn **at all times** during participant interaction.

26.3 Physical cohorting
Cohorting refers to the prevention of physical contact with potentially infectious individuals through control of the physical areas in which they are transported, where they wait, and where they undergo study visits and procedures. All participants should be considered potentially infectious. Individuals deemed high risk for transmission should be identified in a timely manner and appropriate actions should be taken.

26.3.1 Early identification and isolation of high risk participants
Contact should be minimized between participants with known tuberculosis and other participants. For participants with known, active tuberculosis:

- Schedule visits for a time when other participants are not booked
- Do not use the same waiting room or procedure room at the same time as other participants
- Transport the participant separately in the vehicle
- Ensure participant is wearing a surgical mask in the proper way

Participants will be screened before they enter the study clinic for COVID-19 Risk. Participants at high risk of having COVID-19 will be instructed to return to the clinic at a later date. See **SOP: Infection Control Guidelines for COVID-19 Pandemic** for more details on participant risk screening.

26.4 Safety protocol during specific procedures

26.4.1 Phlebotomy
- Staff must wear gloves and protective equipment
- Sharps must be properly discarded
- Staff must not recap needles
- The procedure area must be adequately lit
- The participant's arm must be kept still
- Specimen tubes must not be uncapped, unless study procedure indicates
- Only sealed and unbroken specimen tubes must be used

26.4.2 Sputum collection
- When a sputum specimen is being collected, the specimen should be collected in the sputum collection room.
- Staff should wear masks, gloves, a gown, and a face shield/goggles
- During the procedure, the staff member must stand behind and to the side of the participant
- The specimen jar should be closed tightly and properly

26.5 Screening of Study Staff for Illness

Staff will be screened daily for illness and COVID-19 risk. See **SOP: Infection Control Guidelines for COVID-19 Pandemic** for more information about staff screening, and procedures to follow if a staff

member is exposed, potentially exposed, or sick with COVID-19 or COVID-19 like illness.

26.5.1 Routine screening of staff

All existing staff employed in TOTAL will be required to undergo a routine symptom screen at least annually by SUN Occupational Health and will be required to have current hepatitis vaccination.

27 Participant Transport

Environmental factors that increase the risk for probability of transmission of *Mycobacterium tuberculosis* and/or COVID-19 within the vehicle transporting the study participant are: exposure to TB/COVID-19 in small, enclosed spaces, inadequate ventilation that results in insufficient dilution or removal of infectious droplets and recirculation of air containing infectious droplets.

See **SOP: Infection Control Guidelines for COVID-19** for details on the safe transport of patients to offsite facilities in order to protect the patient and driver of the vehicle from airborne disease transmission.

APPENDICES

Appendix I: Teaching Participants about Peer Recruitment

Teaching participants about the proper way to recruit peers is **critical** for the success of the study. Participants must understand who is a good candidate to recruit, and how to properly recruit them with the recruitment coupon.

Inform the candidate when explaining the recruitment process that:

- A “good” peer to recruit is someone the participant uses drugs with currently, who is 15 years or older and may be interested in, and available to, taking part in a study
- The participant must inform the recruit about the importance of the recruitment coupon, the candidate **cannot** enroll if they do not have the recruitment coupon
- Remind the participant that they will only be reimbursed for successfully enrolled peers, meaning the peer was in fact someone who uses drugs, and who was 15 or older and lives in Worcester area.
- Tell the participant they can come to the clinic with the candidate, and if the candidate successfully enrolls, the participant can pick up their recruitment at the same time.

The script below should be followed when describing the peer recruitment process to participants:

“Here are two coupons for you to use to recruit other people with whom you use tik or mandrax with. Please make sure that the persons you give the coupons to are 15 years are older, currently uses tik or mandrax, and has not received this coupon from someone else (i.e., has not participated in this study before). These persons can be friends, family or someone you live with, people you work with or study with, anyone you smoke tik or mandrax with.

Do not give any coupons to strangers when deciding who you should give the coupons to. Can you think of a few people right now to whom you can give your coupons to (don’t tell me their name, just think about them)?

Please inform your recruits that no one besides the study team will know they are in the study and explain to them that the information they provide is used for helping us understand how tuberculosis spreads. If that person accepts the coupon, show him/her the address and map where he/she can go to be interviewed and inform him/her that he/she can call the number on the coupon to make an appointment.

Please look at the coupon, it has the location of our clinic on it and this special number. This is the special number of the person you will give the coupon to. We use this number to track who gave coupons to who and can properly reimburse you for successfully recruiting your peers.

For each person you recruit who is eligible and takes part in the study, you will be given an incentive worth R50.

Once you give a coupon to one of your peers, and that peer takes part in the study and completes the interview, you can come back to the interview site to claim your compensation. You can also call the study phone number to check your incentive status (whether the person you recruited has enrolled and completed his interview). However, if this person does not take part in the study, we are not able to give you this incentive.

Only you can recruit peers with your coupons. If you have another person recruit peers for you, you will become ineligible to receive your incentive.

Thank you for your participation. Do you have any questions?"

Appendix II: Linkage to TB Care

Protocol for Referrals for further TB Testing and Care

Positive TB results (Xpert or MT Culture): All participants enrolled in the study who have a positive TB test result will be referred for further testing and care at the clinic of their choice (in the Worcester area). Participants will be given a ride to the clinic from their study visit if requested. It has been requested of us by the local clinics that we refer participants to the closest clinic to their home (i.e Avian Park container for those in Avian Park, Worcester CDC for those in the other neighborhoods, and Empilisweni Clinic for those from Zwelethemba).

For all TB referrals the research nurse will fill out a Referral Card and tell the participant to bring the card to the clinic and hand it to their treating nurse or physician (card template document name: "Referrals for TB" in the TOTAL dropbox folder). This card will contain the study contact number and the printed and signed name of the senior study nurse (or staff study nurse if senior nurse is unavailable). If the participant is positive on NHLS Xpert Ultra, the research team must also print the Xpert Ultra results from NHLS and staple them to the referral card for the participant to bring to the clinic (see **Figure 5: below for an example Referral for TB Care Card**). In all cases the referral card and attached documents but be put in a blank envelope (with no participant details on the outside), for the participant to bring to the clinic.

We will also include with this card an **instruction sheet** on how to book a visit at the local clinics, which includes the location and phone numbers to call to book these appointments. The referral card and

instruction sheet must be placed in a blank envelope, with no participant information, and given to the participant.

Figure 5: Referral for TB Care Card Template

Patient Referral

This participant of the TOTAL research study had a positive *mycobacterium tuberculosis* (MTB) culture and is being referred for further testing and care. Please contact the TOTAL research study with any additional questions

Research Team Phone Number: ______________________

____
TOTAL Research Nurse (Printed Name)

____
TOTAL Research Nurse (Signature and Date)

NOTE: In addition to the referral card, for referrals to the Worcester CDC, we must also email the results to TB Admin Officer Cobus Viljoen.

The results must be emailed using the template email "Email for TB referral to the Worcester CDC" in Dropbox.

Appendix III: Reactive HIV Test Results: Counselling and Linkage to Care

Core Principles of HIV Counselling and Testing in South Africa

1. HCT clients(s) and patient(s) must be provided with sufficient information about HIV counselling and testing, so that they can give their explicit and voluntary informed consent to receive services;
2. HCT services shall be confidential, meaning that anything discussed between the client(s) or patient(s) and the HCT provider may not be shared with other persons; except in cases where client's results are shared for client's medical benefit or when ordered by the court or law. Even in such instances, the clients should be informed that their HIV results will be shared.
3. HCT services must include accurate and sufficient client-centred counselling that addresses the needs and risks of the HCT clients(s) or patient(s) and the setting in which the services being rendered;
4. HCT services must adhere to national quality assurance guidelines for testing to ensure the provision of accurate and correct test results;
5. It is the responsibility of HCT programs and providers to ensure that HCT clients and patients are linked to care. This includes prevention, care and treatment and other clinical services, as

(From the RSA Department of Health HIV Counselling and Testing Policy Guidelines May 2015)

All participants who provide consent will receive pre-test counselling and onsite rapid screening for HIV, regardless of if they self-report HIV or not (**unless they have medical record confirmation of HIV**). All counselling and HIV testing is conducted by our HCT certified study nurse's **only.**

Pre and post-test counselling will be offered to all participants as they receive their HIV test results (see **HIV Pre-Post Test Counselling** Guide in Dropbox)

Make sure the participant understands what it means for their results to be reactive (positive) or non-reactive (negative). Negative is "No HIV present"; and Positive is "Yes, there is HIV present". If the two tests are in disagreement, this indicates an "Indeterminate" result, the test could not confirm whether there is HIV or not and the participant will need to provide a sample for a third, confirmatory test.

If you note that your participant is in distress, please follow the procedures in *Appendix V: Procedures for Identifying Distressed Participants and What to Do.*

To refer a participant for further care or testing at a medical clinic, complete and sign the Participant Referral Card (See Dropbox, Participant Referral Card (HIV Reactive) (See example below, **Figure 6: Referral for HIV Care Card Template).** The referral card will indicate that the participant has indication of HIV reactivity, and which tests were used. The card will also include the study team main contact number and must include the research nurses printed name and signature. Instruct the participant to bring the card with them to clinic to give to their treating nurse or physician. The card **will not include the participants name** for purposes of confidentiality, in order to reduce the risk of a lost card indicating a participant's name.

We will also include with this card an **instruction sheet** (See HIV and TB Referral Participant Instruction Sheet on dropbox) with details on how to book a visit at the local clinics, which includes the location and phone numbers to call to book these appointments. The referral card and instruction sheet must be placed in a blank envelope, with no participant information, and given to the participant.

It has been requested of us by the local clinics that we refer participants to the closest clinic to their home (i.e Avian Park container for those in Avian Park, Worcester CDC for those in the other neighborhoods, and Empilisweni Clinic for those from Zwelethemba).

Figure 6: Referral for HIV Care Card Template

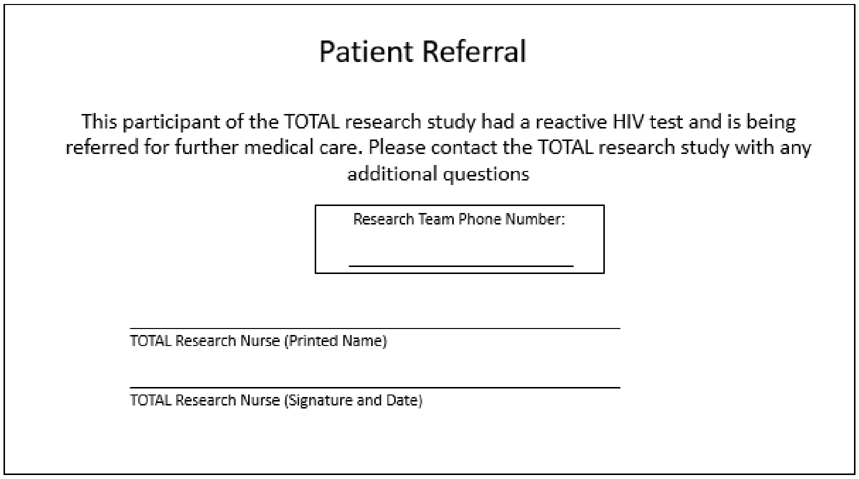

Patient Referral

This participant of the TOTAL research study had a reactive HIV test and is being referred for further medical care. Please contact the TOTAL research study with any additional questions

Research Team Phone Number: ______________________

____
TOTAL Research Nurse (Printed Name)

____
TOTAL Research Nurse (Signature and Date)

Appendix IV: Referrals for Substance Use

Protocol for Referrals to Counselling Services for Substance Use

<u>Illicit Substance Use</u>: All participants enrolled in the study who use illicit substances will offered be referrals to the counselling services listed in **Table 2** below. They will also receive the **TOTAL Patient Pamphlet** at their visit which lists these services, their locations, and contact information. Participants will be given a ride to counselling services from their study visit if requested.

If a participant is interested in a referral the research nurse will fill out a Referral Card (See **Figure 7** below) and tell the participant to bring the card to the counselling center and hand it to the counselling staff (card template document name: “Referral for Substance Use Counselling” in the TOTAL dropbox folder). This card will contain the study contact number and the printed and signed name of the senior study nurse (or staff study nurse if senior nurse is unavailable).

Figure 7: Referral Card for Substance Use Counselling

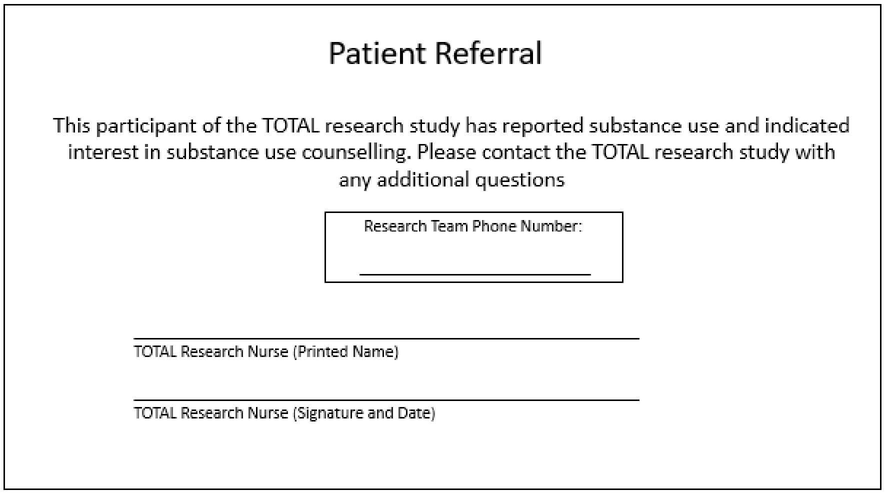

Patient Referral

This participant of the TOTAL research study has reported substance use and indicated interest in substance use counselling. Please contact the TOTAL research study with any additional questions

Research Team Phone Number: ______________________

____
TOTAL Research Nurse (Printed Name)

____
TOTAL Research Nurse (Signature and Date)

Table 2: Mental Health and Counselling Services Available in the Greater Worcester area.

Counselling and Emergency services	Address	Contact Details
**Toevlug Community Centre**	40 Noble Street, Worcester, South Africa	023 342 1162
**Bowl Community Centre**	62 High Street, Jacrens Building, 1st Floor, Worcester	023 342 1856
**Alcoholics Anonymous Meetings**	Church Hall, Church Street, Worcester<br>Thursday 19:00	086 1435 722 (National helpline)
**Department of Social Development**		080 0220 250 (Toll-free)<br>023 348 5300 (Regional office)
**South African Police services**		086 0010 111
**Worcester Ambulance services**		023 341 0177<br>10177 (Toll-free)
**Western Cape Government Safe line**		080 0035 553

Appendix V: Procedures for Identifying Distressed Participants and What to Do

Procedures for Distressed Participants

Given the nature of this study, there may be instances, where you (the study nurse/ interviewer) are faced with a distressed participant who you are concerned needs additional or immediate mental health support. For the most part, you can address this by talking to the participant and providing them with a referral to see the mental health nurse or for additional mental health services.

1. What is a distressed participant?

By “distressed” we mean that a participant shows signs of experiencing negative emotions and by his/her words, expressions, or other nonverbal behaviors or body language indicates that s/he is emotionally upset and may be showing signs of suicidal ideation.

<u>Possible signs or indications of distress:</u>

Please be aware of the following signs during all components of the study visit, however be particularly aware of responses and signs when administering the surveys

- A participant who is tearful and/or reports that s/he feels badly or is sad
- A participant who shows signs of being considerably more nervous or anxious (e.g., very nervous speech, increased sweating, difficulty sitting still during the appointment) than would be expected during an appointment.

- A participant who seems agitated or aggressive and that you cannot easily calm down.

1.1 Steps to follow with mildly distressed participants who need additional services

In most cases of distress, participants may talk about feeling badly but you will be able to manage or contain these feelings and continue with the appointment. They may however, need additional services or support once the counselling intervention has been completed.

In these cases, complete the following steps at the end of the study appointment:

- Indicate to the person that they seem upset or anxious
- Discuss with the participant the importance of additional support
- Ask if they would like to receive additional services to talk more about feelings that came up during the course of the interviews
- If the participant indicates s/he would like to talk with someone further, ask if they would like you to refer them to an organization
- Complete a referral form
- With the participant's permission, call the agency or the mental health nurse to make an appointment
- Provide the participant with a copy of the resource guide for additional services
- If the participant indicates that s/he does not want you to make a direct referral, suggest various organizations for him/her to contact on her own and give them a copy of the resource guide.

These steps should only be followed if the participant shows signs of distress or expresses a need for more counselling- but ***no suicidal intent or imminent harm is disclosed or suspected***.

If the person expresses any desire to end their life or hurt themselves or others, follow the steps described in 1.2 and the mandatory reporting procedures described in section 2.

Steps to follow with very distressed participants

There may be instances where participants are so distressed that you are concerned for their safety and may not be able to continue with the appointment. If participants express the intent to harm themselves or others, or ongoing abuse and/or neglect of children, then we have a duty to report this matter to the proper authorities, e.g. the police, courts or social workers. Field staff will provide information on toll-free hotlines that people can call to talk to someone and seek assistance, as well as local resource information.

If during the course of the study you are concerned that a participant intends to harm him/herself or others, then mandatory reporting procedures need to be followed on completion of the appointment. Immediately inform the project manager and the principal investigator (PI) who will report the incident to the various ethics committees and our project officer within 48 hours.

In these instances, please follow the following steps:

- As far as possible, complete the study appointment
- Indicate to the person that they seem upset or anxious
- Remind the person of the limits of confidentiality and your duty to report
- Follow the mandatory reporting procedures described below
- Complete the referral and release form

- Call the agency or the mental health nurse to make an immediate appointment
- Provide the patients with a copy of the referral letter in an envelope and keep one in the folder
- Provide the patient with a copy of the resource guide for additional services
- Contact the project manager and PI who will report the incident to the relevant regulatory bodies.

2. Mandatory reporting procedures

It is possible that a participant will indicate during the course of a discussion that s/he is in immediate danger of harm or poses a threat to the safety of others. We want you to feel that you can handle this situation with confidence.

There are essentially three situations in which there may be immediate danger of harm:

1) **Suicidal Intent:** The participant expresses a desire to hurt or kill themselves.
2) **Hurtful Intent to Others:** The participant expresses an interest in hurting or killing someone else (not necessarily someone living in the household).
3) **Child Abuse and Neglect:** Ongoing abuse and/or neglect of children.

The rest of this protocol describes what to do in each of these cases. For each of these cases, you must follow compulsory or mandatory reporting procedures.

These procedures are important to follow because they take away the responsibility of you having to decide what to do. These procedures are in your best interest and in the best interest of the participant- they are to keep the participant safe and unharmed.

2.1 Suicidal Intent

If a participant expresses an interest in harming or killing him/herself, assess whether the risk is immediate or not immediate by following this procedure:

Ask the following questions:

	**In The Past Month**	
Answer Questions 1 and 2	**YES**	**NO**
**1)** ***Have you wished you were dead or wished you could go to sleep and not wake up?***		
**2)** ***Have you actually had any thoughts about killing yourself?***		
If **YES** to 2, answer questions 3, 4, 5, and 6. If **NO** to 2, go directly to question 6		
**3)** ***Have you thought about how you might do this?***		

**4)** ***Have you had any intention of acting on these thoughts of killing yourself, as opposed to you have the thoughts but you definitely would not act on them?***		
**5)** ***Have you started to work out or worked out the details of how to kill yourself?***<br>***Do you intend to carry out this plan?***		
	**In the Past 3 Months**	
**6)** ***Have you done anything, started to do anything, or prepared to do anything to end your life?***<br>Examples: Collected pills, obtained a gun, gave away valuables, wrote a will or suicide note, took out pills but didn't swallow any, held a gun but changed your mind or it was grabbed from your hand, went to the roof but didn't jump; or actually took pills, tried to shoot yourself, cut yourself, tried to hang yourself, etc.<br>***In your entire lifetime, how many times have you done any of these things?***		

If the person answers YES to either questions 4, 5, or 6, this is a red flag and you should consider them at high risk for suicide. Risk is not immediate if the person is expressing some suicidal thoughts to describe how badly they feel but they do not have a plan. In the instance of high risk, follow the script below:

2.1.1 Procedure: IMMEDIATE or HIGH RISK of SUICIDE

If you assess that the participant is at **immediate risk** to him/herself, read the following script to the participant:

Script: Risk of participant suicide or self-injury (immediate danger of harm)

**Team member reads:**
"When you agreed to participate in this study, I promised that I would tell someone what you told me only if it was necessary to protect you or other people. You told me earlier that you were thinking of harming yourself. I suggest we contact an organization called LifeLine or SADAG and let them know so they can talk to you about how you feel. LifeLine and SADAG offers counseling. You can discuss your problem with one of their counsellors and they may be able to help you. Do you want to call them or should I call them for you?"

Willing participant:

- If the participant agrees to contact Lifeline or SADAG, use the project phone to make the call for the participant, hand them the phone and go to another room to give them privacy during the call.
- If the participant asks you to make the call on their behalf, use the project phone to make the call for the participant, and follow the script when speaking to the counsellor
- After the script has been read, hand the phone to the participant and go to another room to give them privacy in the call.

Script: Risk of participant suicide or self-injury (immediate danger of harm)

Team member reads to telephone counsellor

"We are conducting a research study and during an appointment, the participant expressed s/he was thinking of killing or harming him/herself. The participant has requested that we make this call for him/her.

Please note that this information was obtained through their participation in this research study. We went through appropriate informed consent, including telling the participant that a report might be made if the information s/he provided raised concerns about his/her well-being. I can give you additional information about the research study, if you would like. I can also provide you with the participant's name." Would you talk with him/her?

Unwilling participant

- If the participant is unwilling to contact the crisis hotline and doesn't want you to contact them, then you should immediately contact the project manager or Prof Myers who can assess the situation (e.g., determine reasons why the participant is unwilling to contact the hotline so these issues can be addressed) and advise of further inputs.
- You can also contact the family physician, medical officer and/or mental health nurse at the facility for their guidance.
- In the meantime, do not leave the participant by him/herself nor let them leave on their own, as they pose a risk to themselves.
- Ask the participant if s/he has a close family member or friend who can come to sit with them until you receive clear instructions about what to do.
- If the participant remains unwilling to contact the hotline, provide the participant with the referral guide as well as the counselling hotline numbers.
- Make sure you document who you spoke to and the advice provided on the incident form

Script: Risk of participant suicide or self-injury (immediate danger of harm - unwilling to contact hotline)

Read to participant

"You told me earlier that you had thought about harming yourself, and this concerns me. I have to report this information to the appropriate authorities. I can also take you to the mental health nurse, refer you to the family physician or call LifeLine or SADAG on your behalf. I strongly suggest that you contact LifeLine or SADAG or go and speak to a doctor. Here is information about the telephone hotline that you could call to discuss your problem with a counselor. I can refer you now to the mental health nurse serving this clinic. If you feel that this is an emergency now or later, you should go to a hospital emergency room right away. If you are unable to get to an emergency room without help, you should call the police for assistance."

After giving this information to the participant, immediately contact the attending family physician or mental health nurse and adhere to the following script. The physician will advise you on next steps.

Script: Risk of participant suicide or self-injury– to family physician

"During an appointment for project TOTAL, a participant expressed s/he was thinking of killing or harming themselves. The participant was encouraged to call LifeLine or SADAG and seek further professional assistance, but we also informed the participant that we have to report the situation. The participant was unwilling to contact anyone for help nor allow us to contact anyone on his/her behalf. S/he is with us now, but we are concerned about her safety.

Please note that this information was obtained through their participation in the study. We went through appropriate informed consent procedures. I can provide you with the participant's name and residential area. Please can you advise us as to next steps to follow?

2.2. Hurtful Intent to Others

It is extremely unlikely, but someone may spontaneously tell you that they are planning to seriously hurt or kill someone else. If a participant expresses an interest in harming or killing someone else, you should take the following steps.

- As far as possible, complete the study appointment
- Indicate to the person that they seem upset
- If they are expressing a desire to hurt someone else, explore whether there is an immediate risk (i.e. they have a plan and the means to do it)
- If there is immediacy and a plan, remind the person of the limits of confidentiality as expressed in the consent form, and your duty to report.
- Follow the mandatory reporting procedures described below
- Follow the reporting procedures - complete an incident report form and immediately send to the project manager and PI who will both keep copies on file.

2.2.1 NO IMMEDIATE RISK

If no immediate danger is perceived, advise the project manager and the PI immediately who will evaluate the seriousness of the issue and give advice on what to do. If the danger of harm is definitely credible, then the authorities will be called to report the incident. If the danger of harm is believed to definitely be credible, you may be advised to contact the police. In this case, please follow the script below.

Script: Risk of someone being harmed or killed revealed by participant

Team member to read to police

"We are conducting a research study, and during an appointment, a participant expressed an interest in harming or killing someone. I have discussed this situation with my supervisors and we have decided that this person might pose a real threat, so we are alerting you.

Please note that this information was obtained during an appointment that was conducted for this research study. We went through appropriate informed consent procedures. I can give you additional information about this research study, if you would like. I can also provide you with the contact information for the participant."

2.2.2 IMMEDIATE RISK

If immediate danger is perceived please alert the project manager and the PI immediately and they will report the incident to the authorities. Do not let the participant leave before you have instructions from the police or the trial manager as to next steps to take. Please follow each of these steps.

Script: Risk of someone being harmed or killed revealed by participant during the appointment

Trial manager or PI to read to the authorities

"We are conducting a research study, and during an appointment, a participant expressed that s/he intended to harm or kill someone, so we are alerting you.

Please note that this information was obtained during an appointment that was conducted for this research study. We went through appropriate informed consent procedures. I can give you additional information about the research study, if you would like. The participant is here at the moment."

2.3 Child Abuse/Neglect

Someone may tell you that s/he has abused or neglected a child. If a participant reports this, you must report the incident to the police (if sexual abuse), or Childline (if any other type of abuse) after completing the appointment.

Follow these steps:

- As far as possible, complete the study appointment
- Indicate to the person that they seem upset
- If they are reported abusing a child, remind the person of the limits of confidentiality as expressed in the consent form, and your duty to report
- Follow the mandatory reporting procedures described below
- Follow the reporting procedures - immediately contact the project manager and PI who will both keep copies on file.

2.3.1 PARTICIPANT ABUSING OR NEGLECTING A CHILD

Script: Child being abused or neglected by participant revealed

Read to authorities:

"We are conducting a research study, and during an appointment with a participant, certain indications were made that the participant has possibly physically or sexually abused or neglected a minor. This led us to conclude that it was necessary to contact you to alert you of our concerns.

Please note that this information was obtained through the participant's participation in this research study, with appropriate informed consent procedures. I can give you additional information about this research study, if you would like. I can also provide you with the contact information for the participant."

Appendix VI: Utilizing the REDCap Database

Every attempt should be made to collect data on REDCap while connected to wi-fi. If wi-fi is unavailable, data can be collected using the REDCap mobile App.

REDCap users can collect their REDCap data in a mobile app on an iPhone, iPad, or Android phone or tablet. The REDCap Mobile App adds a new dimension to REDCap's versatility by providing users with a tool for offline data collection, particularly in environments with poor Internet connectivity. The app cannot be used on its own but is a companion app that must be used alongside REDCap itself (you must first be a REDCap user at a REDCap partner institution before you can utilize the mobile app).

Appendix VI.I: How to Use REDCap Mobile App

Purpose

The primary purpose of the REDCap Mobile App is offline data collection. It is NOT intended to be used by participants in order to take a survey. Your project must fit one of the following scenarios:

- It is a project that needs data collected when there is no internet access available.
- It is a project that needs data collected when there is sporadic internet access.

Mobile App Account Setup
Your first step is to download the REDCap Mobile App from the App Store or Google Play Store. When you first launch the REDCap mobile app, you must agree to the terms.

The next step is to create two users:
1) Admin Account: This account will grant app function rights for data collectors which will allow them to setup/remove projects, send data, delete records, view/modify existing records and reset passwords. The admin account can also perform all of these functions with the exception of data collection. The admin account will not have any projects associated. The data collector passwords can be recovered via the admin account but the Admin password cannot be reset.
2) Data Collection: Allows a user to setup/remove projects, send data, delete records and view/modify existing records. The user will have their normal login for the REDCap server and a separate unique login for the REDCap Mobile App.

Enable User Rights for Mobile App
Once you have set up your users, you can then set up your mobile project. To set up your project, open your project on the external REDCap and navigate to User Rights and grant Mobile App Rights.

Request Mobile App API Token
Log into REDCap and navigate to your project. Click on REDCap Mobile App and then click on Request API Token. An email will be sent to REDCap Support for approval and you will be notified via email when your request is approved.

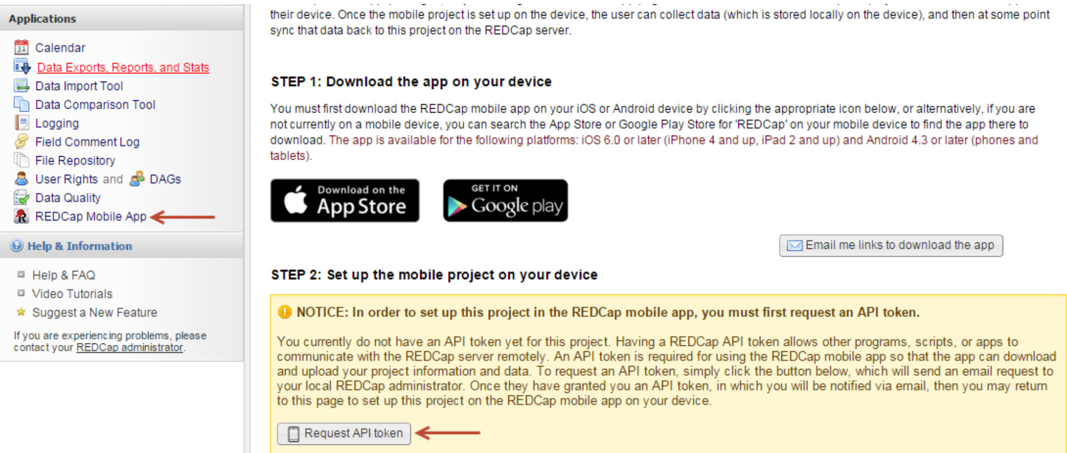

<u>Set Up Mobile Project on Device</u>

Launch the REDCap mobile app and log in as your data collection user (not the admin account) and click “Set Up Mobile Project”. Click “Scan QR code” from the REDCap Mobile App screen in your REDCap project and use your mobile device to scan the code. If you experience any issues you may click “Can’t get the QR code to work” and then enter the Initialization code in the code box on your mobile project.

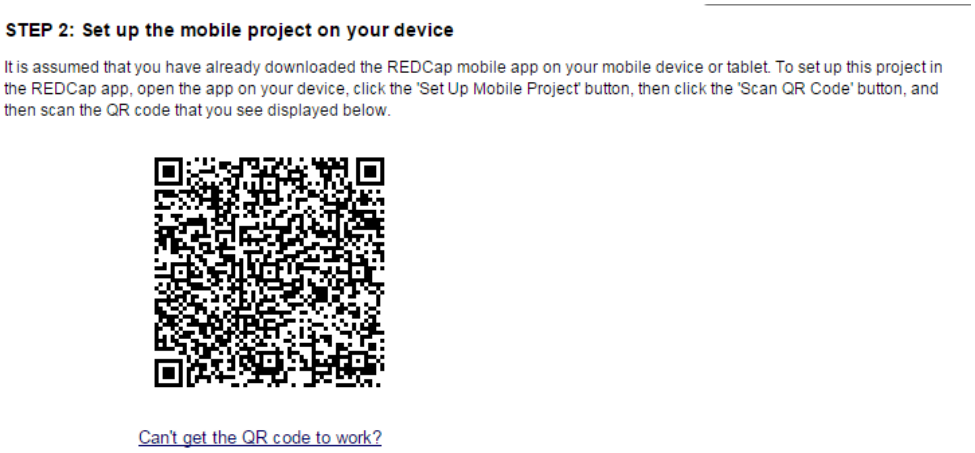

Click on the project name to confirm and to begin its download configuration. The download may take a few minutes depending on how large your project is. There are additional settings you may choose such as Download all records, get a partial set of data or do not download records.

<u>Collect Data</u>

To begin collecting data offline, click collect data. Select an instrument and then either click “Create New Record” or click on an existing record. Enter the data and then click Save Record or Save and Continue or Save and go to Next Instrument.

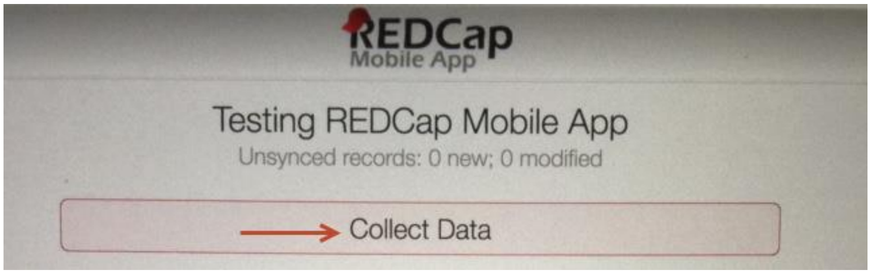

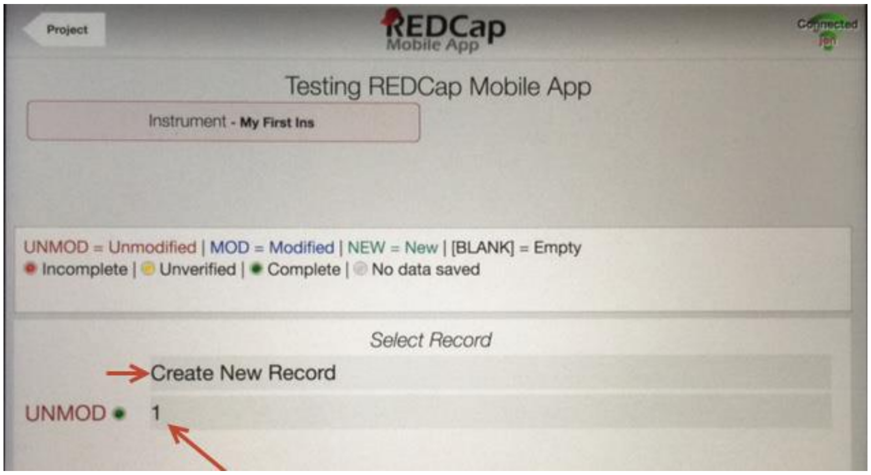

Check for Other Records on the Server

To check to see if there are records that exist in your project that do not exist on your mobile device, click “Check for other records on the server”.

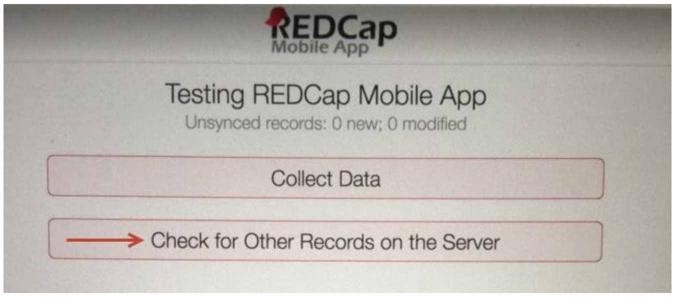

Send Data to Server

When you have an internet connect and you are ready to send your offline data to the REDCap server, click “Send Data to Server” and then click “Begin Send”. You will receive a message letting you know if your data was successfully sent to the REDCap server. Check your project on the REDCap server to ensure data was transferred correctly.

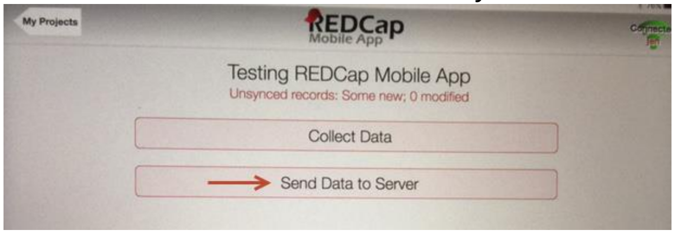

Refresh Setup & Data

If records exist in your project and you would like to sync them to your mobile device, click “Refresh Setup and Data”. This will delete your local copy of the project data and configurations as well as any

offline data you have not transferred over to the project on the REDCap server. Be sure to transfer your offline data first! Once deleted, the mobile app will install the current version. There will be two messages asking you to confirm that you want to delete your data.

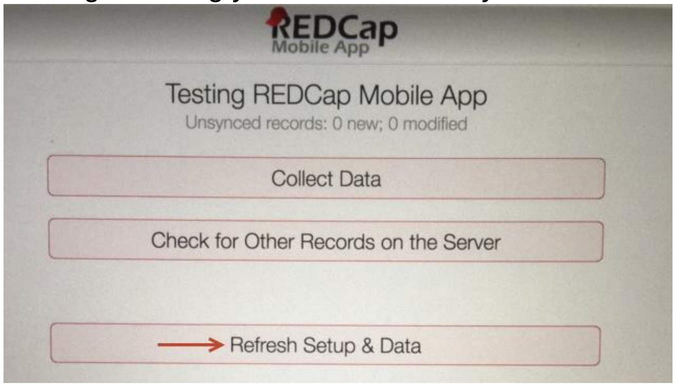

Project Admin

If you would like to delete the data from your Mobile Project, click “Clean All Project Data”. This will only delete the records on your Mobile App and will not affect your project on the REDCap server.
If you would like to remove the Mobile Project from your device, click on “Remove Mobile Project”. You will receive a message asking you to confirm that you want to delete the mobile project. This will not delete your project on the REDCap server.

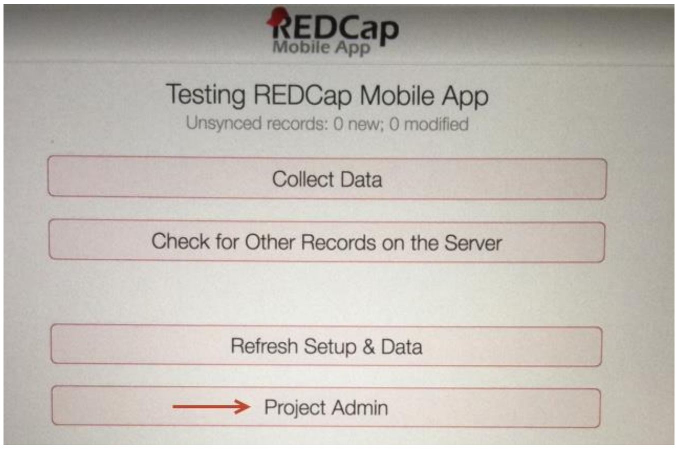

Send Emergency Data Dump

If your data was not transferred over successfully when you clicked “Send Data to Server”, you may send an emergency data dump. This should be used rarely and you must have an internet connection in order to use this functionality. The REDCap mobile app will send your data as a CSV file and it will be stored under the REDCap Mobile App section. To use this function, click “Send Emergency Data Dump” and then click “Proceed with Emergency Send”.

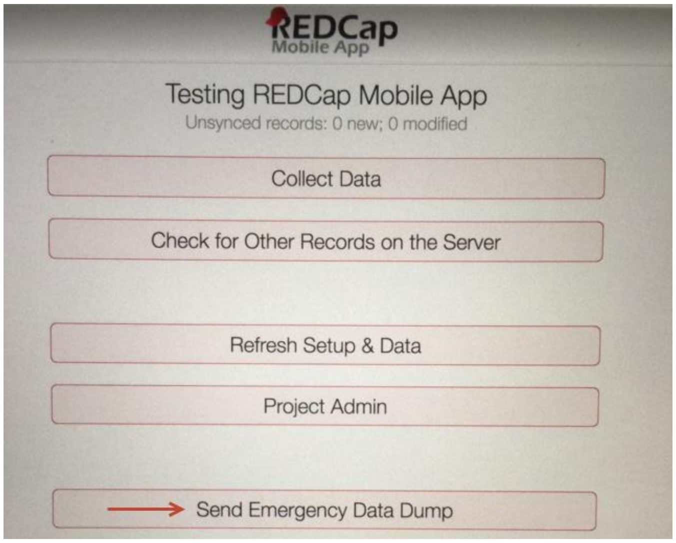

You will receive a message letting you know if your data dump was successful. Navigate to your project on the REDCap server and ensure the CSV file appears under the REDCap Mobile App section under the Mobile App File Archive tab.

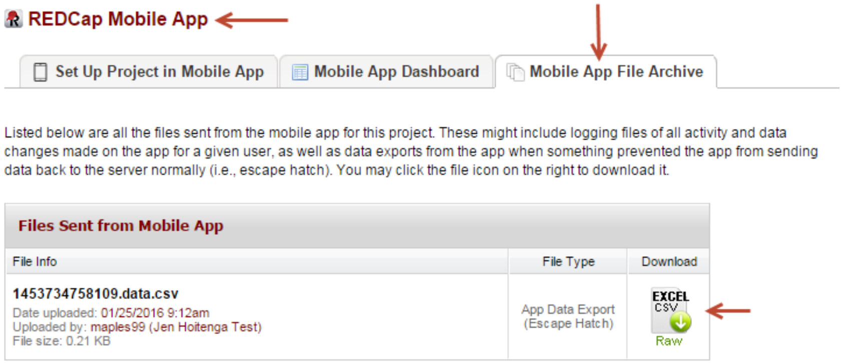

Click on the Excel CSV file and save it to your desktop. Click on the Data Import Tool and navigate to your CSV file and import your data.

<u>Send Project Logs</u>
If you would like to send the mobile app project log to the project on the REDCap server, click “Send Project Logs”. A text file will be sent and stored under the REDCap Mobile App section under the Mobile App File Archive.

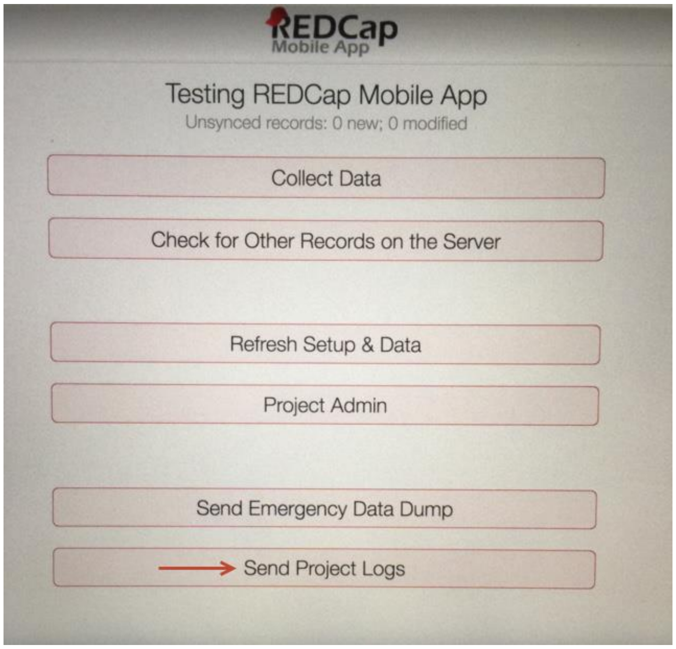

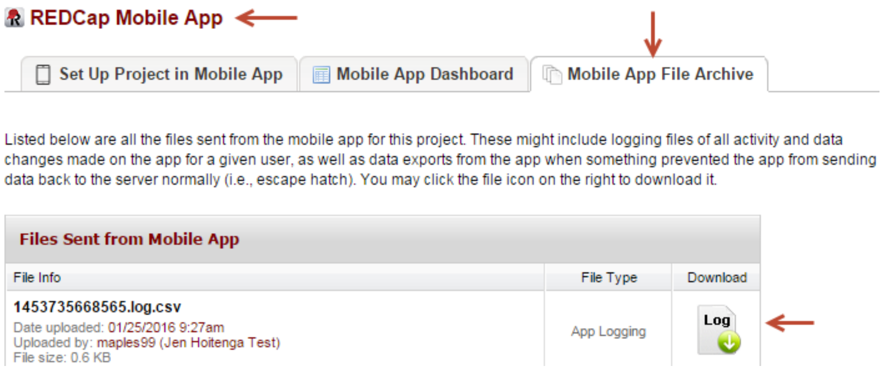

Appendix VI.II: REDCap Mobile App Troubleshooting

- *What happens if the app hangs during this process?* Consider using the Emergency Data Dump. Email rchelp@bu.edu as well for help. If you detect a bug in this process, do the following:
  26 Logout.
  27 Login with your normal PIN, only add a '00' to the end. This will make it an 8-digit PIN.
  28 This throws the app into Testing Mode. There is a button to submit a diagnostic report at the bottom of each screen.
  29 Reproduce your bug in Send Data if you can.
  30 Press the button at the bottom of the screen.
  31 Send a Bug Report via the main menu of the app or by emailing rchelp@bu.edu. We need a normal-language story of what occurred around the bug in order to make the diagnostic reports make sense.

- *What happens if the app totally crashes during this process?* You've probably run out of memory to some degree. The app only has a certain amount of memory from the operating systems. Try an Emergency Data Dump, which uses less memory.
